# New Inhibitors of Respiratory Syncytial Virus (RSV) Replication Based on Monoterpene-Substituted Arylcoumarins

**DOI:** 10.3390/molecules28062673

**Published:** 2023-03-15

**Authors:** Tatyana M. Khomenko, Anna A. Shtro, Anastasia V. Galochkina, Yulia V. Nikolaeva, Anzhelika V. Garshinina, Sophia S. Borisevich, Dina V. Korchagina, Konstantin P. Volcho, Nariman F. Salakhutdinov

**Affiliations:** 1 Department of Medicinal Chemistry, N.N. Vorozhtsov Novosibirsk Institute of Organic Chemistry, Acad. Lavrentjev Ave. 9, 630090 Novosibirsk, Russia; 2 Laboratory of Chemotherapy for Viral Infections, Smorodintsev Research Intitute of Influenza, 197376 Saint-Petersburg, Russia; 3 Laboratory of Physical Chemistry, Ufa Chemistry Institute of the Ufa Federal Research Center, 71 Octyabrya pr., 450054 Ufa, Russia; 4 Institute of Cyber Intelligence Systems, National Research Nuclear University MEPhI, 115409 Moscow, Russia

**Keywords:** coumarin, terpene, antiviral activity, cytotoxicity, respiratory syncytial virus, molecular modeling, F protein

## Abstract

Respiratory syncytial virus (RSV) causes annual epidemics of respiratory infection. Usually harmless to adults, the RSV infection can be dangerous to children under 3 years of age and elderly people over 65 years of age, often causing serious problems, even death. At present, there are no vaccines and specific chemotherapeutic agents for the treatment of this disease, so the search for low-molecular weight compounds to combat RSV is a challenge. In this work, we have shown, for the first time, that monoterpene-substituted arylcoumarins are efficient RSV replication inhibitors at low micromolar concentrations. The most active compound has a selectivity index of about 200 and acts most effectively at the early stages of infection. The F protein of RSV is a potential target for these compounds, which is also confirmed by molecular docking and molecular dynamics simulation data.

## 1. Introduction

Respiratory syncytial virus (RSV) belongs to the *Pneumoviridae* family and causes respiratory infections responsible for annual epidemics during the cold season. Having a high level of clinical manifestation severity (severe bronchiolitis and pneumonia, which may lead to death) in babies and young children, RSV infection is a significant medical and social problem nowadays [1]. Risk groups for RSV infection are also people over 65 years of age and patients with weakened immunity [2].

**Figure 1:**
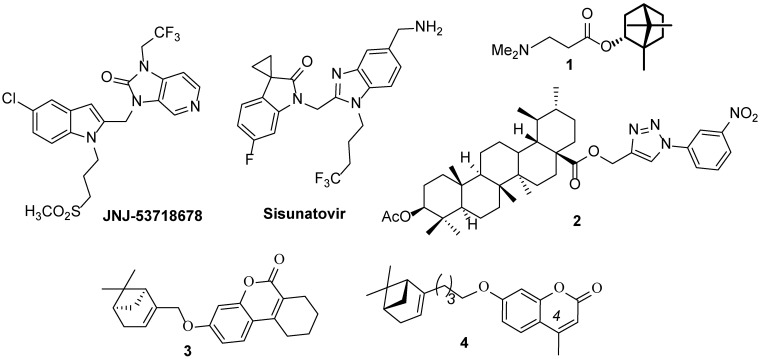
Known compounds with anti-RSV activity.

There is no vaccine against RSV infection, and therapy is usually symptomatic, with only one poorly effective antiviral agent Ribavirin [3,4]. Another antiviral drug palivizumab (a monoclonal antibody against the F protein of RSV) is approved not for treatment, but for prophylaxis only [5]. The F protein is essential for viral entry into the host cell and is considered to be an important target for therapy with low-molecular weight inhibitors, such as JNJ-53718678 [6] and sisunatovir [7] (Figure 1), which are under the second phase of clinical trials. Recently, monoterpenoid (–)-borneol and triterpenoid ursolic acid-derived esters **1** [8] and **2** [9] have been found to exhibit significant antiviral activity against RSV.

Previously, we demonstrated that compounds comprising coumarin and monoterpenoid moieties, such as compounds **3** and **4** (Figure 1), were highly active against RSV. In this case, the structure and absolute configuration of a monoterpene moiety, the size of the annulated aliphatic ring, and an increase in the length of an aliphatic bridge between the monoterpene and coumarin moieties from one to four CH_2_ groups were found to significantly affect the antiviral activity [10]. Based on the results of time-of-addition experiments and molecular modeling, the RSV F protein was suggested as a possible target.

These data and molecular modeling findings suggest that the introduction of an aromatic substituent in position 4 of the coumarin molecule may be very promising. In this study, we synthesized a library of compounds that included both previously obtained coumarin–monoterpenoid hybrids [11] and new compounds in which coumarin and monoterpene bicyclic fragments are separated from each other by three or four CH_2_ groups, as well as a number of nitrogen-containing coumarin derivatives with an NH_2_ group instead of an OH group.

## 2. Results and Discussion

### 2.1. Chemistry

**Scheme 1:**
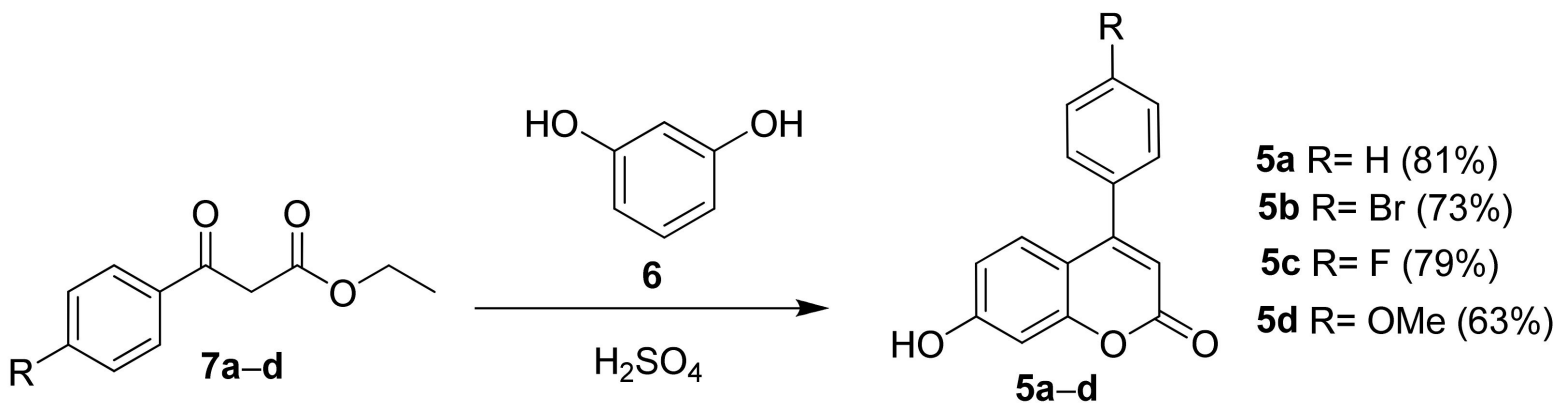
Synthesis of 7-hydroxy-4-arylcoumarins **5a**–**d**.

4-Arylcoumarins **5a**–**d** were synthesized according to [11] (Scheme 1) with yields 63–81%. Acid-catalyzed Pechmann condensation between resorcinol **6** and an ester of β-keto-carboxylic acids was used. Ester **7a** is commercially available, while compounds **7b**–**d** were obtained by reaction of substituted acetophenones **8b**–**d** with diethyl carbonate in the presence of sodium hydride.

**Scheme 2:**
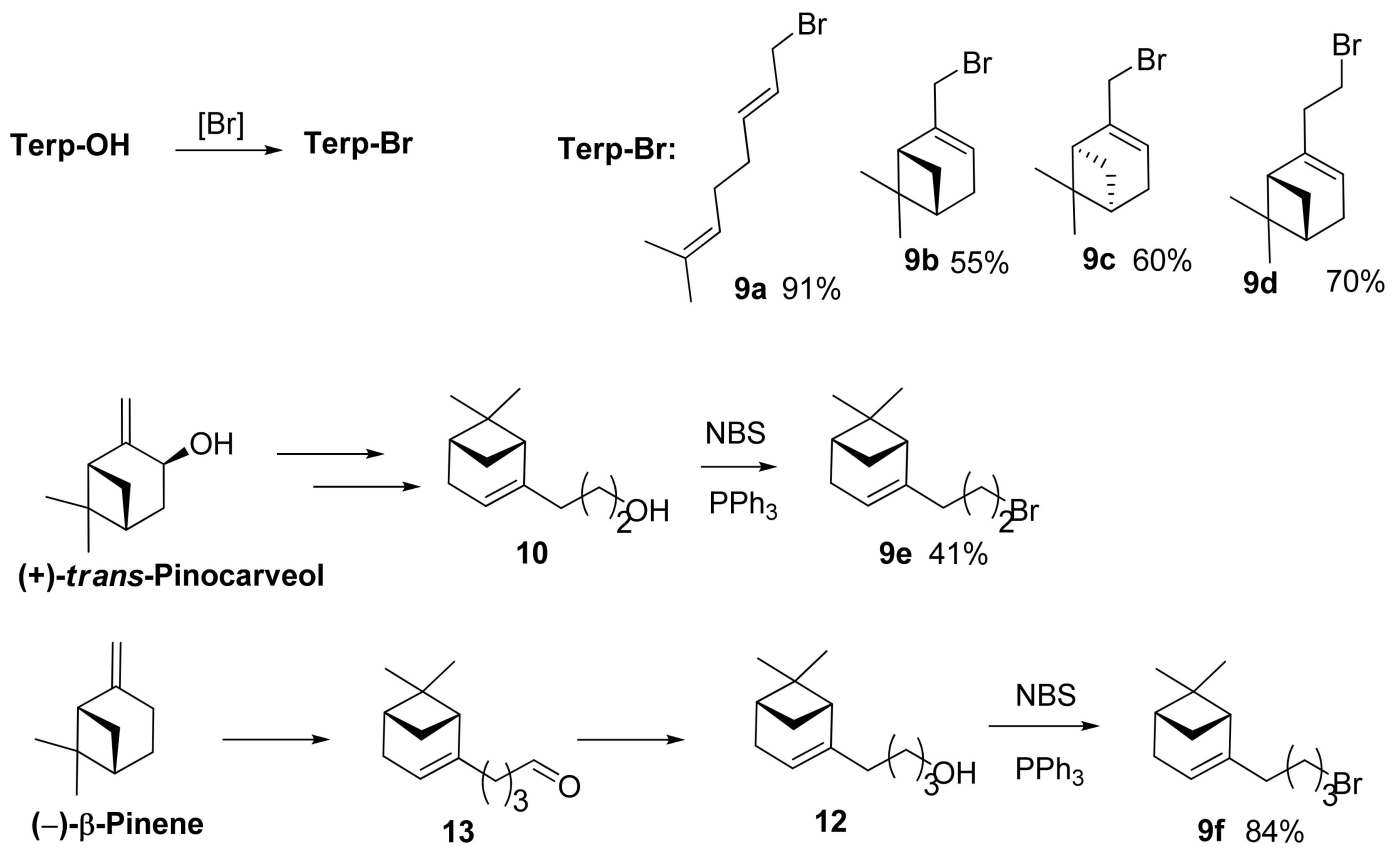
Synthesis of monoterpenoid bromides **9a**–**f**.

Reaction of geraniol, (–)-myrtenol, and (+)-myrtenol obtained from (+)-α-pinene [12] with PBr_3_ in accordance with [13] gave corresponding bromides **9a**–**c** (Scheme 2). Nopol bromide **9d** and its homologs **9e** and **9f** comprising additional CH_2_ groups were synthesized from the corresponding alcohols using the NBS–PPh_3_ system [14] (Scheme 2).

Nopol homolog **10** containing an additional CH_2_ group was synthesized from (+)-*trans*-pinocarveol prepared according to the procedure of [15] by reaction with triethyl orthoacetate in the presence of hexanoic acid to give ester **11** that was then reduced by LiAlH_4_ [16] (Scheme 2). Nopol homolog **12** containing two additional CH_2_ groups was synthesized by reaction of (−)-β-pinene with acrolein in the presence of ZnBr_2_ to form aldehyde **13** that was reduced by NaBH_4_ [17].

**Scheme 3:**
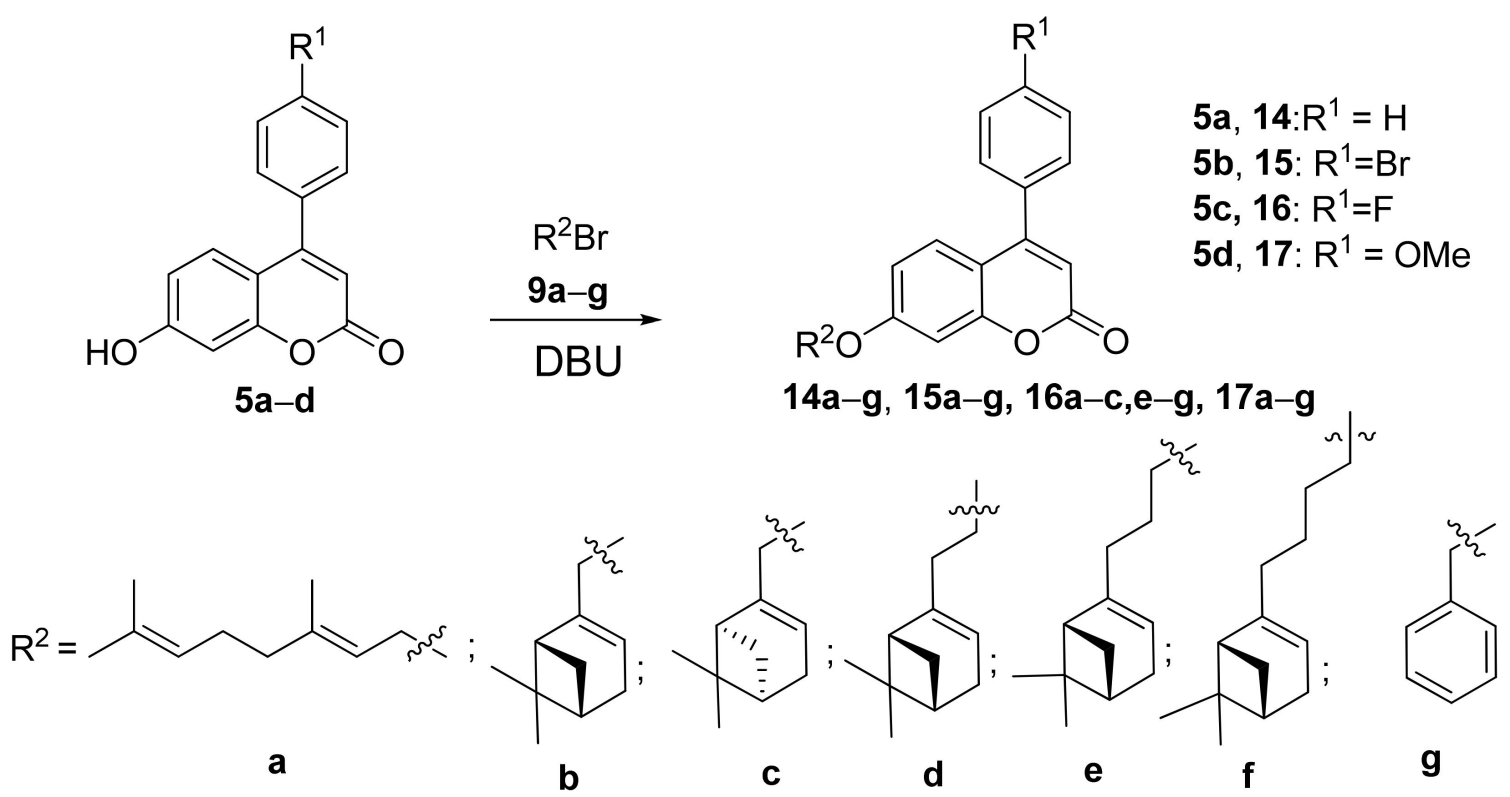
Synthesis of substituted coumarins **14**–**17**.

Coumarin derivatives **14**–**17** were obtained by reaction of 7-hydroxyarylcoumarins **5a**–**d** with the corresponding monoterpenoid bromides **9a**–**f** and benzylbromide **9g** using DBU and DMF as described previously [11] (Scheme 3). The products were purified by recrystallization or column chromatography (yields 38–72%).

**Scheme 4:**
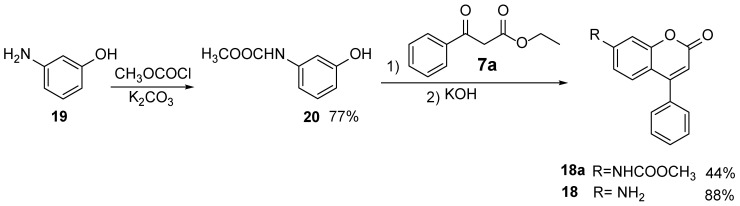
Synthesis of aminocoumarin **18**.

7-Arylcoumarin **18** was synthesized according to the procedure [18], starting from 3-aminophenol **19**, through intermediate compound **20** that reacted with ketoester **7a** (Scheme 4).

**Scheme 5:**
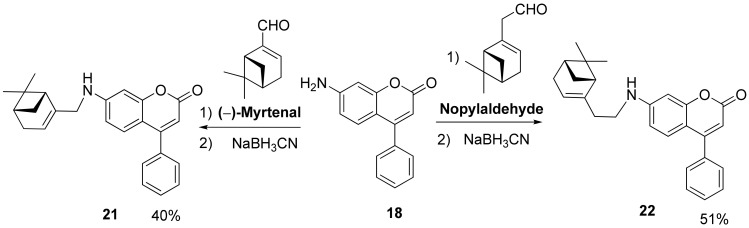
Synthesis of aminocoumarin derivatives **21** and **22**.

Next, secondary amine **21** was produced in the reaction between compound **18** and (–)-myrtenal, followed by reduction with NaBH_3_CN (Scheme 5). Similarly, amine **22** was synthesized from compound **18** and (–)-nopinal. Amine **21** was produced as an individual compound by recrystallization, while amine **22** was purified by column chromatography.

### 2.2. Biology

Cytotoxicity of compounds on non-infected HEp-2 cells was evaluated using the MTT assay to calculate CC_50_ values.

All compounds were evaluated in vitro for their antiviral activity against the RSV A strain A2 in HEp-2 cells. EC_50_ and SI values of each compound were taken as criteria for evaluating the antiviral activity.

The obtained results are shown in Table 1. Compounds were considered to be promising with a selectivity index of 10 or higher (highlighted by bold).

All monoterpene–coumarin conjugates were tested for their ability to inhibit replication of RSV A. Compound **14a**, comprising a geraniol moiety, exhibited moderate antiviral activity, but, given low cytotoxicity, a selectivity index of more than 10 was achieved. The replacement of a monoterpene moiety from geraniol to a (–)-myrtenol residue when going to compound **14b** led to a four-fold increase in activity and a three-fold increase in the selectivity index. However, compound **14c** produced using (+)-myrtenol was not active. An increase in the hydrocarbon chain length to two or four methylene groups, when going from **14a** to **14d** and **14f**, respectively, led to a decrease in the antiviral activity. Furthermore, compound **14e**, which differs from **14a** by the length of a 3-methylene group linker, demonstrated the highest activity among these compounds, inhibiting virus replication at low micromolar concentrations, which, together with low cytotoxicity, led to a high selectivity index of 193. The replacement of a monoterpene moiety by a benzyl fragment (compound **14g**) completely eliminated the antiviral effect.

The introduction of a bromine atom into the *para*-position of an aromatic moiety significantly increased cytotoxicity of coumarins **15** and, accordingly, lowered the selectivity index. A selectivity index of about 10 was found only for compound **15f** that had the longest aliphatic linker between the pinene and coumarin moieties among the tested compounds.

Cytotoxicity of compounds similar to **16**, which have a fluorine atom in the aryl substituent, was in between that of compounds **14** and **15**. Significant selectivity indices of 9 and 11 were found for compounds **16e** and **16f**, respectively, which differed by one methylene group in the linker, with the latter compound being ten-fold more active (EC_50_ of 9 µM).

Among methoxy derivatives **17**, compound **17f** was the most active (EC_50_, 10 µM; selectivity index, 11). Compound **17d** had the same selectivity index, but was six-fold less active than **17f**.

The replacement of oxygen by the NH group when going from **14b** to **21** led to an abrupt increase in cytotoxicity and, therefore, to a low selectivity index; in this case, compound **22** exhibited activity and toxicity comparable to those of oxygen-containing analog **14d**.

Compound **14e**, which exhibited both the best antiviral activity and the highest selectivity index, was tested for its ability to inhibit reproduction of RSV type B. Compound **14e** was found to be efficient against both types of the virus, with EC_50_ for RSV B being 3 ± 0.2 µM.

**Figure 2:**
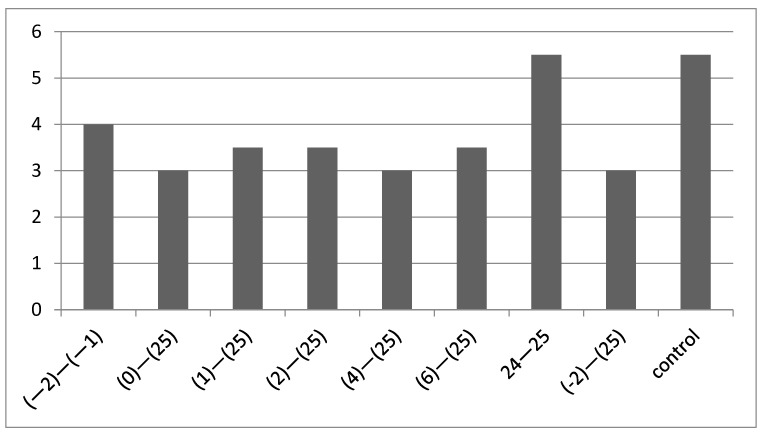
Dependence of the intensity of virus growth on the time of **14e** addition.

**Figure 3:**
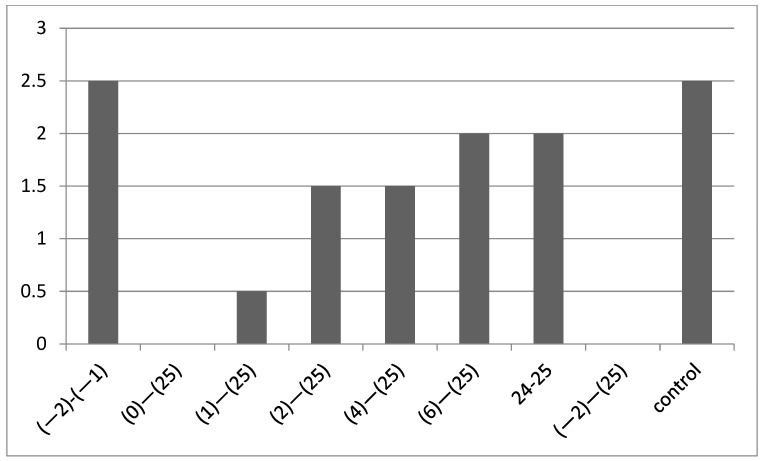
Dependence of the intensity of virus growth on the time of **14b** addition.

To clarify action mechanisms of the most active compounds, an experiment using the time-of-addition method was carried out. Compounds **14e** and **14b** at a concentration of 300 μg/mL were added at different time intervals before, after, or during virus inoculation (Figure 2 and Figure 3). The time of compound addition was counted off from Point 0, i.e., the moment of virus entry into the cell. RSV A2 virus was added to cells at a time conventionally designated as Point 1; then, cells were kept at a temperature of 40 °C for 1 h. Next, at Point 0, unbound virus was washed off, fresh medium containing compounds at the same concentration was added, and the cells were transferred to a thermostat at 37 °C where they were incubated for 25 h. After this, medium collected from each well was used to make a series of ten-fold dilutions with fresh cell culture. The cells were incubated for 6 days. The viral titer was measured by ELISA.

Compound **14e** reduced the viral titer upon addition at all time periods through 6 h after infection, and compound **14b** reduced the viral titer at all time periods, except for prophylactic use 2 h before virus entry into cells. A decrease by 0.5 lgTID_50_ was observed upon compound addition at 6 and 24 h after infection. The maximum titer decrease (up to complete virus elimination) was observed at Point 0; then, the later the compound was added, the higher the viral titer was. Putative targets for compounds of this group are surface proteins F and/or G, as well as the L protein (polymerase).

### 2.3. Molecular Modeling Study

According to the results of the time-of-addition experiment and the earlier findings for coumarin **3** [10], the surface F protein can be considered to be a potential biological target.

#### 2.3.1. The Pharmacophoric Profile of the Binding Site to F Protein Inhibitor

**Figure 4:**
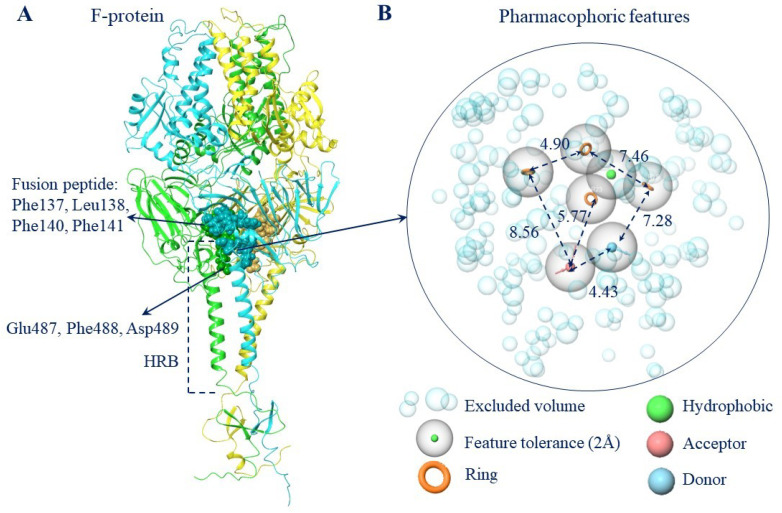
(**A**) Position of the a.a. binding site within the RSV F protein trimer. The number of functional a.a. fusion peptide (a.a. 137–155) and heptadic repeat (HBR, a.a. 476–524) are shown; (**B**) the pharmacophoric profile of the binding site. The distance between the fragments is shown in Å.

The binding site to the known F protein inhibitors or the DS-Cav1 cavity [19] resides in the central part of the trimer. The key amino acids (a.a.) are Phe137, Leu138, Phe140, and Leu141 of fusion peptide (FP) and Glu487, Phe488, and Asp489 of the heptad repeat (Figure 4A). The FP is located at the terminus of the F1 subunit, and the α-helix of HR resides at the C-terminus of the F2 subunit. Protein parts both undergo abrupt conformational rearrangements during the fusion. According to [20], binding of inhibitors within a binding site affects the conformation of the side chains of these amino acids and affects the thermodynamic component of the transition from the pre- to the post-fusion conformation.

The pharmacophoric profile of the binding site was constructed based on the position of the key amino acids and contains a number of significant pharmacophoric features (Figure 4B). According to the pharmacophoric profile, the “ideal” ligand should contain more than one aromatic fragment, a donor and an acceptor group, and at least one hydrophobic fragment.

The physical properties and matches of the ligand fragments to the pharmacophore features of the binding site obviously depend on their structure. All the phenyl coumarins studied share a common scaffold, phenyl-chromen-2-one. The structural descriptors and matches of the ligand fragments to the pharmacophoric profile of the binding site obviously depend on substituents. In general, longer linker length increases the lipophilicity value (Tables S1–S3). Meanwhile, the sum of the surface of all polar atoms (PSA) does not exceed 49 Å, thus inferring that all the studied ligands must have good membrane permeability [21].

Matches of the fragments to pharmacophoric signs of the binding site is observed for most studied ligands. The presence of aromatic rings is largely important. The presence of essential structural descriptors in the ligand does not mean that would reside in the “right” place of the site. The spatial position of the linker is also an important parameter.

For arylcoumarins (Table S1) containing the hydrophobic pinene (dimethylbicyclo[3.1.1]heptene) fragment, three or four (for **21**) matches are observed in the compounds with the smallest linker, one of which is an acceptor or donor (**21**), to a hydrogen bond. For a linker with length *n* = 2 or 3, three are a hydrophobic fragment and aromatic rings; for *n* = 4, there are less than three of them. Meanwhile, the more matches to the pharmacophore profile of the site the ligands have, the more fully the ligand will occupy the entire space of the linking site.

Compounds with diene substituents (Table S2) have less than three matches, and compounds containing a benzyl substituent instead of a monoterpene carry only three aromatic rings. Meanwhile, less than three matches are observed for the **14g** compound.

In general, ligands characterized by high pIC_50_ have three or more matches with the pharmacophore profile of the site.

The inhibitory activity of the compounds also depends on the stereoisomerism of the hydrophobic pinene fragment. Thus, (*S*)-stereoisomers containing the (−)-pinene fragment are characterized by more than three matches: hydrogen bond acceptor and aromatic rings (Table S3), while the (*R*)-isomers have less than three matches. In this case, an analysis of pIC_50_ shows that the *S* isomers are generally more active compared to the *R* ones.

#### 2.3.2. The Molecular Docking Data

**Figure 5:**
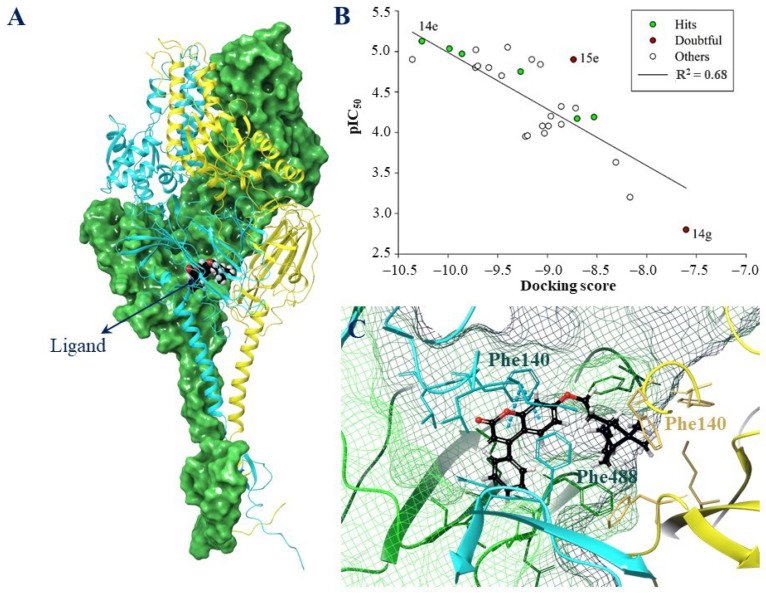
(**A**) The ligand binding site in the trimer; (**B**) the correlation between the experimental and theoretical data; (**C**) position of the leader **14e** connection at the binding site π–π interaction stacking is shown with blue discontinuous lines.

Twenty docking solutions were set as the maximum possible number under the induced docking protocol. For a number of ligands, less than 20 docking positions were implemented, although this result obviously depends on ligand mobility (Table S4). The binding site of F-protein inhibitors is saturated with aromatic and hydrophobic amino acid residues. In fact, when bound in the tested site, all arylcoumarins are located in a hydrophobic cavity in the central part of the trimer (Figure 5A), mainly giving rise to the exactly π–π stacking interaction between the aromatic rings of ligands and the Phe fusion loop (Phe140, Phe137) and the heptadic repeat (Phe488) (Table S4). Hydrophobic contacts are also detected between the a.a. of the binding site and the hydrophobic fragments of the studied compounds. 

The optimal ligand position was chosen to find correlations between the inhibitory activity values in vitro and the docking score. On the one hand, the molecular docking data should be considered to be some kind of “rough” approach to answer the question whether the ligand can bind at the binding site under study. The correlation between the experimental and theoretical data may be not obvious. On the other hand, however, an analysis of the matches of structural ligand descriptors to the pharmacophore profile of the binding site, together with time-of-addition experiments, leave no doubt that the chosen biological target and the binding site were correct.

In any case, there is a correlation between the results of molecular docking of arylcoumarines and the data of biological experiments (Figure 5B). However, a number of compounds obviously are “given out” (e.g., **14g** and **15e**). 

In the binding site, the compound leader **14e** is associated primarily with formation of π–π staking interactions with Phe and hydrophobic contacts with surrounding amino acids (Figure 5C). A docking position corresponding to the optimal energy and structural parameters was used to prepare a starting position for molecular dynamic simulations.

#### 2.3.3. The Results of Molecular Dynamics Simulations

The main objective of the molecular dynamics simulations was to evaluate the behavior of the leader compound within the binding site. The model system was constructed in such a way that the transmembrane domain of the protein was immersed in the membrane [8].

**Figure 6:**
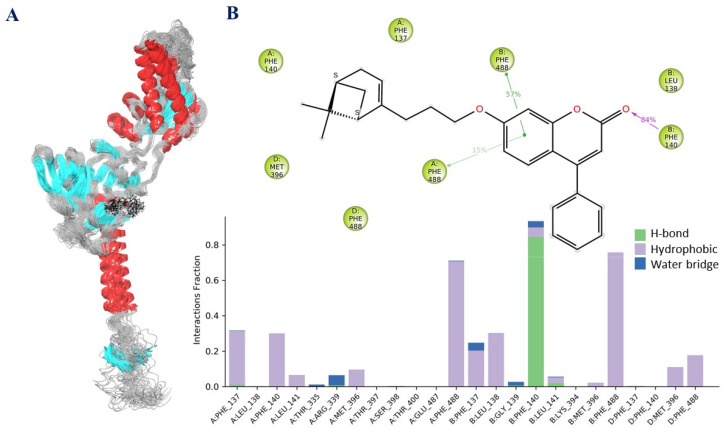
The results of molecular dynamics simulations: (**A**) Superimposition of geometric parameters of the ligand–protein complex (only the F protein protomer is shown to facilitate visualization) corresponding to 100 frames with a step of 100 (the ligand molecules are shown in green); (**B**) the frequency (in %) of contacts between the a.a. binding site and the ligand is shown (pi–pi stacking interactions are shown with green lines, hydrogen bonds are shown with a purple arrow) and the histogram of the interaction between the ligand and the a.a. of the binding sites, along the Y axis the time of contact (in fractions) of the ligand with the a.a. is delayed.

According to the analysis of the RMSD plot, the ligand–protein system equalized to 80 ns of molecular dynamics simulations (Figure S1). During the entire simulation, the ligands were located inside a symmetrical binding site and did not diffuse into a solvent. However, the geometric parameters of the ligand at the binding site changed (Figure 6A). The ligand was mobile; the structure unfolds during the simulations.

Located in the binding site, the ligand formed mainly hydrophobic interactions with a symmetrical set of a.a. of the binding site, namely with the a.a. of fusion peptide 137–141 and heptadic repeat 335, 396–400, 487, and 488 (Figure 6B). For over 80% of the simulation time, the hydrogen bridge between carbonyl oxygen and Phe140 was preserved (more than 50% of the hydrophobic contact with Phe488). 

Additionally, short-lived contacts mediated by water were detected. In general, during the entire time of molecular dynamics simulations, the ligand formed at least eight intermolecular contacts with the a.a. of the binding site (Figure S2).

Compound leader **14e** was characterized by three out of the seven possible fragments corresponding to the pharmacophoric profile of the site, low values of binding energy parameters and a set π–π stacking of interactions with functional a.a. An analysis of molecular dynamics simulations showed that the ligand was quite freely located in the site. Only two contacts persisted for more than 50% of the simulation time. The totality of the molecular modeling data allows us to make assumptions that structural modification of the compound is needed, at least inserting one hydrogen bond donor fragment (e.g., a formalin fragment or an amino group). The similar fragments in the known F protein inhibitors TMC-353121 and JNJ-240868 cause formation of an additional salt bridge between the positively charged ligand fragments and the negatively charged amino acids Asp486 and Glu487 [20].

## 3. Materials and Methods

### 3.1. Chemistry

General Information: Reagents and solvents were purchased from commercial suppliers (Sigma-Aldrich (St. Louis, MO, USA), Acros (Waltham, MA, USA)) and used as received. GC-MS: *Agilent 7890A* gas chromatograph (Santa Clara, CA, USA) equipped with a quadrupole mass spectrometer *Agilent 5975C* as a detector; quartz column HP-5MS (copolymer 5% diphenyl and 95% dimethylsiloxane) of length 30 m, internal diameter 0.25 mm, and stationary phase film thickness 0.25 µm. Optical rotation: polAAr 3005 spectrometer. ^1^H and ^13^C NMR: *Bruker DRX-500* apparatus at 500.13 MHz (^1^H) and 125.76 MHz (^13^C) and *Bruker Avance–III 600* apparatus (Billerica, MA, USA) at 600.30 MHz (^1^H) and 150.95 MHz (^13^C), *J* in Hz; structure determinations by analyzing the ^1^H NMR spectra, including ^1^H–^1^H double resonance spectra and ^1^H–^1^H 2D homonuclear correlation, *J*-modulated ^13^C NMR spectra (JMOD), and ^13^C–^1^H 2D heteronuclear correlation with one-bond and long-range spin-spin coupling constants (C–H COSY, ^1^*J*(C,H) = 160 Hz, COLOC, ^2,3^*J*(C,H) = 10 Hz). HR-MS: DFS Thermo Scientific spectrometer (Waltham, MA, USA) in a full scan mode (15–500 *m*/*z*, 70 eV electron impact ionization, direct sample administration). 

Spectral and analytical investigations were carried out at the Multi-Access Chemical Research Center of the Siberian Branch of Russian Academy of Sciences. All product yields were given for pure compounds purified by recrystallization from ethanol or isolated by column chromatography (SiO_2_; 60–200 μ; *Macherey-Nagel* (Dueren, Germany)). The purity of the target compounds was determined by GC-MS methods. All of the target compounds reported in this paper had a purity of no less than 95%.

#### 3.1.1. Synthesis of Coumarins **5a**–**d**

To a stirred mixture of sodium hydride (3 mol equiv) washed with hexane (3 × 15 mL) and diethyl carbonate (4 mol equiv) in 50 mL of tetrahydrofuran (THF) was added dropwise appropriately substituted acetophenone (1 mol equiv) over 30 min. Reaction mixture was refluxed for 4 h. The reaction mixture was poured into ice water, acidified with 5 mL glacial acetic acid, and extracted with EtOAc (3 × 100 mL). Combined organic phase was washed with saturated sodium bicarbonate, brine and water, dried over anhydrous Na_2_SO_4_, and evaporated in vacuo; the crude products were purified by silica gel column chromatography eluting with dichloromethane to afford **7b**–**d**. The yields of **7b**–**d** were 80%, 89%, and 92% respectively.

Syntheses were carried out from resorcinol **6** and appropriate β-keto esters (**7a**–**d**) in accordance with [11]. Yields of **5a**–**d** were 81%, 73%, 79%, and 63% respectively.

#### 3.1.2. Synthesis of Bromides **9a**–**c**

Bromides **9a**–**c** were synthesized from geraniol(–), (−)-myrtenol, and (+)-myrtenol via the reaction with PBr_3_ with the yields 91%, 55%, and 60%, respectively [10].

#### 3.1.3. Synthesis of Bromides **9d**–**f**

Bromides **9d**–**f** were synthesized from (−)-nopol and via reaction with NBS –PPh_3_ as described in [10]. Yields of **9d**–**f** were 70%, 41%, and 84% respectively.

#### 3.1.4. Synthesis of Compounds **14a**–**g**, **15a**–**g**, **16a**–**c**, **16e**–**g**, and **17a**–**g**

Compounds **14a**–**g**, **15a**–**g**, **16a**–**c**, **16e**–**g, 17a**–**d**, and **17f,g** were synthesized from coumarins **5a**–**d** and corresponding bromides **9a**–**g** with the use DBU and DMF [11].

General procedure: DBU (1.0 mmol) and corresponding bromide **9a**–**g** (0.75 mmol) were added to compound **5a**–**d** (0.5 mmol) in dry DMF (5 mL) at r.t. under stirring. The reaction mixture was stirred at r.t. for 15 min, and then heated at 60 °C for 5 h. H_2_O (15 mL) was added and the product was extracted with ethyl acetate. The extracts were washed with brine, dried with Na_2_SO_4_, and evaporated. The products **14a**–**g**, **15a**–**g**, **16a**–**c**, **16e**–**g,** and **17a**–**g** were isolated in the individual form (Method a) by recrystallization from ethanol or (Method b) by column chromatography on silica gel, eluent—hexane, solution containing from 25 to 100% ethyl acetate in hexane, ethanol.

^1^H and ^13^C NMR data for compounds **14a**–**d**, **14g, 15a**–**d**, **15g, 16a**–**c, 17a**–**d**, and **17g** correspond to those published earlier [11].

*7-(3-((1R,5S)-6,6-Dimethylbicyclo[3.1.1]hept-2-en-2-yl)propoxy)-4-phenyl-2H-chromen-2-one* **14e** Yield 56%, Method b. ${\left[\alpha \right]}_{589}^{24.5}$ = −14.9 (*c* = 0.83, CHCl_3_). HRMS: 399.1959 [M − H]^+^; calcd. 399.1955 (C_27_H_27_O_3_). ^1^H-NMR (CDCl_3_, *δ* ppm, J, Hz): 0.82 (s, 3H-C(27)); 1.13 (d, 1H, ^2^*J* = 8.5, H-C(25a)); 1.25 (s, 3H-C(26)); 1.79–1.91 (m, 2H-C(17)); 2.02 (ddd, 1H, *J*(24,22) = *J*(24,25s)= 5.6, *J*(24,20) = 1.4, H-C(24)); 2.05–2.13 (m, 3H, 2H-C(18), H-C(22)); 2.17 (dm, 1H, ^2^*J* = 17.5, H-C(21)) 2.23 (dm, 1H, ^2^*J* = 17.5, H’-C(21)); 2.35 (ddd. 1H, ^2^*J* = 8.5, *J*(25s,22) = *J*(25s,24) = 5.6, H-C(25s)); 3.99 (t, 2H, *J*(16,17) = 6.5, 2H-C(16)); 5.20–5.23 (m, H-C(20)); 6.18 (s, H-C(3)); 6.76 (dd, 1H, *J*(7,6) = 8.9, *J*(7,9) = 2.5, H-C(7)); 6.85 (d, 1H, *J*(9,7) = 2.5, H-C(9)); 7.35 (d, 1H, *J*(6,7) = 8.9, H-C(6)); 7.39–7.43 (m, 2H, H-(C-11), H-C(15)); 7.47–7.51 (m, 3H, H-C(12), H-C(13), H-C(14)). ^13^C-NMR (CDCl_3_, δ_C_): 155.88 (s, C(1)); 161.16 (s, C(2)); 111.56 (d, C(3)); 155.72 (s, C(4)); 112.20 (s, C(5)); 127.77 (d, C(6)); 112.59 (d, C(7)); 162.23 (s, C(8)); 101.41 (d, C(9)); 135.49 (s, C(10)); 128.24 (d, C(11)); 128.67 (d, C(12); 129.41 (d, C(13)); 128.67 (d, C(14); 128.24 (d, C(15)); 68.15 (t, C(16)); 26.44 (t, C(17)); 32.87 (t, C(18)); 146.93 (s, C(19)); 116.67 (d, C(20)); 31.13 (t, C(21)); 40.68 (d, C(22)); 37.82 (s, C(23)); 45.58 (d, C(24)); 31.57 (t, C(25)); 26.17 (q, C(26)); 21.06 (q, C(27)).
*7-(4-((1R,5S)-6,6-Dimethylbicyclo[3.1.1]hept-2-en-2-yl)butoxy)-4-phenyl-2H-chromen-2-one* **14f** Yield 40%, Method a. M.p. 40 °C. ${\left[\alpha \right]}_{589}^{26}$ = −12.8 (*c* = 1.85, CHCl_3_) HRMS: 413.2114 [M − H]^+^; calcd. 413.2111 (C_28_H_29_O_3_). ^1^H-NMR (CDCl_3_, *δ* ppm, *J*, Hz): 0.82 (s, 3H-C(28)); 1.12 (d, 1H, ^2^*J* = 8.5, H-C(26a)); 1.25 (s, 3H-C(27)); 1.44–1.58 (m, 2H-C(18)); 1.75–1.83 (m, 2H-C(17)); 1.96–2.02 (m, 3H, 2H-C(19), H-C(25)); 2.04–2.09 (m, H-C(23)); 2.17 (dm, 1H, ^2^*J* = 17.5, H-C(22)); 2.23 (dm, 1H, ^2^*J* = 17.5, H’-C(22)); 2.34 (ddd, 1H, ^2^*J* = 8.5, *J*(26s,23) = *J*(26s,25) = 5.6, H-C(26s)); 4.01 (t, 2H, *J*(16,17) = 6.5, 2H-C(16)); 5.17–5.21 (m, H-C(21)); 6.18 (s, H-C(3)); 6.76 (dd, 1H, *J*(7,6) = 8.9, *J*(7,9) = 2.5, H-C(7)); 6.85 (d, *J*(9,7) = 2.5, H-C(9)); 7.34 (d, 1H, *J*(6,7) = 8.9, H-C(6)); 7.39–7.43 (m, 2H, H-(C-11), H-C(15)); 7.46–7.51 (m, 3H, H-C(12), H-C(13), H-C(14)). ^13^C-NMR (CDCl_3_, δ_C_): 155.90 (s, C(1)); 161.13 (s, C(2)); 111.58 (d, C(3)); 155.70 (s, C(4)); 112.21 (s, C(5)); 127.76 (d, C(6)); 112.58 (d, C(7)); 162.25 (s, C(8)); 101.47 (d, C(9)); 135.52 (c, C(10)); 128.24 (d, C(11)); 128.67 (d, C(12); 129.40 (d, C(13)); 128.67 (d, C(14); 128.24 (d, C(15)); 68.42 (t, C(16)); 28.59 (t, C(17)); 23.35 (t, C(18)); 36.34 (t, C(19)); 147.71 (c, C(20)); 116.16 (d, C(21)); 31.14 (t, C(22)); 40.76 (d, C(23)); 37.81 (c, C(24)); 45.63 (d, C(25)); 31.55 (t, C(26)); 26.22 (q, C(27)); 21.07 (q, C(28)).
*4-(4-Bromophenyl)-7-(3-((1R,5S)-6,6-dimethylbicyclo[3.1.1]hept-2-en-2-yl)propoxy)-2H-chromen-2-one* **15e** Yield 68%, Method b. ${\left[\alpha \right]}_{589}^{24.5}$ = −13.1 (*c* = 0.55, CHCl_3_). HRMS: 477.1066 [M − H]^+^; calcd. 477.1060 (C_27_H_26_O_3_^79^Br_1_). ^1^H-NMR (CDCl_3_, *δ* ppm, *J*, Hz): 0.82 (s, 3H-C(27)); 1.13 (d, 1H, ^2^*J* = 8.5, H-C(25a)); 1.26 (s, 3H-C(26)); 1.79–1.91 (2H-C(17)); 2.02 (ddd, 1H, *J*(24,22) = *J*(24,25s)= 5.6, *J*(24,20) = 1.4, H-C(24)); 2.05–2.13 (m, 2H-C(18), H-C(22)); 2.17 (dm, 1H, ^2^*J* = 17.5, H-C(21)); 2.23 (dm, 1H, *J* = 17.5, H’-C(17)); 2.35 (ddd, 1H, ^2^*J* = 8.5, *J*(25s,22) = *J*(25s,24) = 5.6, H-C(25s)); 3.99 (t, 2H, *J*(16,17) = 6.5, 2H-C(16)); 5.20–5.23 (m, 1H, H-C(20)); 6.16 (s, H-C(3)); 6.77 (dd, 1H, *J*(7,6) = 8.9, *J*(7,9) = 2.5, H-C(7)); 6.85 (d, *J*(9,7) = 2.5, H-C(9)); 7.27–7.31 (m, 3H, H-C(6), H-(C-11), H-C(15)); 7.63 (d, 2H, *J*(12,11) = *J*(14,15) = 8.5, H-C(12), H-C(14)). ^13^C-NMR (CDCl_3_, δ_C_): 155.92 (s, C(1)); 160.88 (s, C(2)); 111.67 (d, C(3)); 154.51 (s, C(4)); 111.84 (s, C(5)); 127.45 (d, C(6)); 112.78 (d, C(7)); 162.44 (s, C(8)); 101.55 (d, C(9)); 134.36 (c, C(10)); 129.85 (d, C(11)); 132.00 (d, C(12); 123.88 (c, C(13)); 132.00 (d, C(14); 129.85 (d, C(15)); 68.23 (t, C(16)); 26.45 (t, C(17)); 32.88 (t, C(18)); 146.93 (c, C(19)); 116.72 (d, C(20)); 31.15 (t, C(21)); 40.72 (d, C(22)); 37.85 (c, C(23)); 45.63 (d, C(24)); 31.59 (t, C(25)); 26.20 (q, C(26)); 21.08 (q, C(27)).
*4-(4-Bromophenyl)-7-(4-((1R,5S)-6,6-dimethylbicyclo[3.1.1]hept-2-en-2-yl)butoxy)-2H-chromen-2*-one **15f** Yield 38%, Method a. M.p. 44 °C. ${\left[\alpha \right]}_{589}^{22}$ = −8.2 (*c* = 0.95, CHCl_3_). HRMS: 491.1214 [M − H]^+^; calcd. 491.1216 (C_28_H_29_O_3_^79^Br_1_). ^1^H-NMR (CDCl_3_, *δ* ppm, *J*, Hz): 0.81 (s, 3H-C(28)); 1.12 (d, 1H, ^2^*J* = 8.5, H-C(26a)); 1.25 (s, 3H-C(27)); 1.43–1.57 (m, 2H-C(18)); 1.75–1.83 (m, 2H-C(17)); 1.96–2.02 (m, 3H, 2H-C(19), H-C(25)); 2.03–2.09 (m, H-C(23)); 2.16 (dm, ^2^*J* = 17.5, H-C(22)); 2.23 (dm, 1H, ^2^*J* = 17.5, H’-C(22)); 2.34 (ddd, 1H, ^2^*J* = 8.5, *J*(26s,23) = *J*(26s,25) = 5.6, H-C(26s)); 4.00 (t, 2H, *J*(16,17) = 6.5, 2H-C(16)); 5.17–5.21 (m, 1H, H-C(21)); 6.15 (s, 1H, H-C(3)); 6.76 (dd, 1H, *J*(7,6) = 8.9, *J*(7,9) = 2.5, H-C(7)); 6.85 (d, 1H, *J* = 2.5, H-C(9)); 7.26–7.31 (m, 3H, H-C(6), H-(C-11), H-C(15)); 7.63 (m, 2H, H-C(12), H-C(14)). ^13^C-NMR (CDCl_3_, δ_C_): 155.92 (s, C(1)); 160.85 (s, C(2)); 111.67 (d, C(3)); 154.48 (s, C(4)); 111.81 (s, C(5)); 127.43 (d, C(6)); 112.75 (d, C(7)); 162.44 (s, C(8)); 101.59 (d, C(9)); 134.37 (c, C(10)); 129.84 (d, C(11)); 131.99 (d, C(12); 123.87 (c, C(13)); 131.99 (d, C(14); 129.84 (d, C(15)); 68.49 (t, C(16)); 28.58 (t, C(17)); 23.35 (t, C(18)); 36.34 (t, C(19)); 147.70 (c, C(20)); 116.19 (d, C(21)); 31.15 (t, C(22)); 40.76 (d, C(23)); 37.82 (c, C(24)); 45.64 (d, C(25)); 31.56 (t, C(26)); 26.23 (q, C(27)); 21.08 (q, C(28)).
*7-(3-((1R,5S)-6,6-Dimethylbicyclo[3.1.1]hept-2-en-2-yl)propoxy)-4-(4-fluorophenyl)-2H-chromen-2-one* **16e** Yield 72%, Method b. ${\left[\alpha \right]}_{589}^{24.5}$ = −16.6 (*c* = 0.70, CHCl_3_). HRMS: 417.1856 [M − H]^+^; calcd. 417.1861 (C_27_H_26_O_3_^79^F_1_).∙^1^H-NMR (CDCl_3_, *δ* ppm, *J*, Hz): 0.81 (s, 3H-C(27)); 1.13 (d, 1H, ^2^*J* = 8.5, H-C(25a)); 1.25 (s, 3H-C(26)); 1.79–1.91 (m, 2H-C(17)); 2.02 (ddd, *J*(24,22) = *J*(24,25s) = 5.6, *J*(24,20) = 1.3, H-C(24)); 2.05–2.13 (m, 2H-C(18), H-C(22)); 2.17 (dm, ^2^*J* = 17.5, H-C(21)); 2.23 (dm, 1H, ^2^*J* = 17.5, H’-C(21)); 2.35 (ddd, 1H, ^2^*J* = 8.5, *J*(25s,22) = *J*(25s,24) = 5.6, H-C(25s)); 3.99 (t, 2H, *J* = 6.5, *J*(16,17) = 6.5, 2H-C(16)); 5.20–5.23 (m, H-C(20)); 6.16 (s, H-C(3)); 6.77 (dd, *J* = 8.8, 2.5, H-C(7)); 6.85 (d, *J* = 2.5, H-C(9)); 7.16–7.21 (m, 2H, H-(C-12), H-C(14)); 7.31 (d, *J* = 8.8, H-C(6)); 7.38–7.43 (m, 2H, H-C(11), H-C(15)). ^13^C-NMR (CDCl_3_, δ_C_): 155.88 (s, C(1)); 160.98 (s, C(2)); 111.71 (d, C(3)); 154.65 (s, C(4)); 112.08 (s, C(5)); 127.52 (d, C(6)); 112.71 (d, C(7)); 162.35 (s, C(8)); 101.49 (d, C(9)); 131.48 (d, *J* = 3.4, C(10)); 130.18 (d, *J* = 8.3, C(11) and C(15)); 115.89 (d, *J* = 21.7, C(12) and C(14)); 163.32 (d, ^1^*J* = 250.1, C(13)); 68.19 (t, C(16)); 26.43 (t, C(17)); 32.86 (t, C(18)); 146.92 (c, C(19)); 116.70 (d, C(20)); 31.13 (t, C(21)); 40.68 (d, C(22)); 37.83 (c, C(23)); 45.59 (d, C(24)); 31.58 (t, C(25)); 26.18 (q, C(26)); 21.07 (q, C(27)).
*7-(4-((1R,5S)-6,6-Dimethylbicyclo[3.1.1]hept-2-en-2-yl)butoxy)-4-(4-fluorophenyl)-2H-chromen-2-one* **16f** Yield 55%, Method a. M.p. 40 °C. ${\left[\alpha \right]}_{589}^{26}$ = −8.0 (*c* = 1.28, CHCl_3_). HRMS: 431.2023 [M − H]^+^; calcd. 431.2017 (C_28_H_28_O_3_^79^F_1_).∙^1^H-NMR (CDCl_3_, *δ* ppm, *J*, Hz): 0.81 (s, 3H-C(28)); 1.12 (d, 1H, ^2^*J* = 8.5, H-C(26a)); 1.25 (s, 3H-C(27)); 1.43–1.57 (m, 2H-C(18)); 1.76–1.82 (m, 2H-C(17)); 1.96–2.02 (m, 3H, 2H-C(19), H-C(25)); 2.04–2.08 (m, H-C(23)); 2.17 (dm, 1H, ^2^*J* = 17.4, H-C(22)); 2.23 (dm, 1H, ^2^*J* = 17.4, H’-C(22)); 2.33 (ddd, 1H, ^2^*J* = 8.5, *J*(26s,23) = *J*(26s,25) = 5.6, H-C(26s)); 4.00 (t, 2H, *J*(16,17) = 6.5, 2H-C(16)); 5.17–5.20 (m, H-C(21)); 6.15 (s, H-C(3)); 6.77 (dd, 1H, *J*(7,6) = 8.9, *J*(7,9) = 2.5, H-C(7)); 6.85 (d, 1H, *J* = 2.5, H-C(9)); 7.16–7.21 (m, 2H, *J*(12,11)= *J*(14,15) = 8.7, *J*(12(14),F) = 9.0, H-C(12), H-C(14)); 7.30 (d, 1H, *J*(6,7) = 8.9, H-C(6)); 7.38–7.43 (m. 2H, *J*(11,12)= *J*(15,14) =8.7, *J*(11(15),F)= 5.2, H-(C-11), H-C(15)). ^13^C-NMR (CDCl_3_, δ_C_): 155.90 (s, C(1)); 161.00 (s, C(2)); 111.73 (d, C(3)); 154.65 (s, C(4)); 112.08 (s, C(5)); 127.52 (d, C(6)); 112.72 (d, C(7)); 162.36 (s, C(8)); 101.54 (d, C(9)); 131.50 (d, ^4^*J* = 3.5, C(10)); 130.21 (d, C(11)); 115.89 (d, ^2^*J* = 21.7, C(12) and C(14)); 163.34 (d, ^1^*J* = 250.1, C(13)); 130.16 (d, C(15)); 68.47 (t, C(16)); 28.58 (t, C(17)); 23.35 (t, C(18)); 36.35 (t, C(19)); 147.71 (c, C(20)); 116.19 (d, C(21)); 31.15 (t, C(22)); 40.75 (d, C(23)); 37.83 (c, C(24)); 45.62 (d, C(25)); 31.56 (t, C(26)); 26.22 (q, C(27)); 21.08 (q, C(28)).
*7-(Benzyloxy)-4-(4-fluorophenyl)-2H-chromen-2-one* **16g** Yield 50%, Method b. HRMS: 346.1006 [M]^+^; calcd. 346.1000 (C_22_H_15_O_3_F_1_). ^1^H-NMR (CDCl_3_, *δ* ppm, *J*, Hz): 5.12 (s, 2H, 2H-C(16)); 6.17 (s, 1H, H-C(3)); 6.86 (dd, 1H, *J*(7,6) = 8.9, *J*(7,9) = 2.5, H-C(7)); 6.94 (d, 1H, *J*(9,7) = 2.5, H-C(9)); 7.16–7.21 (m, 2H, *J*(12,11) = *J*(14,15) = 8.6, *J*(12(14),F) = 8.6, H-C(12), H-C(14)); 7.32 (d, 1H, *J*(6,7) = 8.9, H-C(6)); 7.30–7.35 (m, 1H, H-C(20)); 7.35–7.44 (m, 6H, H-C(11), H-C(15), H-C(18), H-C(19), H-C(21), H-C(22)). ^13^C-NMR (CDCl_3_, δ_C_): 155.81 (s, C(1)); 160.83 (s, C(2)); 112.02 (d, C(3)); 154.56 (s, C(4)); 112.50 (s, C(5)); 127.64 (d, C(6)); 112.93 (d, C(7)); 161.83 (s, C(8)); 102.17 (d, C(9)); 131.43 (d, ^4^*J* = 3.4, C(10)); 130.18 (d, ^3^*J* = 8.4, C(11) and C(15)); 115.90 (d, ^2^*J* = 21.8, C(12) and C(14)); 163.36 (d, ^1^*J* = 250.1, C(13)); 70.43 (t, C(16)); 135.62 (s, C(17)); 127.37 and 128.65 (2d, C(18), C(22) and C(19), C(21)); 128.28 (d, C(20)).
*7-(3-((1R,5S)-6,6-Dimethylbicyclo[3.1.1]hept-2-en-2-yl)propoxy)-4-(4-methoxyphenyl)-2H-chromen-2-one* **17e** Yield 40 %, Method b. HRMS: 429.2055 [M − H]^+^; calcd. 429.2060 (C_28_H_29_O_4_). ^1^H-NMR (CDCl_3_, *δ* ppm, *J*, Hz): 0.81 (s, 3H-C(28)); 1.12 (d, 1H, ^2^*J* = 8.5, H-C(26a)); 1.25 (s, 3H-C(27)); 1.78–1.91 (m, 2H-C(18)); 2.01 (ddd, 1H, *J*(25,26s) = *J*(25,23) = 5.6, *J*(25,21) = 1.4, H-C(25)); 2.04–2.12 (m, 3H, 2H-C(19), H-C(23)); 2.16 (dm, 1H, ^2^*J* = 17.5, H-C(22)); 2.23 (dm, 1H, ^2^*J* = 17.5, H’-C(22)); 2.35 (ddd, 1H, ^2^*J* = 8.5, *J*(26s,23) = *J*(26s,25) = 5.6, H-C(26s)); 3.85 (c, 3H-C(16)); 3.98 (t, 2H, *J*(17,18) = 6.5, 2H-C(17)); 5.20–5.23 (m, H-C(21)); 6.14 (s, H-C(3)); 6.76 (dd, 1H, *J*(7,6) = 8.9, *J*(7,9) = 2.5, H-C(7)); 6.83 (d, 1H, *J*(9,7) = 2.5, H-C(9)); 7.00 (br.d, 2H, *J*(12,11) = *J*(14,15) = 8.6, H-C(12), H-C(14)); 7.36 (br.d, 2H, *J*(11,12) = *J*(15,14) = 8.8, H-(C-11), H-C(15)); 7.40 (d, 1H, *J*(6,7) = 8.9, H-C(6). ^13^C-NMR (CDCl_3_, δ_C_): 155.91 (s, C(1)); 160.61 (s, C(2)); 111.05 (d, C(3)); 155.37 (s, C(4)); 112.34 (s, C(5)); 127.78 (d, C(6)); 112.48 (d, C(7)); 162.14 (s, C(8)); 101.43 (d, C(9)); 127.78 (c, C(10)); 129.71 (d, C(11)); 114.12 (d, C(12); 160.61 (c, C(13)); 114.12 (d, C(14); 129.71 (d, C(15)); 55.27 (k, C(16)); 68.13 (t, C(17)); 26.46 (t, C(18)); 32.87 (t, C(19)); 146.94 (c, C(20)); 116.65 (d, C(21)); 31.12 (t, C(22)); 40.70 (d, C(23)); 37.81 (c, C(24)); 45.62 (d, C(25)); 31.56 (t, C(26)); 26.17 (q, C(27)); 21.05 (q, C(28)).
*7-(4-((1R,5S)-6,6-Dimethylbicyclo[3.1.1]hept-2-en-2-yl)butoxy)-4-(4-methoxyphenyl)-2H-chromen-2-one* **17f** Yield 44%, Method **b**. ${\left[\alpha \right]}_{589}^{26}$ = −7.2 (*c* = 1.03, CHCl_3_) HRMS: 443.2222 [M − H]^+^; calcd. 443.2217 (C_29_H_31_O_4_). ^1^H-NMR (CDCl_3_, *δ* ppm, *J*, Hz): 0.81 (s, 3H-C(29)); 1.12 (d, 1H, ^2^*J* = 8.5, H-C(27a)); 1.25 (s, 3H-C(28)); 1.44–1.56 (m, 2H-C(19)); 1.75–1.82 (m, 2H-C(18)); 1.96–2.01 (m, 3H, 2H-C(20), H-C(26)); 2.03–2.07 (m, H-C(24)); 2.16 (dm, 1H, ^2^*J* = 17.4, H-C(23)); 2.23 (dm, 1H, ^2^*J* = 17.4, H’-C(23)); 2.33 (ddd, 1H, ^2^*J* = 8.5, *J*(27s,24) = *J*(27s,26) = 5.6, H-C(27s)); 3.86 (c, 3H, C(16)); 4.00 (t, 2H, *J*(17,18) = 6.5, H-C(17)); 5.17–5.20 (m, 1H, H-C(22)); 6.15 (s, 1H, H-C(3)); 6.76 (dd, 1H, *J*(7,6) = 8.9, *J*(7,9) = 2.5, H-C(7)); 6.84 (d, *J*(9,7) = 2.5, H-C(9)); 7.00 (d, *J*(12,11) = *J*(14,15) = 8.8, 2H, H-C(12), H-C(14)); 7.36 (d, *J*(11,12) = *J*(15,14) = 8.8, 2H, H-(C-11), H-C(15)); 7.41 (d, *J*(6,7) = 8.9, H-C(6)). ^13^C-NMR (CDCl_3_, δ_C_): 155.89 (s, C(1)); 161.31 (s, C(2)); 111.03 (d, C(3)); 155.37 (s, C(4)); 112.48 (s, C(5)); 127.77 (d, C(6)); 112.48 (d, C(7)); 162.12 (s, C(8)); 101.44 (d, C(9)); 127.73 (c, C(10)); 129.71 (d, C(11)); 114.11 (d, C(12); 160.59 (c, C(13)); 114.11 (d, C(14); 129.71 (d, C(15)); 55.27 (q, C(16)); 68.38 (t, C(17)); 28.58 (t, C(18)); 23.33 (t, C(19)); 36.33 (t, C(20)); 147.69 (c, C(21)); 116.13 (d, C(22)); 31.12 (t, C(23)); 40.72 (d, C(24)); 37.79 (c, C(25)); 45.59 (d, C(26)); 31.53 (t, C(27)); 26.20 (q, C(28)); 21.06 (q, C(29)).

#### 3.1.5. Synthesis of 7-Aminocoumarins **21** and **22**

7-Aminocoumarin **18** was synthesized from 3-aminophenol **19** in accordance with [18].

Methoxycarbonyl chloride (3.6 mL, 46.8 mmol) was added dropwise to a cooled (5–10 °C) suspension of 3-aminophenol **19** (4.4g, 40.4 mmol) and K_2_CO_3_ (3.5 g) in 35 mL of ethyl acetate and 3 mL of water with vigorous stirring. The mixture was stirred for 1 h, then 10 mL of water was added, and the mixture was stirred for another 3 h. The product was extracted with ethyl acetate. The extracts were washed with water, 1M H_2_SO_4_, water and brine, dried with Na_2_SO_4_, and evaporated. The resulting solid was crystallized from benzene to give 5.7 g of **20** (77%).

A mixture of compound **20** (4.6 g, 28 mmol) and 5.0 mL ether **7a** was added dropwise to 6 mL H_2_SO_4_ with vigorous stirring. The mixture was stirred for 3 h and diluted with 50 mL of ice water. The precipitate was removed by filtration, washed with water, MeOH, and ether, and then dried to give 3.62 g of **18a** (44%).

A suspension of compound **18a** (2.45 g, 8.3 mmol) in 5 mL of 45% KOH solution was stirred at 90 °C for 0.5 h until the solution formed. The mixture was cooled and diluted with water and acidified with concentrated HCl to pH 5–6. A solution of alkali was added to the suspension to pH 8. The mixture was stirred until crystallization ceased. The precipitate was removed by filtration, washed with water, MeOH, ether, and then dried to give 1.62 g of **18** (88%).

^1^H NMR data for compound 18 correspond to those published earlier [22].

Amines **21** and **22** were obtained by the interaction of compound **18** and (−)-myrtenal and (−)-nopinal (synthesized by the oxidation of (−)-nopol with IBX according to the procedure [23]) and subsequent reduction with NaBH_3_CN in accordance with [24]. 

Compound **18** (0.190 g, 0.8 mmol), (−)-myrtenal (0.180 g, 1.2 mmol) and acetic acid (150 μL) were dissolved in methanol (5 mL) and stirred at room temperature for 2.5 h. Then, NaBH_3_CN (0.165 g, 3.0 mmol) was added and the reaction mixture was stirred at room temperature for 1.5 h. Methanol was evaporated and the reaction mixture was extracted with CH_2_Cl_2_. The organic layer was washed with brine, dried over anhydrous Na_2_SO4, filtered, and evaporated. The residue was crystallized from ethanol to give 0.119 g of **21** (yield—40%).

Similarly, compound **22** was synthesized from amine **18** and (−)-nopinal (51%).

*7-(((1R,5S)-6,6-Dimethylbicyclo[3.1.1]hept-2-en-2-yl)methylamino)-4-phenyl-2H-chromen-2-one* **21** Yield 33%, Method a. M.p. 141 °C. ${\left[\alpha \right]}_{589}^{27}$ = −28.14 (*c* = 0.73, CHCl_3_). HRMS: 371.1877 [M]^+^; calcd. 371.1880 (C_25_H_25_O_2_N_1_). ^1^H-NMR (CDCl_3_, *δ* ppm, *J*, Hz): 0.80 (s, 3H-C(26)); 1.14 (d, 1H, ^2^*J* = 8.6, H-C(24a)); 1.26 (s, 3H-C(25)); 2.05–2.12 (m, 2H, H-C(21), H-C(23)); 2.20 (dm, 1H, ^2^*J* = 17.7, H-C(20)); 2.27 (dm, 1H, ^2^*J* = 17.7, H’-C(20)); 2.37 (ddd, 1H, ^2^*J* = 8.6, *J*(24s,21) = *J*(24s,23) = 5.6, H-C(24s)); 3.64–3.71 (br.s, 2H-C(17)); 4.72 (br. s, 1H, H-N(16)), 5.42–5.46 (m, H-C(19)); 6.03 (s, H-C(3)); 6.44 (dd, 1H, *J*(7,6) = 8.8, *J*(7,9) = 2.1, H-C(7)); 6.53 (d, 1H, *J*(9,7) = 2.1, H-C(9)); 7.19 (d, 1H, *J*(6,7) = 8.7, H-C(6)); 7.38–7.42 (m, 2H, H-(C-11), H-C(15)); 7.44–7.49 (3H, H-C(12), H-C(13), H-C(14)). ^13^C-NMR (CDCl_3_, δ_C_): 156.47 (s, C(1)); 161.86 (s, C(2)); 108.98 (d, C(3)); 156.10 (s, C(4)); 109.38 (s, C(5)); 127.63 (d, C(6)); 110.59 (d, C(7)); 151.41 (s, C(8)); 98.60 (d, C(9)); 135.97 (s, C(10)); 128.24 (d, C(11)); 128.52 (d, C(12); 129.14 (d, C(13)); 128.52 (d, C(14); 128.24 (d, C(15)); 48.30 (t, C(17)); 143.75 (s, C(18)); 118.82 (d, C(19)); 30.99 (t, C(20)); 40.67 (d, C(21)); 38.02 (s, C(22)); 43.81 (d, C(23)); 31.45 (t, C(24)); 25.99 (q, C(25)); 21.01 (q, C(26)).
*7-(2-((1R,5S)-6,6-Dimethylbicyclo[3.1.1]hept-2-en-2-yl)ethylamino)-4-phenyl-2H-chromen-2-one* **22** Yield 51%, Method b. ${\left[\alpha \right]}_{589}^{27}$ = −17.70 (*c* = 0.73, CHCl_3_) HRMS: 385.2033 [M]^+^; calcd. 385.2036 (C_26_H_27_O_2_N_1_). ^1^H-NMR (CDCl_3_, *δ* ppm, *J*, Hz): 0.80 (s, 3H-C(27)); 1.10 (d, 1H, ^2^*J* = 8.6, H-C(25a)); 1.26 (s, 3H-C(26)); 2.03 (ddd, 1H, H-C(24); 2.06–2.11 (m, 2H, H-C(22)); 2.20 (dm, 1H, ^2^*J* = 17.7, H-C(21)); 2.27 (dm, 1H, ^2^*J* = 17.7, H’-C(21)); 2.30–2.38 (m, 3H, 2H-C(18), H-C(25s)); 3.13–3.23 (m, 2H-C(17)); 5.32–5.35 (m, H-C(20)); 6.07 (s, H-C(3)); 6.54 (d, 1H, *J*(7,6) = 8.8, H-C(7)); 6.64 (d, 1H, *J*(9,7) = 2.1, H-C(9)); 7.23 (d, *J*(6,7) = 8.7, H-C(6)); 7.37–7.42 (m, 2H, H-C(11), H-C(15)); 7.44–7.50 (m, 3H, H-C(12), H-C(13), H-C(14)). ^13^C-NMR (CDCl_3_, δ_C_): 156.35 (s, C(1)); 161.47 (s, C(2)); 109.82 (d, C(3)); 155.90 (s, C(4)); 110.58 (s, C(5)); 127.87 (d, C(6)); 111.50 (d, C(7)); 154.76 (s, C(8)); 115.69 (d, C(9)); 135.79 (s, C(10)); 128.23 (d, C(11)); 128.58 (d, C(12); 129.25 (d, C(13)); 128.58 (d, C(14); 128.23 (d, C(15)); 41.95 (t, C(17)); 35.42 (t, C(18)); 144.52 (s, C(19)); 119.31 (d, C(20)); 31.20 (t, C(21)); 40.56 (d, C(22)); 37.87 (s, C(23)); 45.15 (d, C(24)); 31.61 (t, C(25)); 26.06 (q, C(26)); 21.09 (q, C(27)).

### 3.2. Biology

Cells and viruses were obtained from the working collection of the laboratory of chemotherapy for viral infections of the Smorodintsev Research Institute of Influenza (Saint-Petersburg, Russia).

#### 3.2.1. Cytotoxicity Test

The compounds were weighed in an amount of 2 mg and dissolved in 100 μL of DMSO. Then, the resulting solution was adjusted with the medium to a concentration of 1000 μg/mL, and a series of 2-fold dilutions was prepared from it. One-day culture of HEp2 cells, grown in 96-well plates, cell concentration 3 × 10^5^/well of the plate, was checked visually in an inverted microscope for the integrity of the monolayer. Plates were selected for work, where the cell closure was 60–80%.

Dilutions of the compounds at the appropriate concentration were added to the plate in a volume of 100 μL in each well in 2 replicates for each tested concentration. The plates were incubated for 24 h at 37 °C in the presence of 5% CO_2_. Cell viability was assessed using the MTT test.

The MTT solution was prepared on a maintenance medium at a concentration of 0.5 mg/mL. Then, 0.1 mL of MTT solution was added to each well. After 1.5 h of MTT contact at 37 °C at a CO_2_ concentration of 5%, MTT was discarded with the cells of the well and 0.1 mL of ethyl alcohol 96% was poured, after which the optical density in the wells was measured at a wavelength of 535 nm. Based on the data obtained, the CC_50_ was calculated.

#### 3.2.2. Antiviral Activity

The antiviral activity against the respiratory syncytial virus was assessed in a series of 3-fold dilutions of test compounds, starting from ½CC_50_, which were added to HEp-2 cell culture, at a double concentration, 100 μL per well, followed by addition of 100 μL of the virus in a series of 10-fold dilutions. Cells were incubated at 37 °C and 5% CO_2_ for 1 h. Then, the virus was washed out, and the compounds were again added at a single concentration and incubated at 37 °C and 5% CO_2_ for 6 days. For the enzyme-linked immunosorbent assay (ELISA), cell culture was fixed with cold 80% acetone at −20 °C for 15 min, and then washed with phosphate buffered saline containing 0.05% Tween 20. Next, a solution of primary mouse anti-RSV F protein antibodies was added to the culture and incubated at room temperature under continuous stirring for 2 h. Then, cells were again washed with buffer, secondary anti-mouse antibodies were added, and the cells were incubated under continuous stirring for 2 h. Then, the antibodies were washed off, and a substrate-chromogenic mixture with tetramethylbenzidine was added. After 5 min, the reaction was stopped with 0.1 M sulfuric acid, and optical density of the solution was measured at a wavelength of 450 nm. Wells with absorbance values two fold or greater than the cell control were considered to be contaminated. The virus titer was calculated using the Reed and Muench method. All experiments were made in triplicate.

#### 3.2.3. Time-of-addition Assay

Compounds were added at different time points before, after, or simultaneously with the introduction of the virus. The time of addition of the compounds was counted from Point 0, i.e., the time of entry of the virus into the cell. During the period (−1)–0, the cells together with the virus were incubated at 40 °C. All other experiments were carried out at 37 °C. RSV virus A 2 mL was added to the cells at a time that was conventionally designated as Point 1, after which the cells were kept for an hour at a temperature of 40 °C. Then, at Point 0, the virus was unbound. The cells were transferred to a thermostat at 37 °C, where they were incubated for 25 h. After this period, the medium was taken from each well and a series of ten-fold dilutions were made on a fresh cell culture and incubated for 6 days. For each compound, 2 repetitions were made by different operators. The virus titer was estimated by ELISA. The compounds were added at the following times relative to the addition of the virus: Point 2, the compounds were introduced one hour before cell infection (prophylactic regimen); Point 0, at the moment of temperature change; Points 1, 2, 4, 6, and 24, after 1, 2, 4, 6, and 24 h after the temperature change, respectively. In the wells marked (−2)–(25), the compounds were kept throughout the experiment, starting from Point 2 and until the end of the experiment, i.e., 25 h. No compounds were added to the control wells; instead, a similar volume of medium was added.

### 3.3. Molecular Modeling Study

All theoretical calculations were carried out using software Schrodinger Small Molecule Drug Discovery Release 2022-1.

#### 3.3.1. Protein and Ligands Preparations

The geometrical parameters of full-size F protein (PDB code 7LVW [25] of human RVS A2 were downloaded from non-commercial database Protein Data Bank [26]. To localization of the binding site complex protomer, inhibitor RV521 was used (PDB code 7KQD [7]. Model structures were prepared using plugin Protein PrepWizard: hydrogen atoms were added and minimized, side chains of amino acids were edited, multiplicity of chemical bonds were restored, water molecules were removed. Geometric parameters were optimized in the OPLS4 force field [27]. The geometric parameters of the ligands were optimized by the OPLS4 force field method [27], considering possible conformers.

#### 3.3.2. Binging Site Analysis

A cavity located in the central part of the F protein was considered as a likely binding site. According to [20], most known F protein inhibitors (BMS-43371, JNJ-240868, rilematovir, and others) bind in the cavity. The binding site contains a number of aromatic and hydrophobic a.a. of fusion loops (FP) and heptadic repeat (HBR), namely Phe140, Lue141, Asp486, Glu487, and Phe488. The pharmacophore linking site profile was created and described using the Phase plugin [28].

#### 3.3.3. Molecular Docking

Molecular docking of all compounds was carried out using the induced fit docking (IFD) protocol with the following conditions: flexible protein and ligand, grid matrix size of 15 Å, amino acids within a radius of 5Å from the ligand were optimized considering the influence of the ligand. The ranking of docking solutions was carried out by evaluating the following calculation parameters: docking score (based on GlideScore minus fines), ligand efficiency (LE, which took into account the atomic distribution of the scoring function), as well as the parameter of the model energy value (Emodel), including the GlideScore value, the energy of unrelated interactions, and the parameters of the energy spent on the formation of the stacking of the connection in the binding site.

#### 3.3.4. Molecular Dynamics

Trimer–ligand complexes for the two leader compounds were used for subsequent simulations of molecular dynamics. Given the structure of the full-length F protein, the transmembrane domain (525–550 a.o.) was placed in the POPC membrane (phosphatidylcholine) [8]. Phosphatidylcholine is part of most cell membranes of viruses [29,30]. The complexes were placed in an orthorhombic box (size of 15 × 35 × 55 Å) and filled with 0.15 M of aqueous solution of NaCl. The solvent model is TIP3P. Counterions were added to the system to maintain neutrality. The thermodynamic ensemble is NPAT. The period of recorded simulation of dynamics was 100 nanoseconds at a temperature of 310 K (37 °C). The protocol for preparing the system for simulation included a preliminary minimization and balancing of system components.

## 4. Conclusions

A set of monoterpene-substituted arylcoumarins was synthesized and tested as antiviral agents against respiratory syncytial virus (RSV). It was shown, for the first time, that these compounds are efficient RSV replication inhibitors. The most active compound **14e** has a selectivity index of about 200 and acts most effectively at the early stages of infection. Based on molecular docking and molecular dynamics simulation data, the F protein of RSV was proposed as a potential target for these compounds.

## Figures and Tables

**Table 1:** Antiviral activity and cytotoxicity of compounds **14**–**17**, **21,** and **22** against RSV A.

*N*	CC_50_ *, µM	EC_50_ **, µM	SI ***	*N*	CC_50_, µM	EC_50_, µM	SI
**14a**	805 ± 97.6	67.3 ± 9.1	**11.9**	**16a**	273 ± 16	47.4 ± 8	5.8
**14b**	591 ± 85.2	18.0 ± 0.9	**32.8**	**16b**	297 ± 8.5	102.9 ± 7.1	2.9
**14c**	813 ± 32.4	233.6 ± 27.1	3.5	**16c**	20.5 ± 4.9	14.3 ± 2.9	1.4
**14d**	657 ± 20.4	82.5 ± 10.3	7.9	**16e**	996 ± 8.2	109.9 ± 10.4	9
**14e**	1443 ± 85.1	7.5 ± 0.4	**192.6**	**16f**	109 ± 10.5	9.2 ± 1.1	**11.8**
**14f**	1267 ± 180	110.9 ± 10.5	11.4	**16g**	655 ± 29.7	>690	<0.9
**14g**	1544 ± 73.7	>1580	<1	**17a**	339 ± 26.3	58.3 ± 8.5	5.8
**15a**	8.8 ± 0.5	8.8 ± 0.7	1	**17b**	12.4 ± 2.8	>12	<1
**15b**	46.6 ± 8.6	15.1 ± 1.1	3	**17c**	14.7 ± 2.9	>15	<1
**15c**	90.8 ± 11	48.7 ± 7.6	1.9	**17d**	677 ± 22.9	64.1 ± 5.4	**10.6**
**15d**	17.2 ± 2.6	>20	<0.8	**17e**	>100	35.9 ± 5.7	2.8
**15e**	12.5 ± 1.4	>12.5	<1	**17f**	117 ± 7	10.8 ± 1	**10.8**
**15f**	91.3 ± 9.2	9.5 ± 0.8	9.6	**17g**	16.8 ± 3.2	>17	<1
**15g**	265 ± 29.5	84.0 ± 3	3.2	**21**	20.5 ± 1.4	>13	<1.5
Ribavirin	>4000	80.1 ± 13.5	**50**	**22**	480 ± 18.4	79.9 ± 7.4	6

* CC_50_—50% cytotoxic concentration,** EC_50_—50% effective concentration, *** SI—selective index.

## Data Availability

The data presented in this study are available on request from the corresponding author.

## Supplementary Materials

The following supporting information can be downloaded at https://www.mdpi.com/article/10.3390/molecules28062673/s1: NMR ^1^H and ^13^C spectra of the compounds **14**–**17**, **21,** and **22**. Table S1: Analysis of structural descriptors and pharmacophoric features of the ligand as a function of linker length, Table S2: Analysis of structural descriptors and pharmacophoric features of the ligand as a function of the nature of the substituent, Table S3: Analysis of structural descriptors and pharmacophoric features of the different stereoisomer, Table S4: Molecular docking results, Figure S1: RMSD fluctuations, Figure S2: The total number of protein-ligand contacts.

## Sample Availability

Samples of the compounds presented in this study are available on request from the authors.
