# Supplementary material for: New Inhibitors of Respiratory Syncytial Virus (RSV) Replication Based on Monoterpene-Substituted Arylcoumarins
Source: Molecules. 2023 Mar 15;28(6):2673. doi: 10.3390/molecules28062673 (PMC10054240; doi:10.3390/molecules28062673)
Supplement: Supplementary file 1 [file molecules-28-02673-s001.zip › molecules-2162759-supplementary.pdf]

Supplementary Information

New Inhibitors of Respiratory Syncytial Virus (RSV) Replication Based on Monoterpene-Substituted Arylcoumarins

Tatyana M. Khomenko [1], Anna A. Shtro [2], Anastasia V. Galochkina [2], Yulia V. Nikolaeva [2], Anzhelika V. Garshinina [2], Sophia S. Borisevich [3,4], Dina V. Korchagina [1], Konstantin P. Volcho [1,*] and Nariman F. Salakhutdinov [1]

[1] Department of Medicinal Chemistry, N.N. Vorozhtsov Novosibirsk Institute of Organic Chemistry, Acad. Lavrentjev Ave. 9, 630090 Novosibirsk, Russia
[2] Laboratory of chemotherapy for viral infections, Smorodintsev Research Intitute of Influenza, 197376 Saint-Petersburg, Russia
[3] Laboratory of Physical Chemistry, Ufa Chemistry Institute of the Ufa Federal Research Center, 71 Octyabrya pr., 450054 Ufa, Russia
[4] Institute of Cyber Intelligence Systems, National Research Nuclear University MEPhI, 115409 Moscow, Russia
* Correspondence: volcho@nioch.nsc.ru

1. NMR ^{1}H and ^{13}C spectra of the compounds **14-17, 21 and 22.**
2. Table S1: Analysis of structural descriptors and pharmacophoric features of the ligand as a function of linker length.
3. Table S2: Analysis of structural descriptors and pharmacophoric features of the ligand as a function of the nature of the substituent
4. Table S3: Analysis of structural descriptors and pharmacophoric features of the different stereoisomers
5. Table S4: Molecular docking results
6. Figure S1: RMSD fluctuations
7. Figure S2: The total number of protein-ligand contacts

Compound **14a**

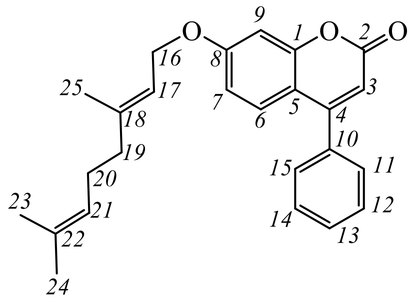

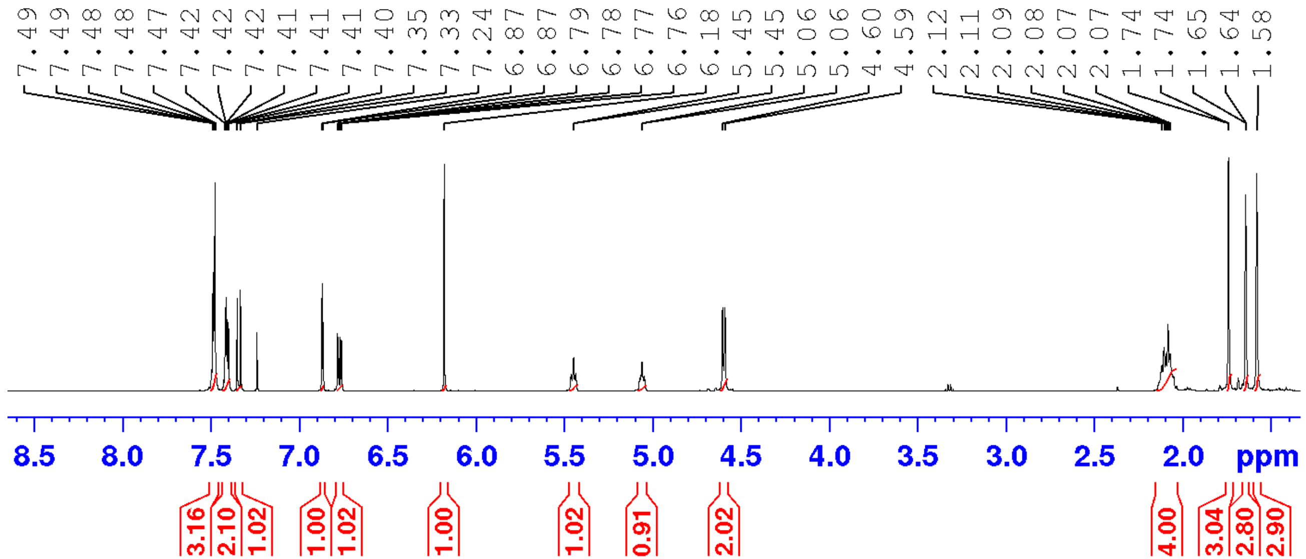

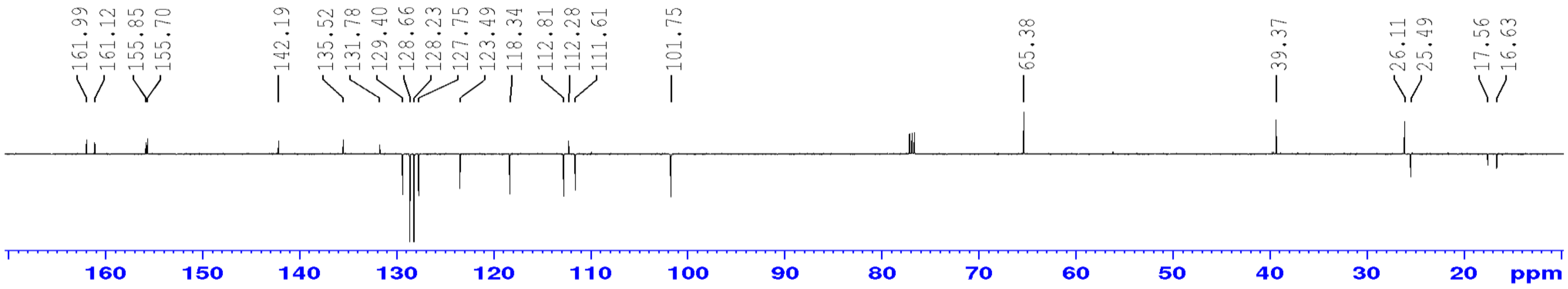

Compound **14b**

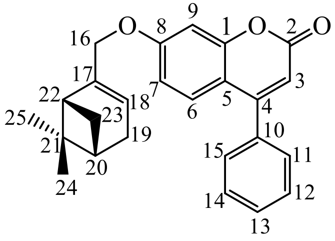

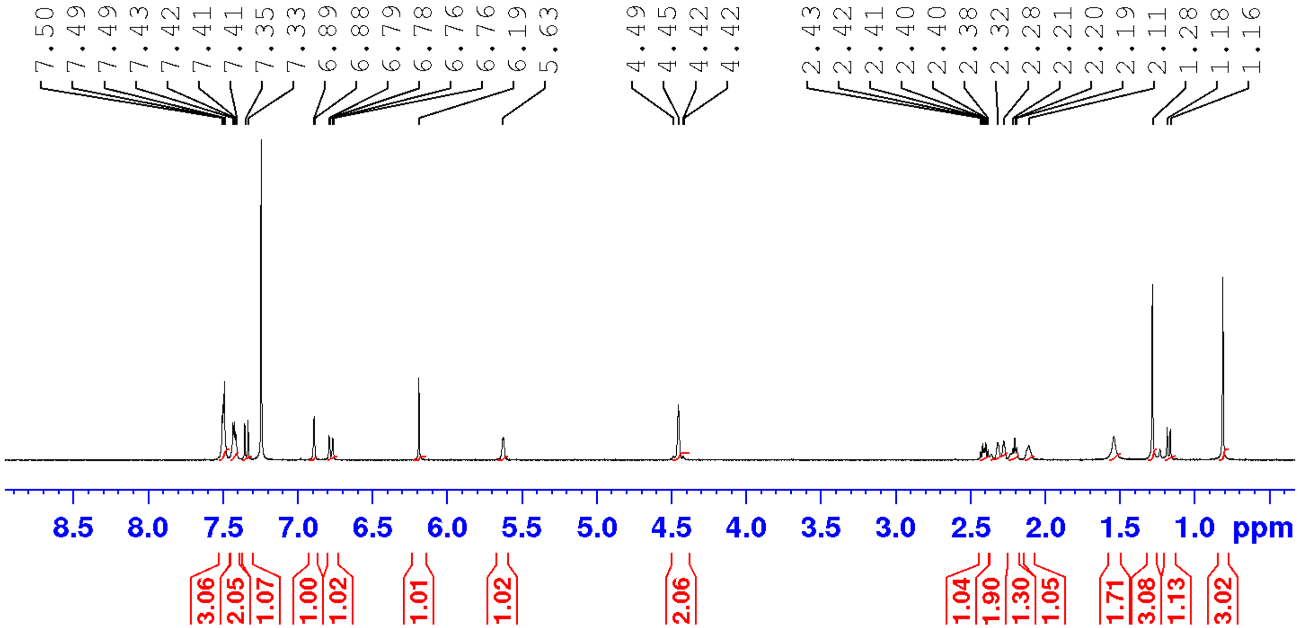

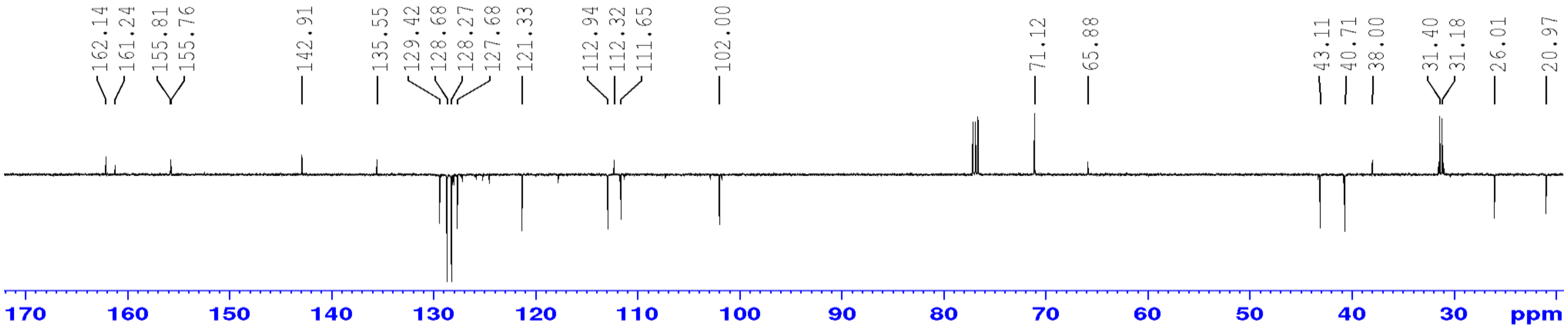

Compound **14d**

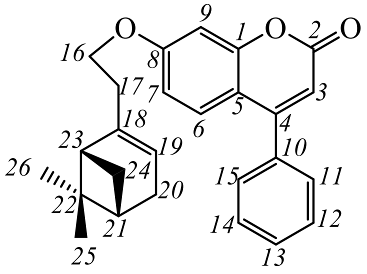

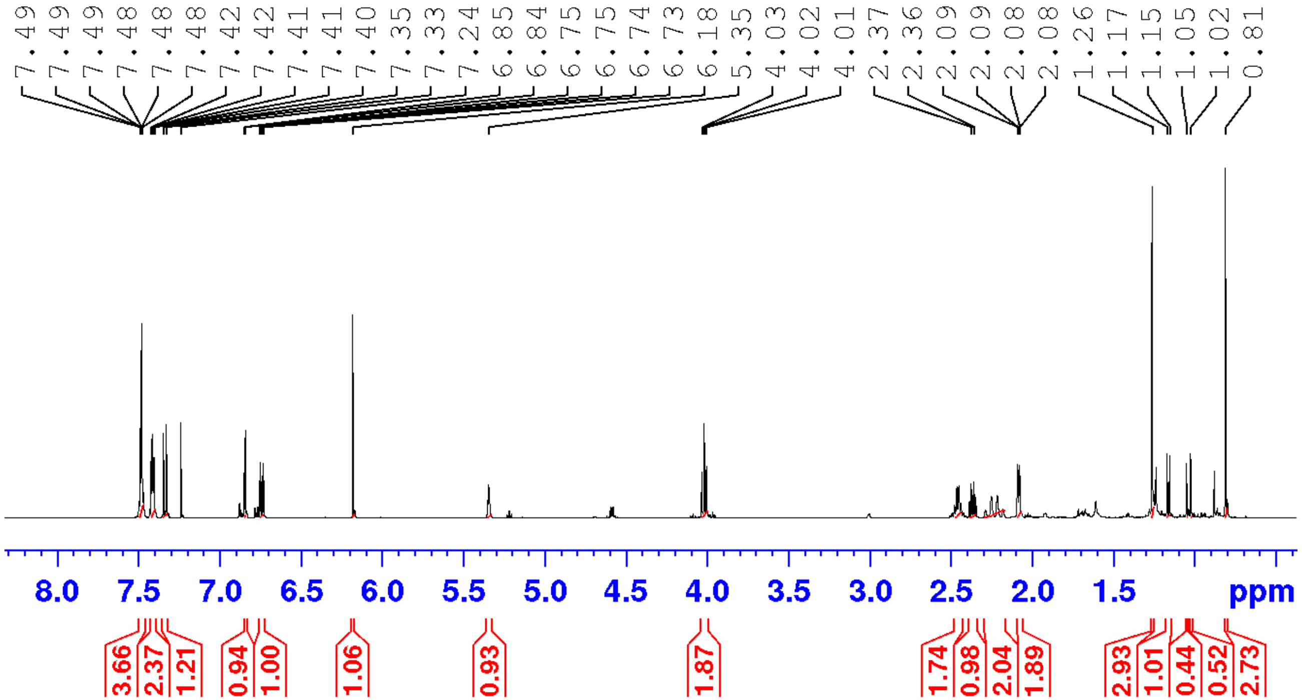

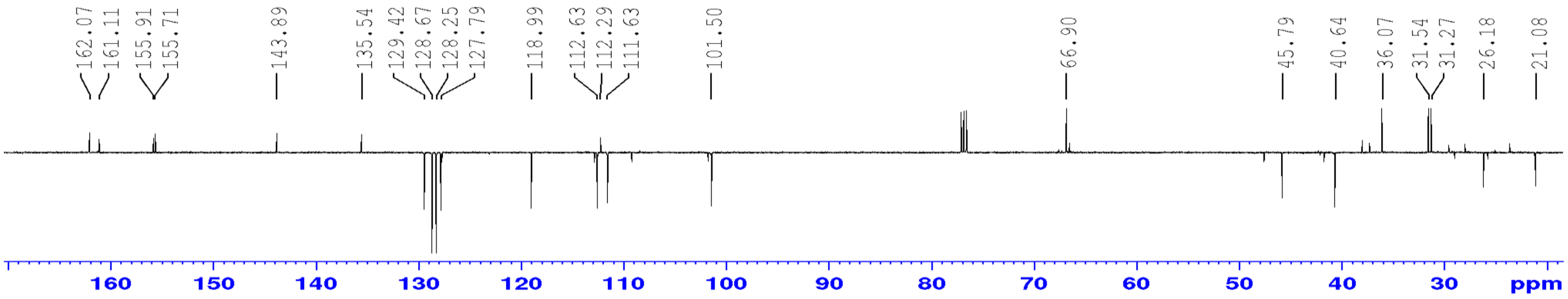

Compound **14e**

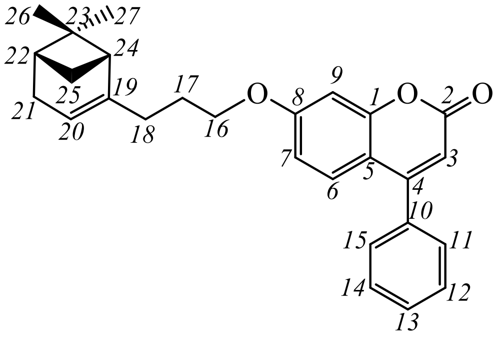

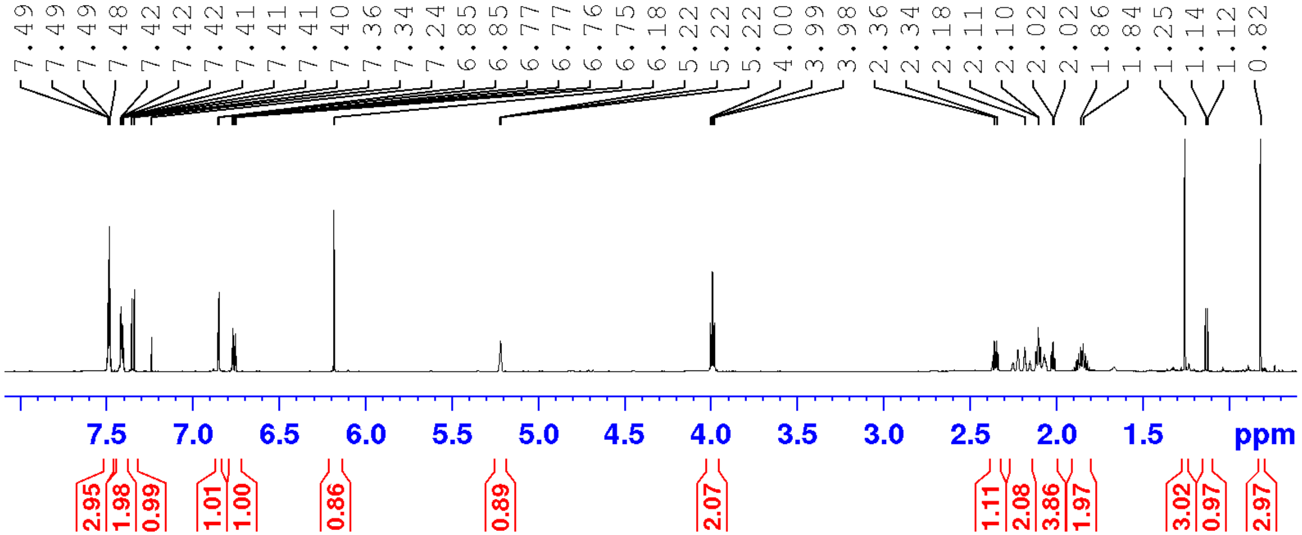

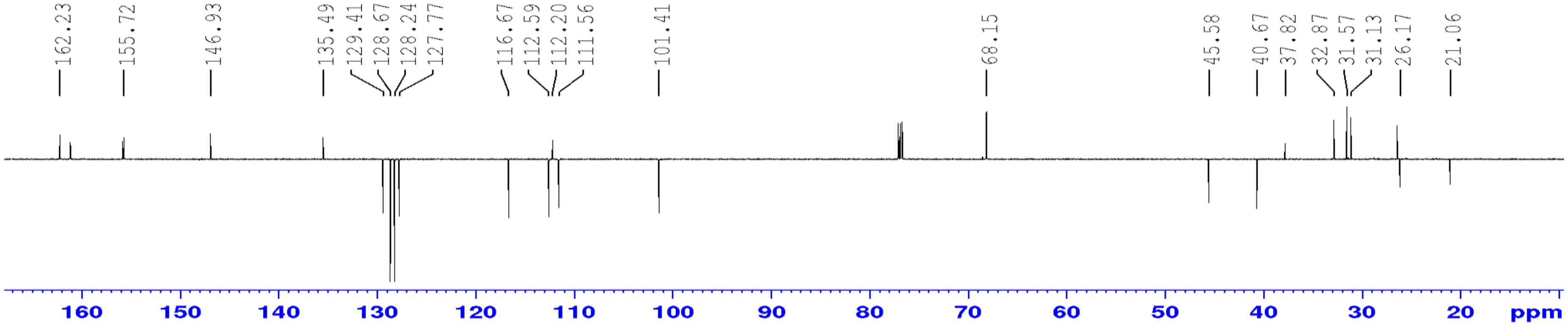

Compound **14f**

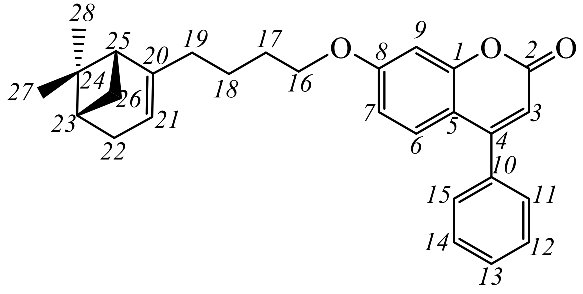

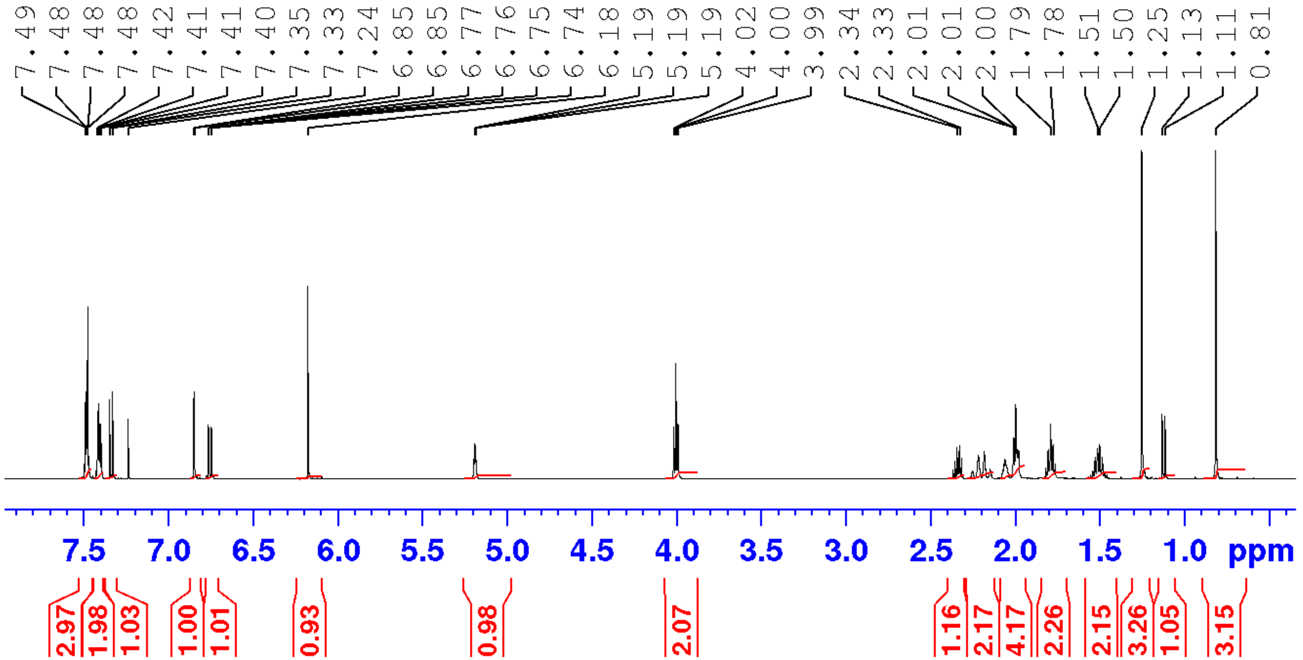

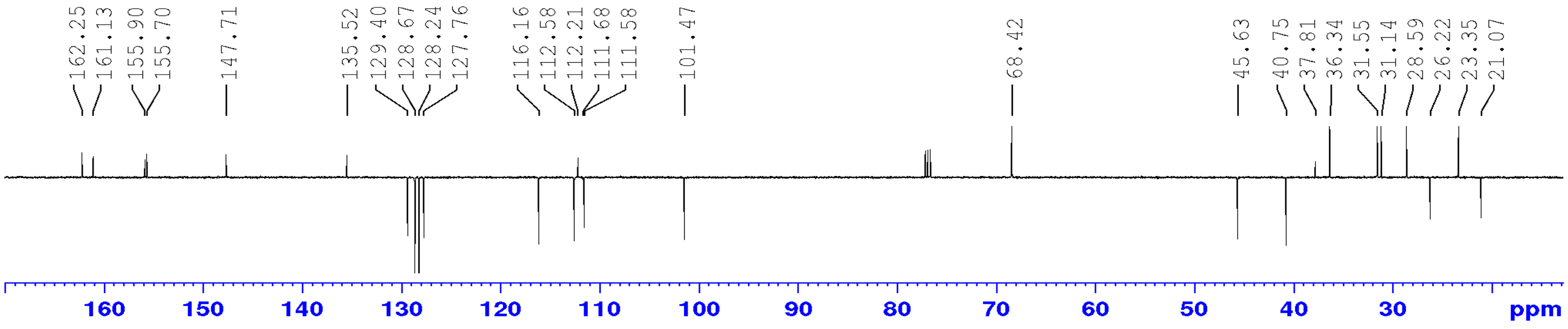

Compound **14g**

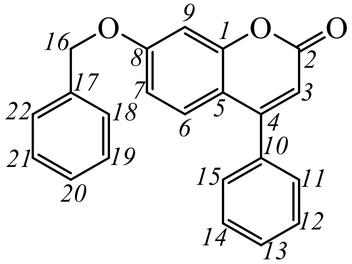

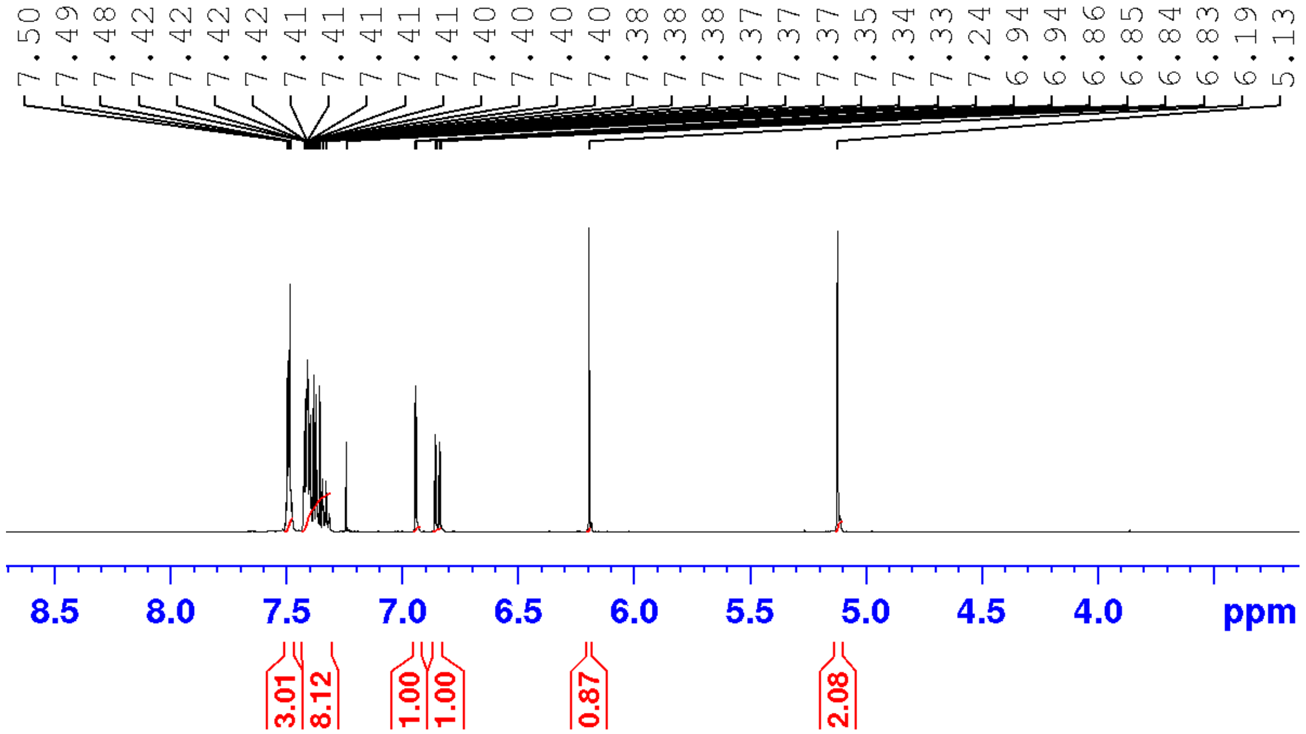

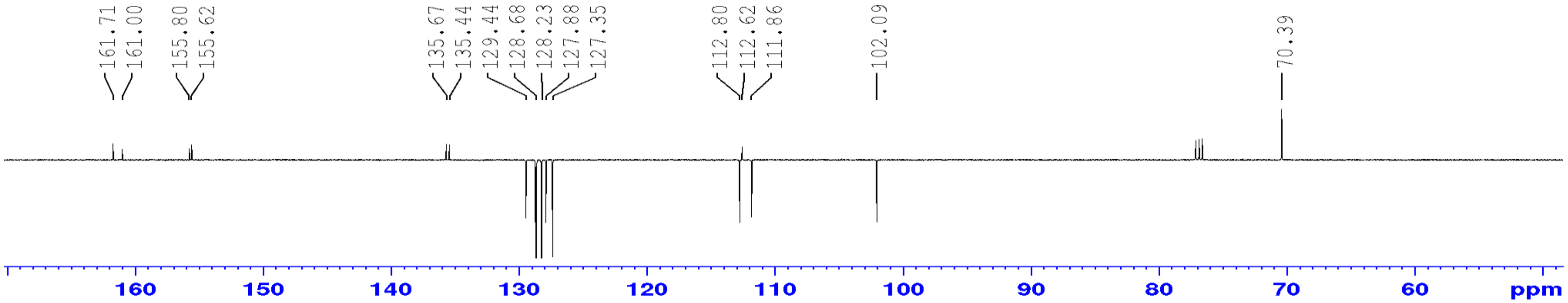

Compound **15a**

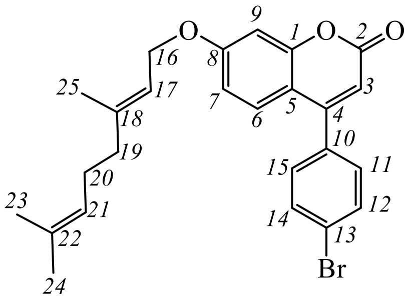

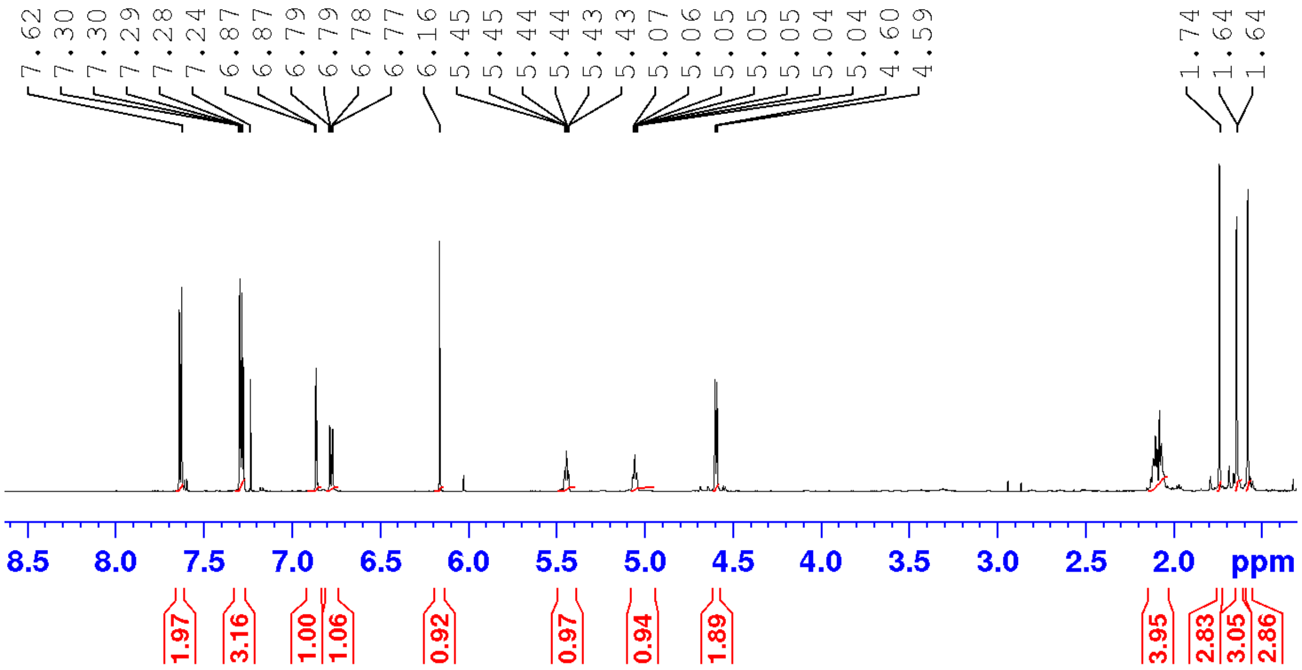

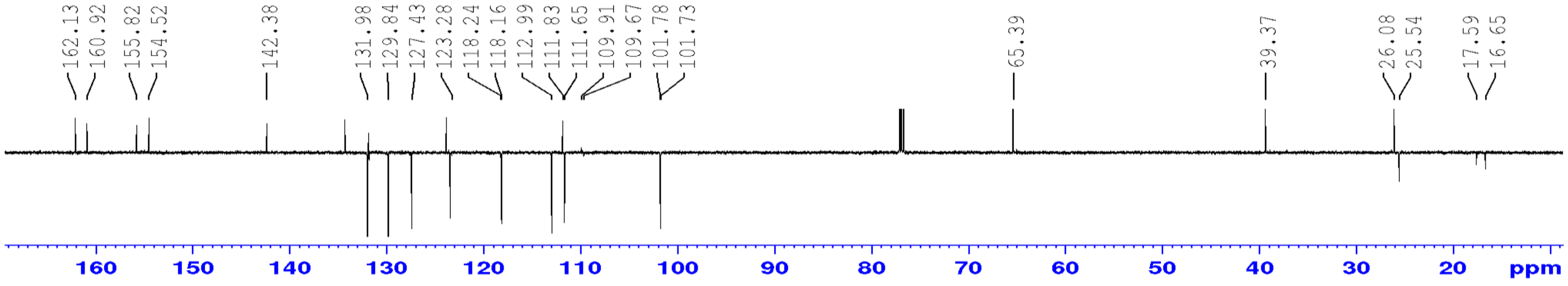

Compound **15b**

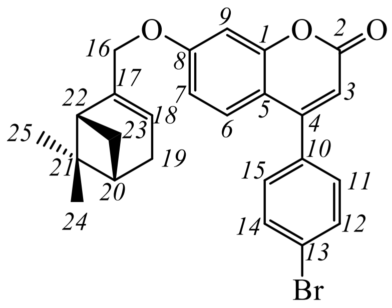

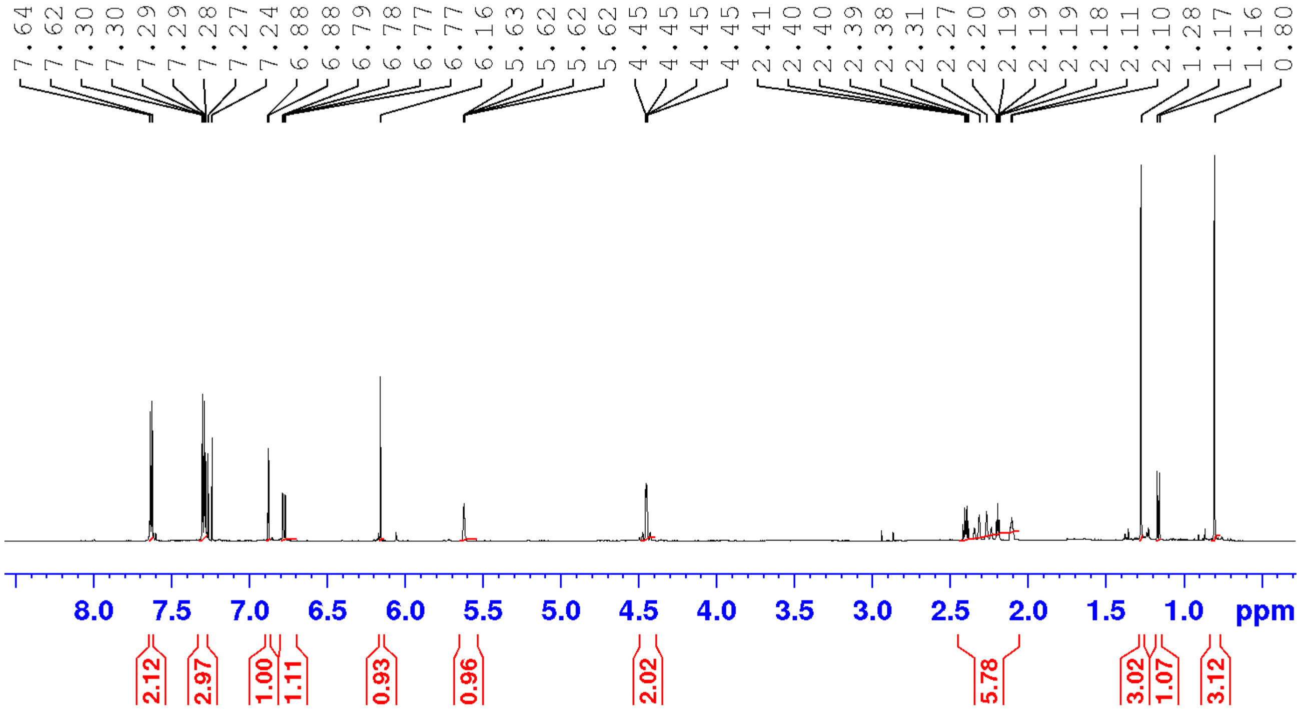

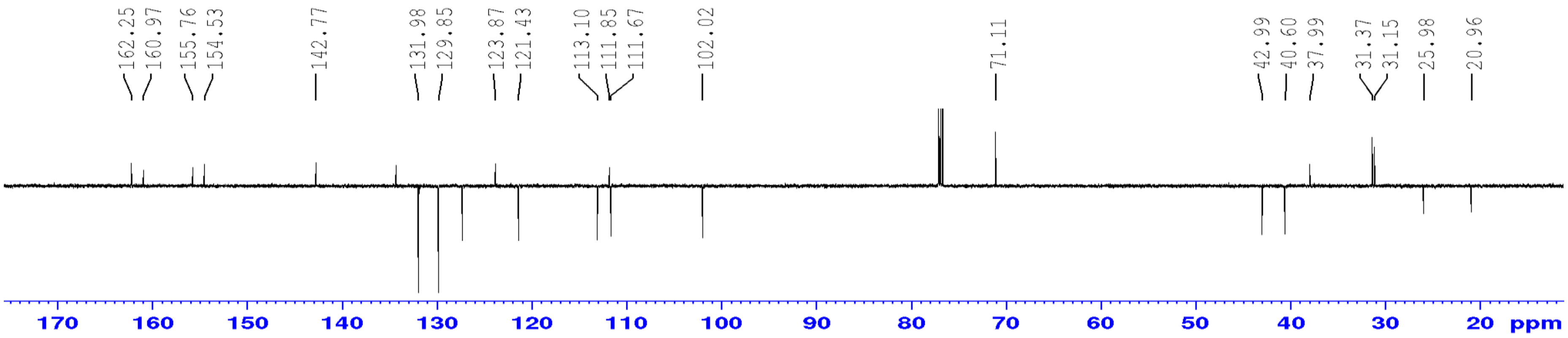

Compound **15d**

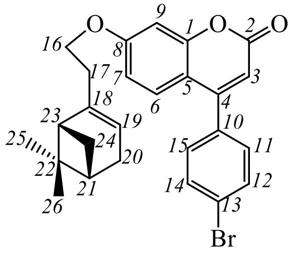

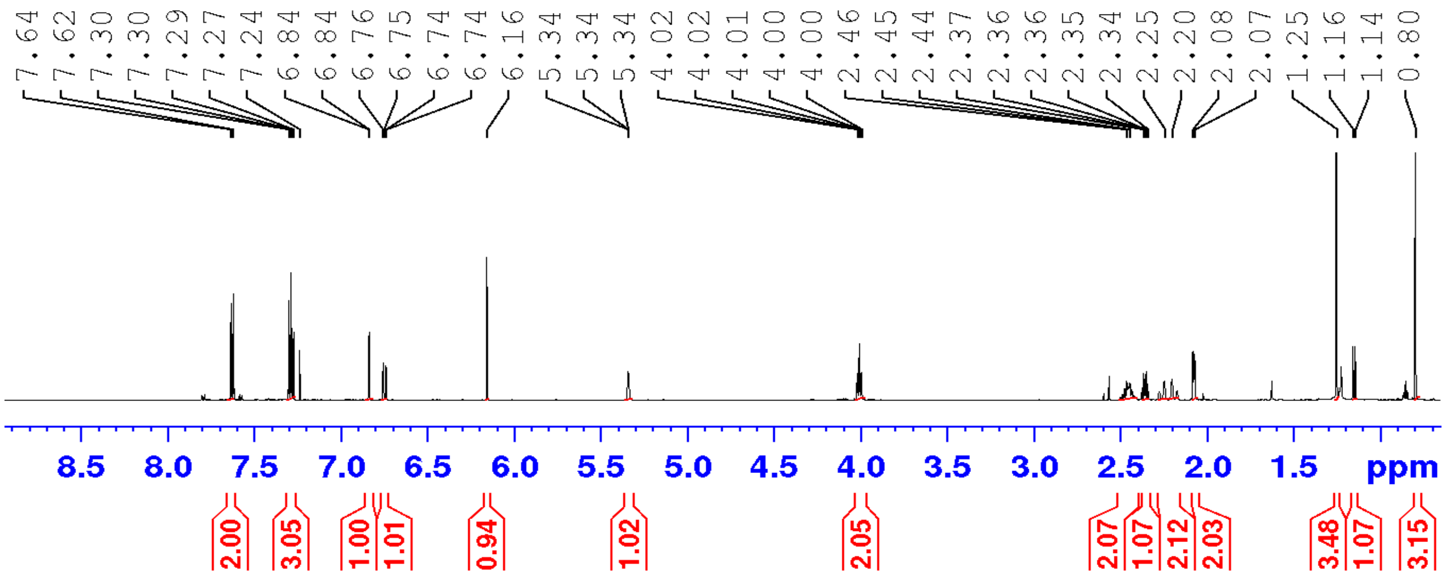

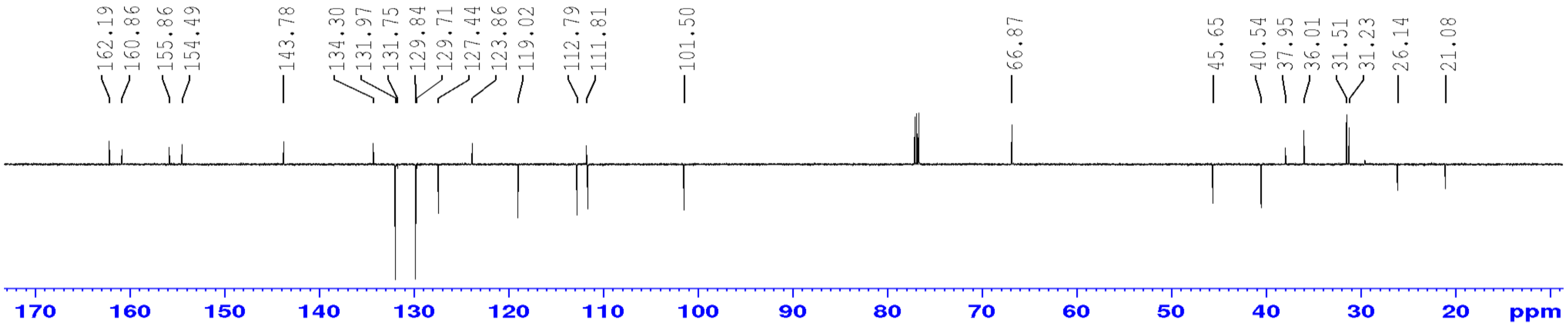

Compound **15e**

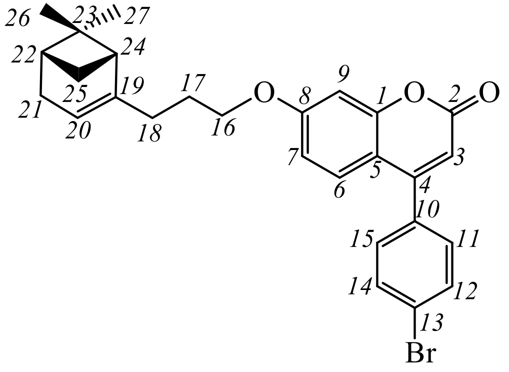

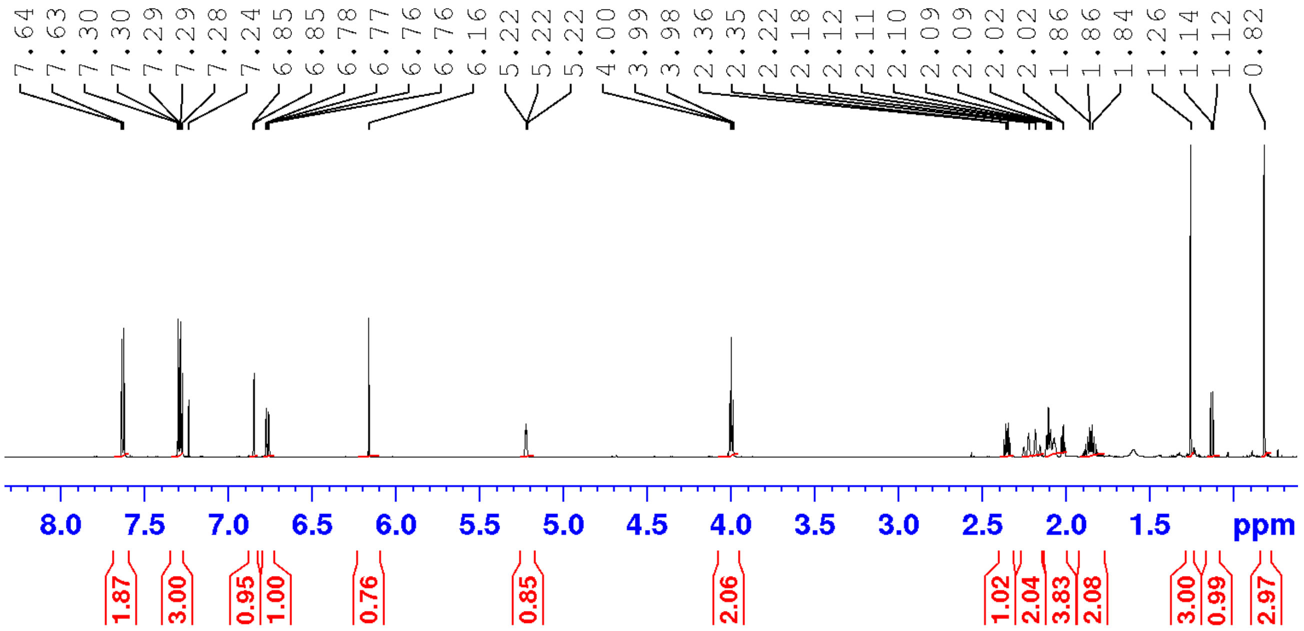

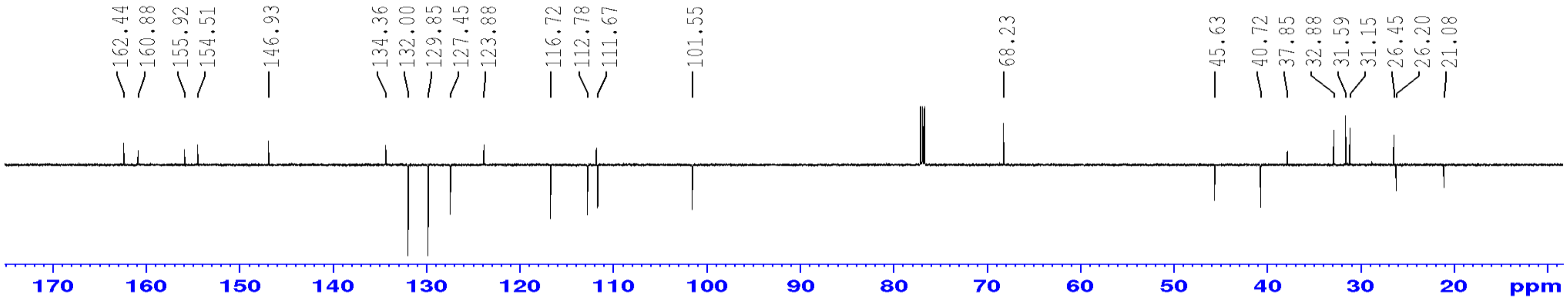

Compound **15f**

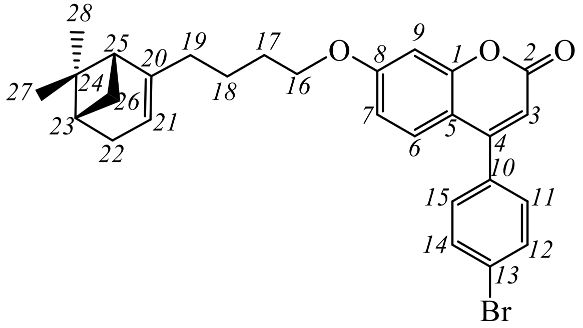

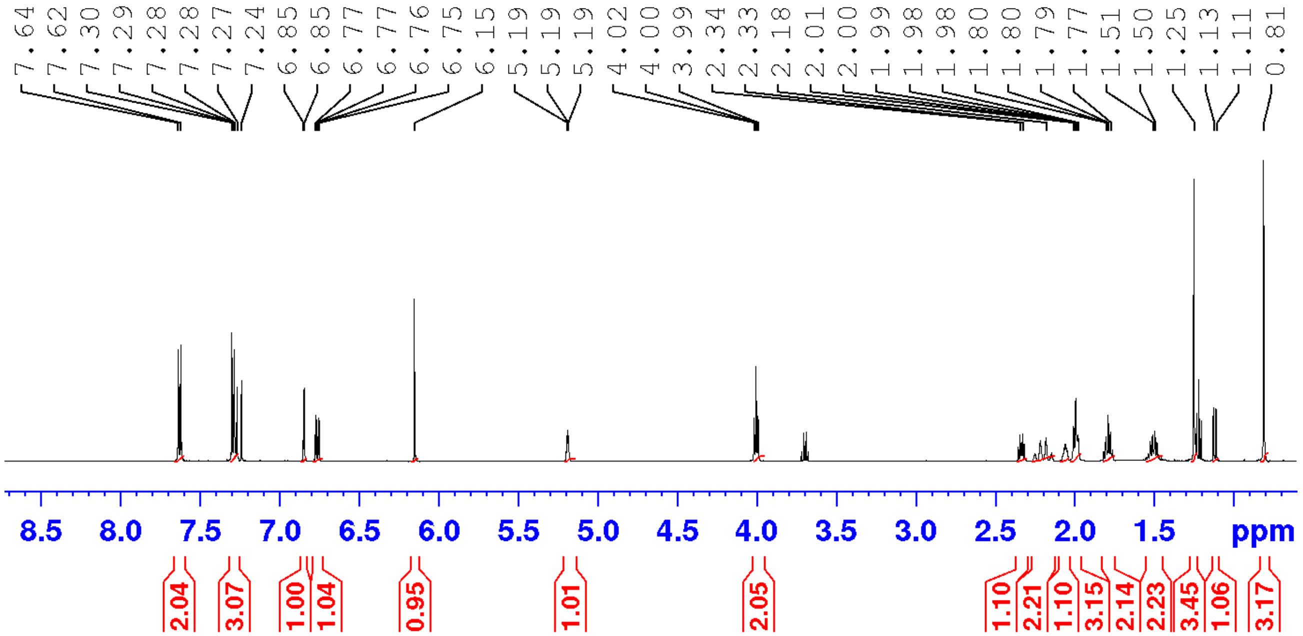

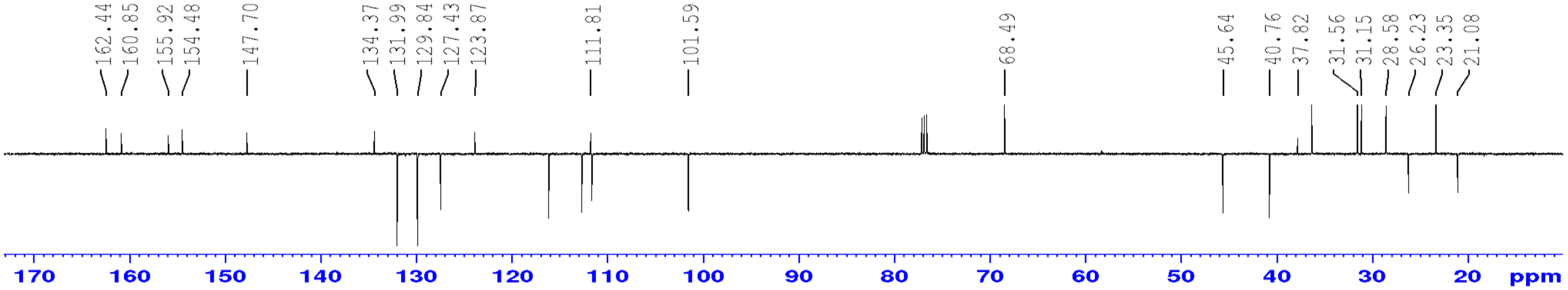

Compound **15g**

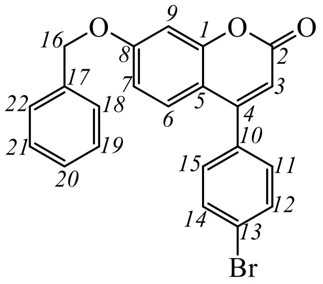

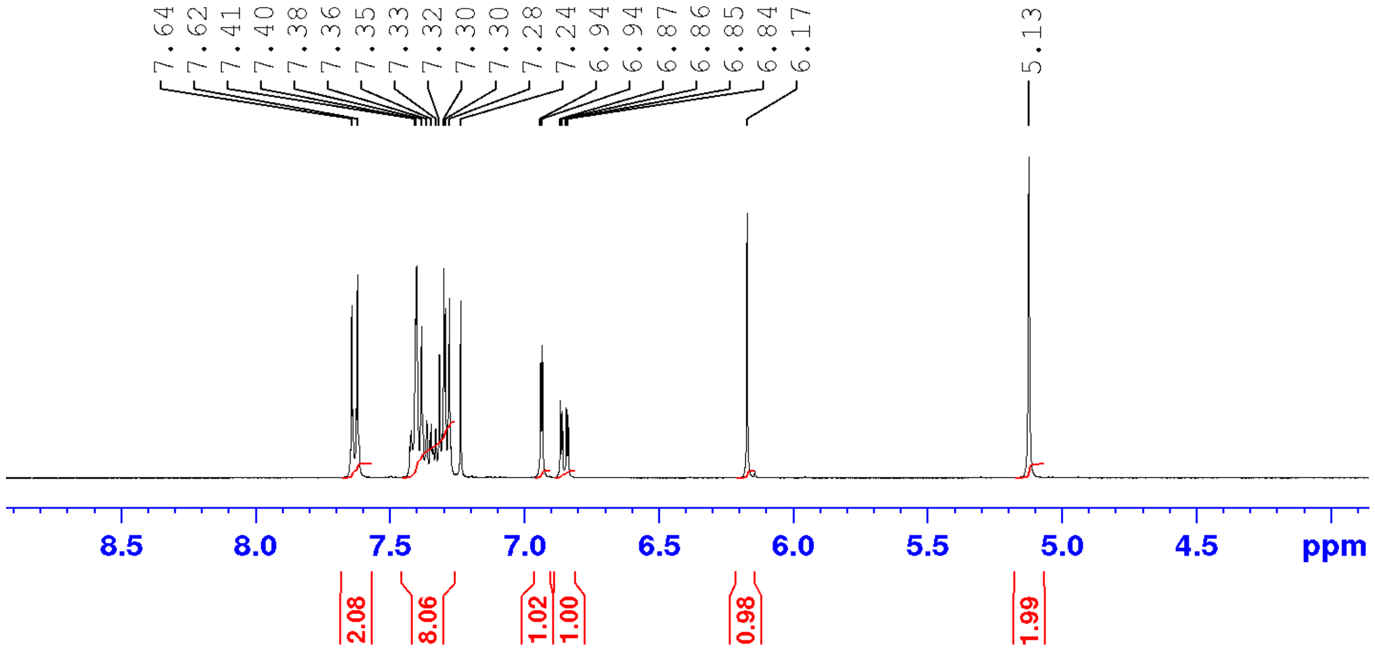

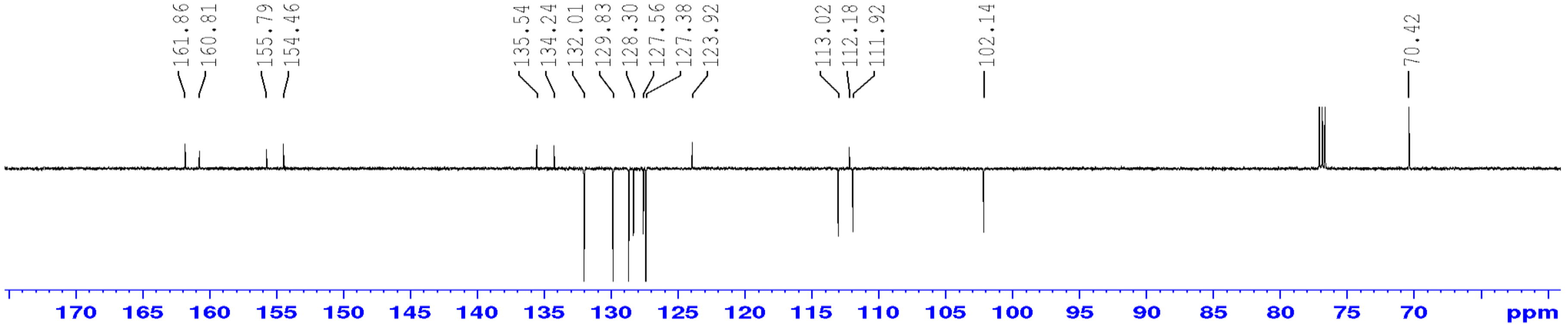

Compound **16a**

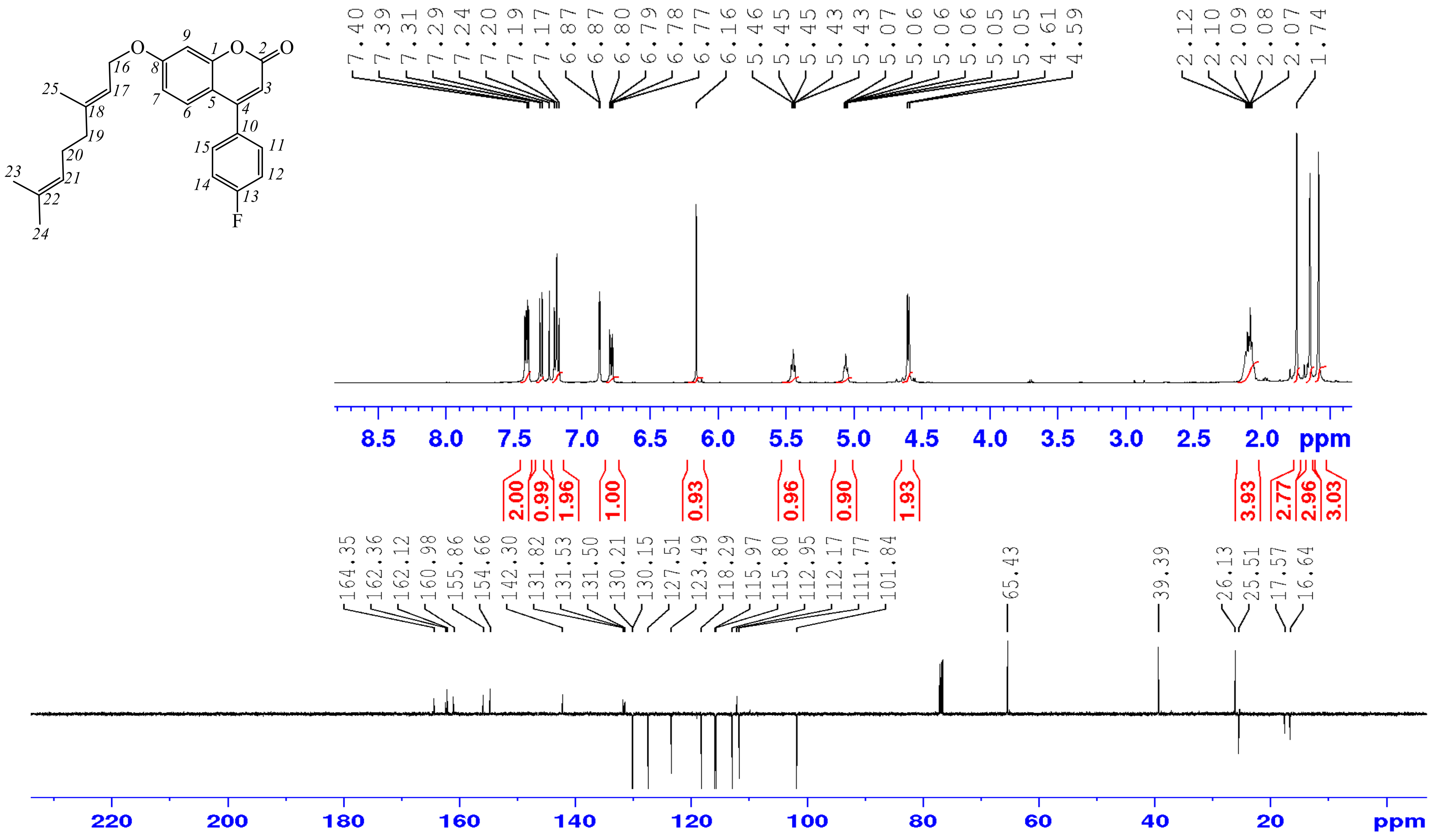

Compound **16b**

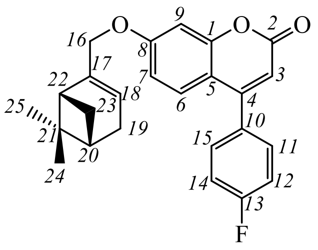

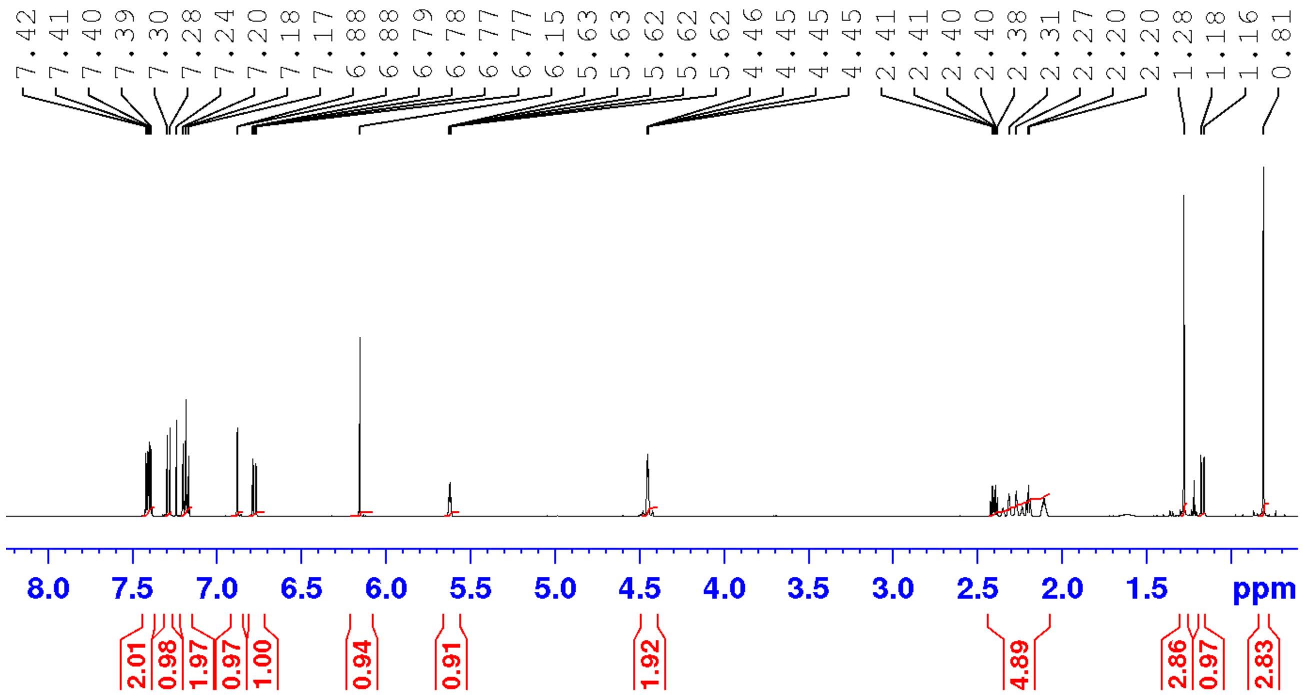

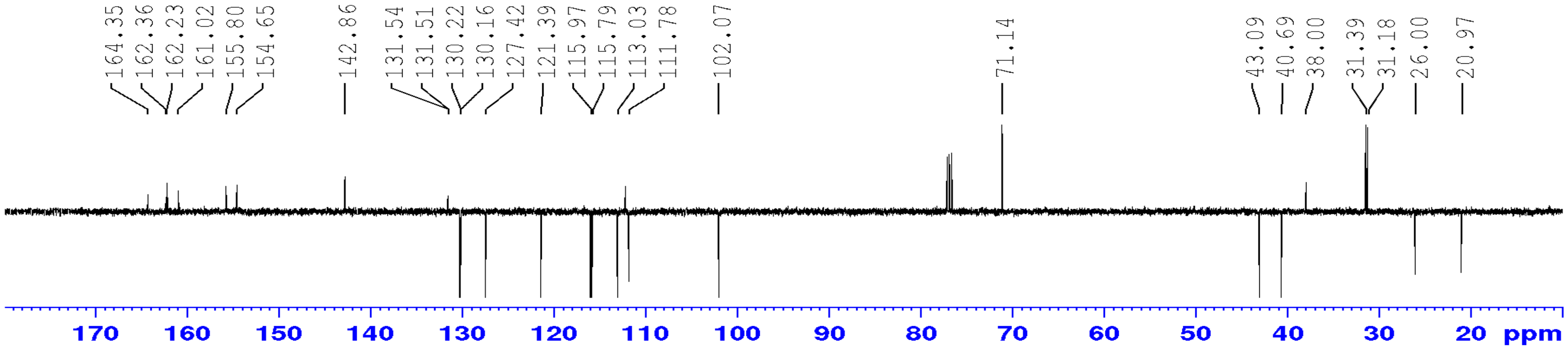

Compound **16e**

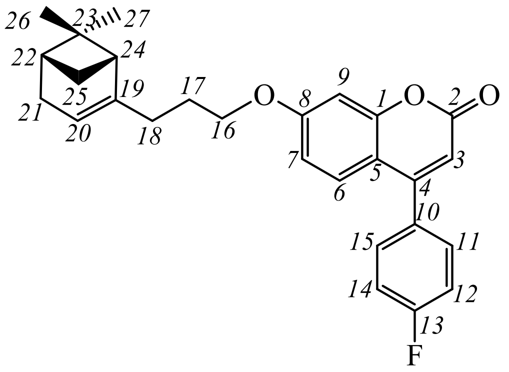

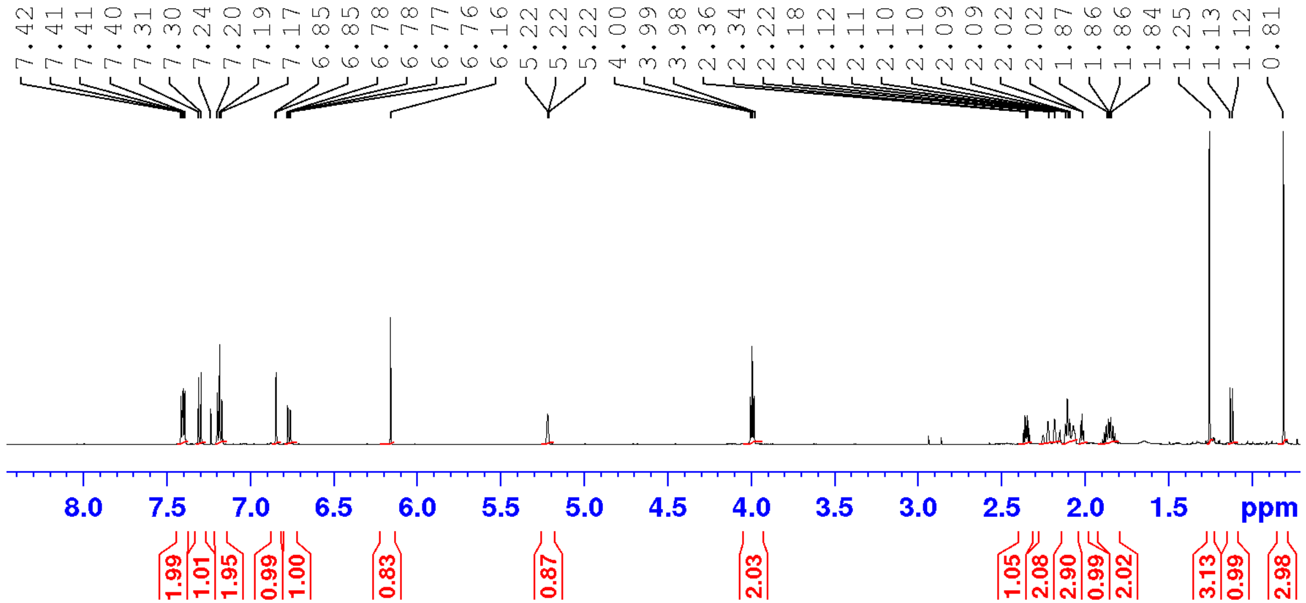

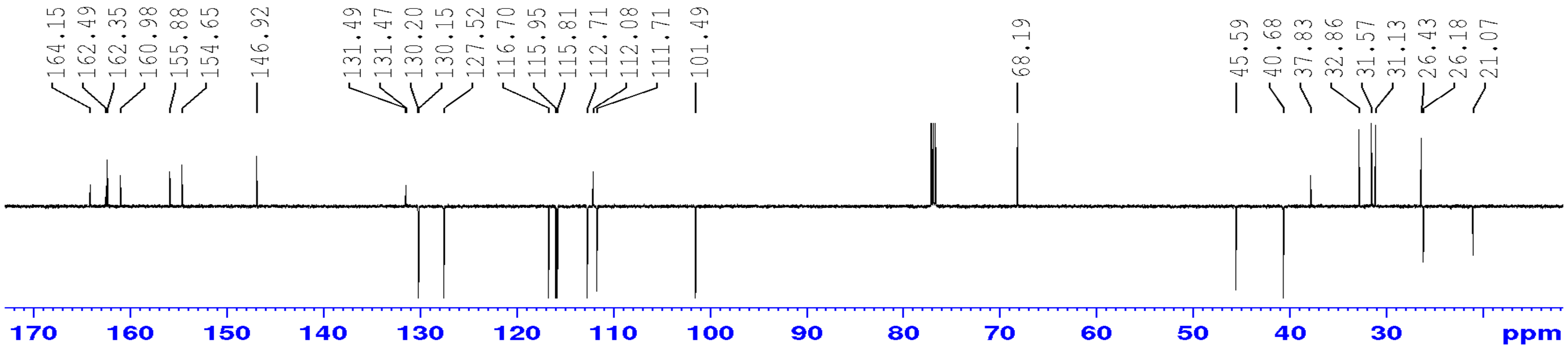

Compound **16f**

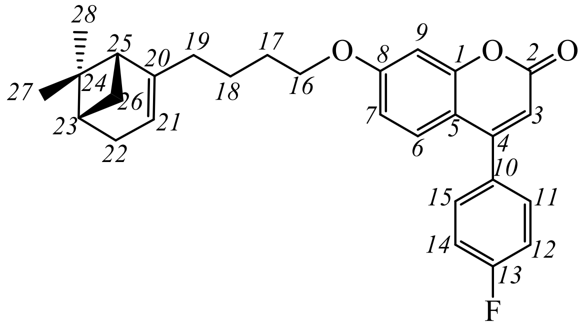

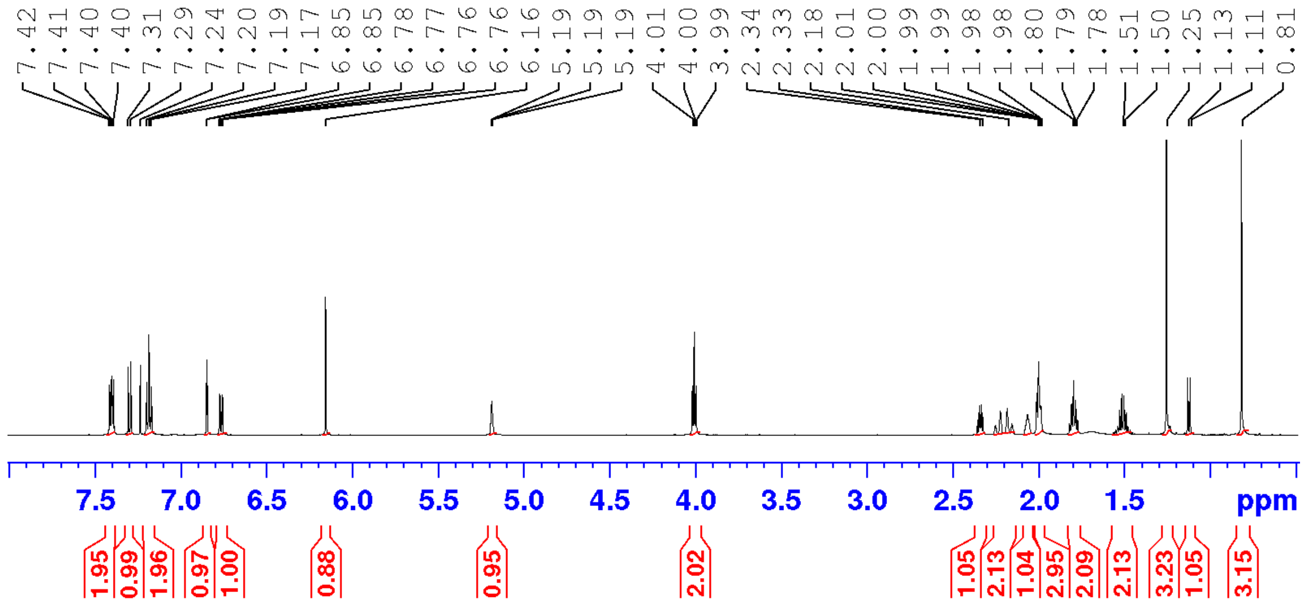

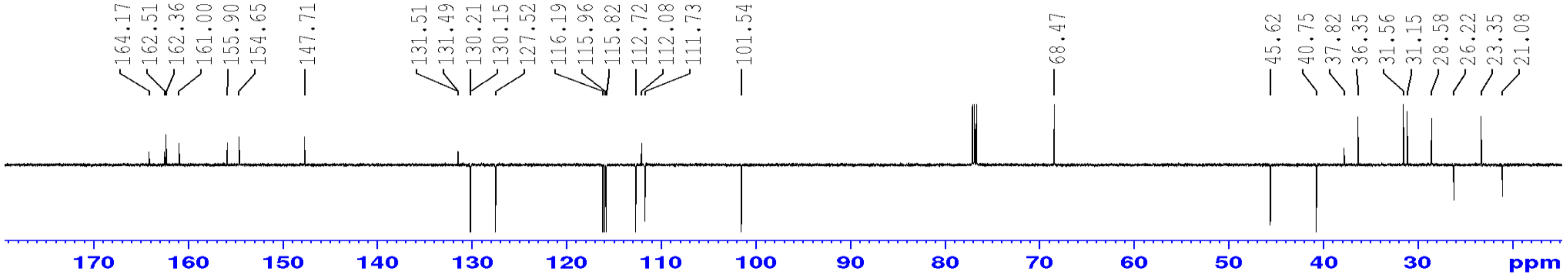

Compound **16g**

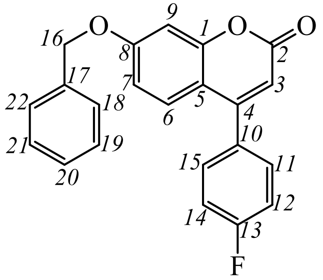

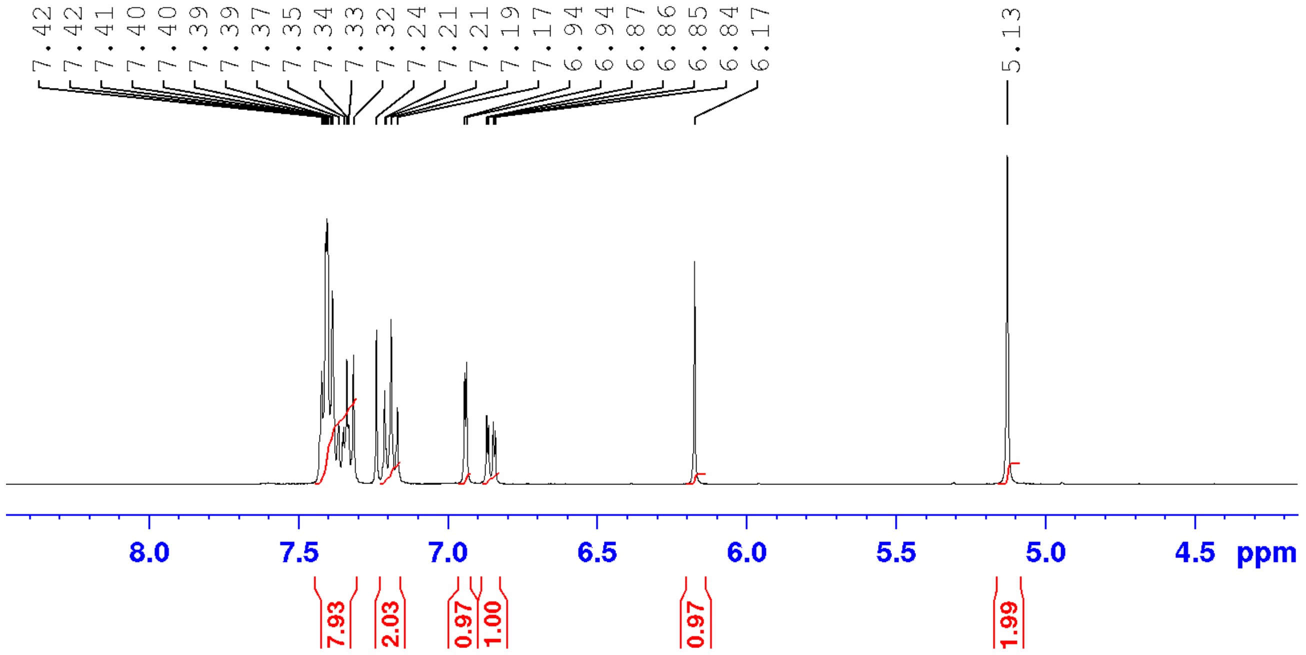

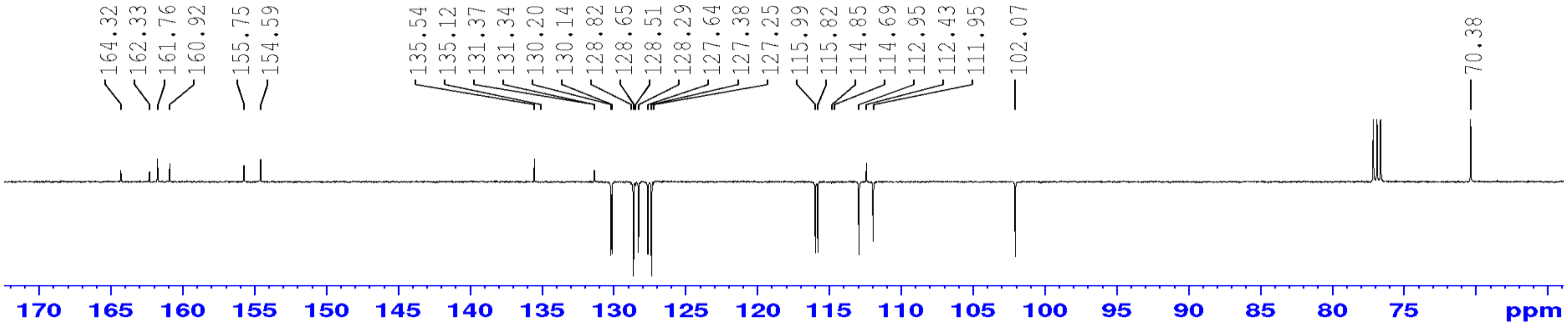

Compound **17a**

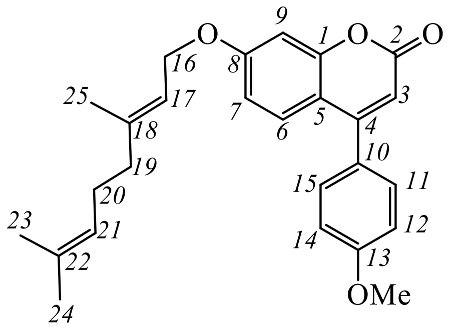

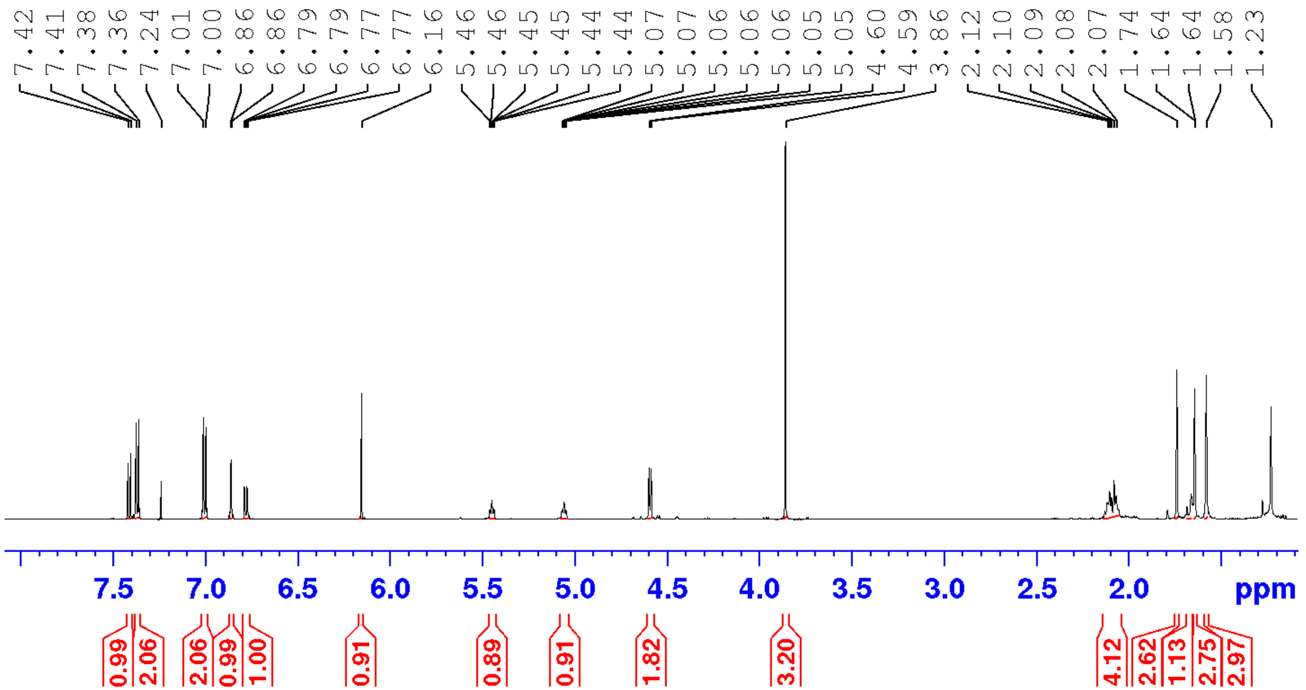

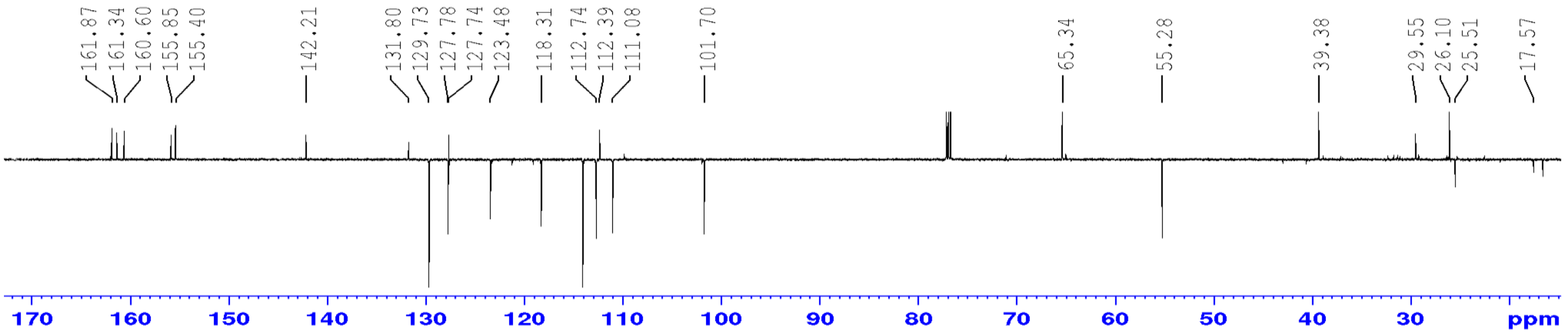

Compound **17b**

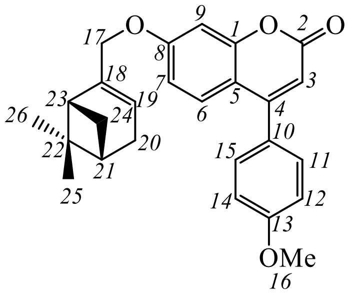

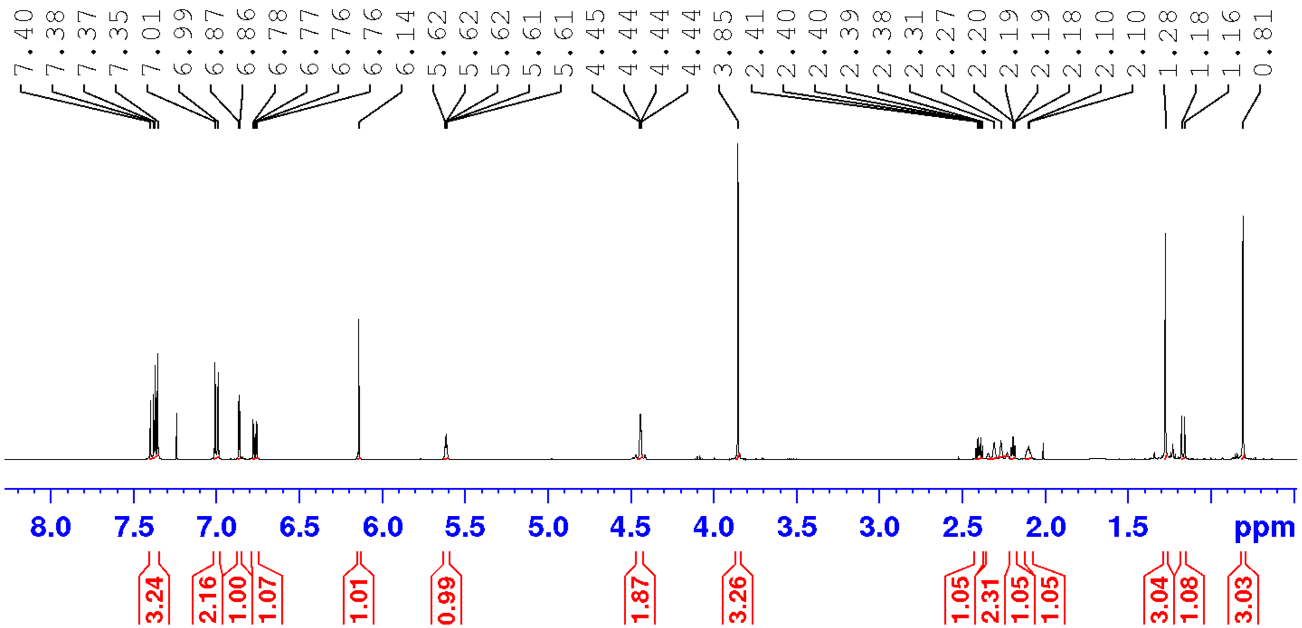

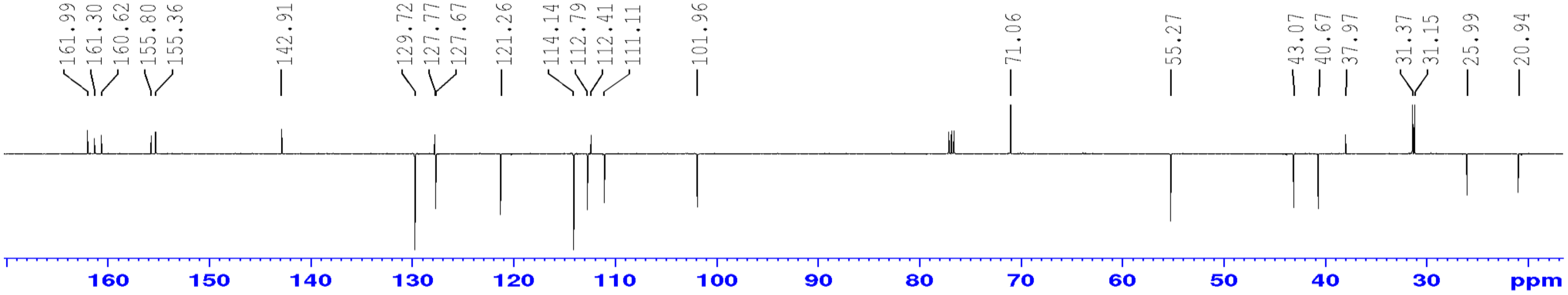

Compound **17d**

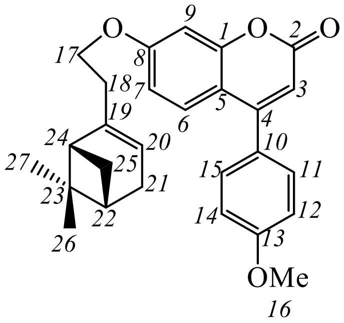

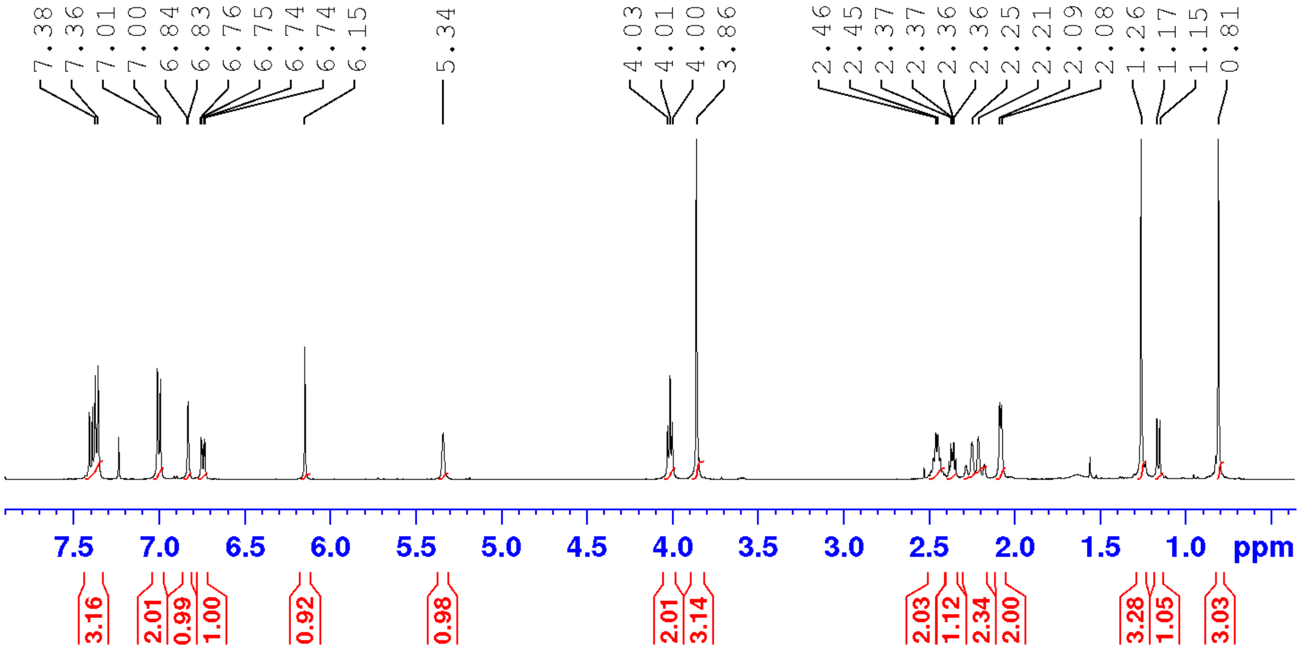

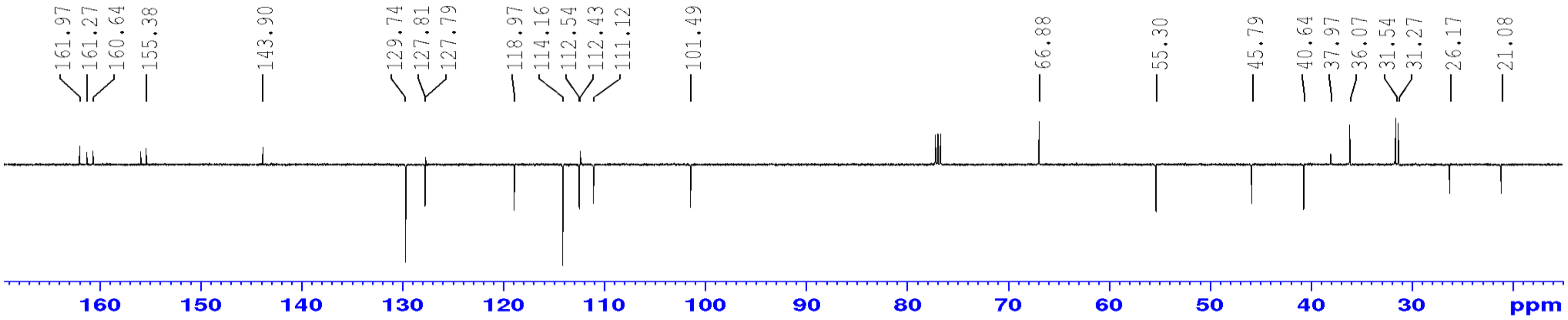

Compound **17e**

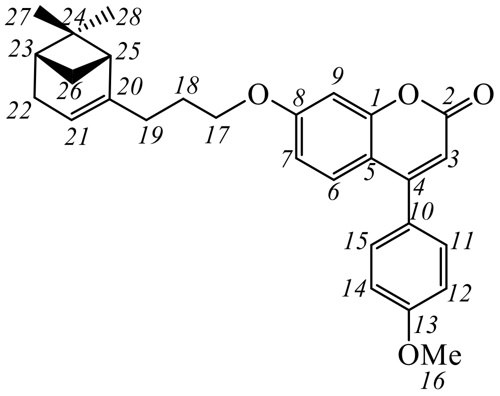

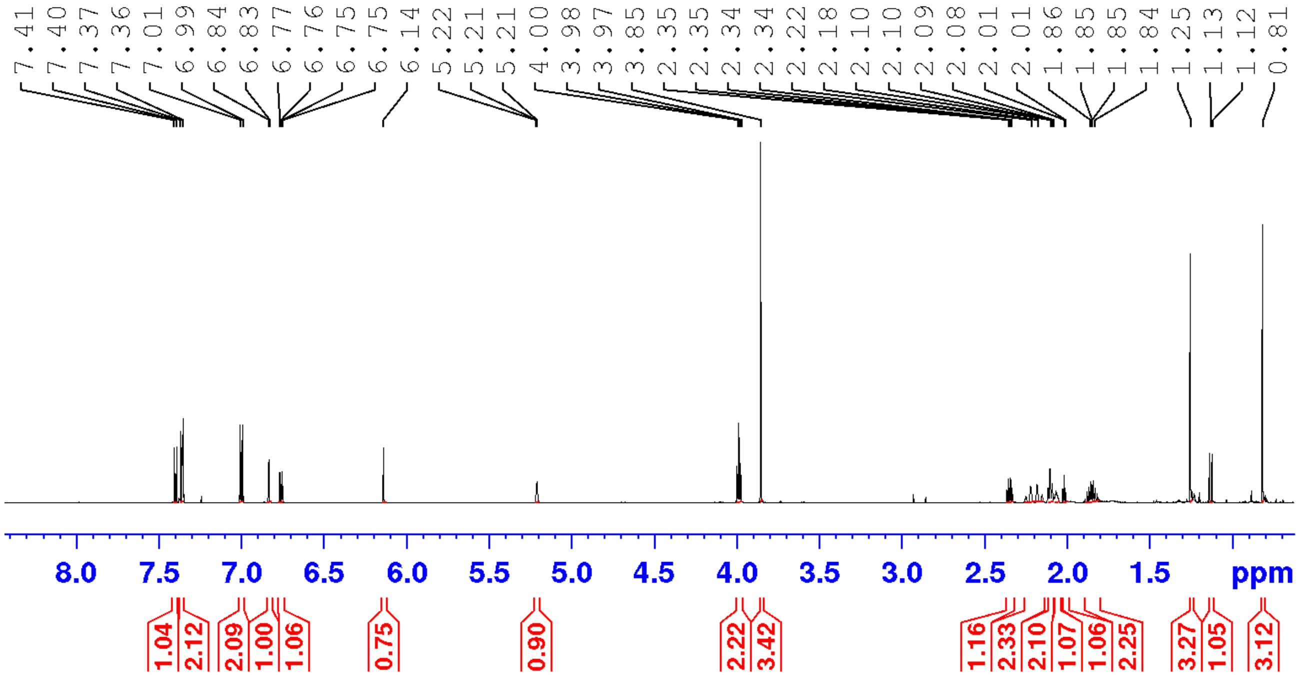

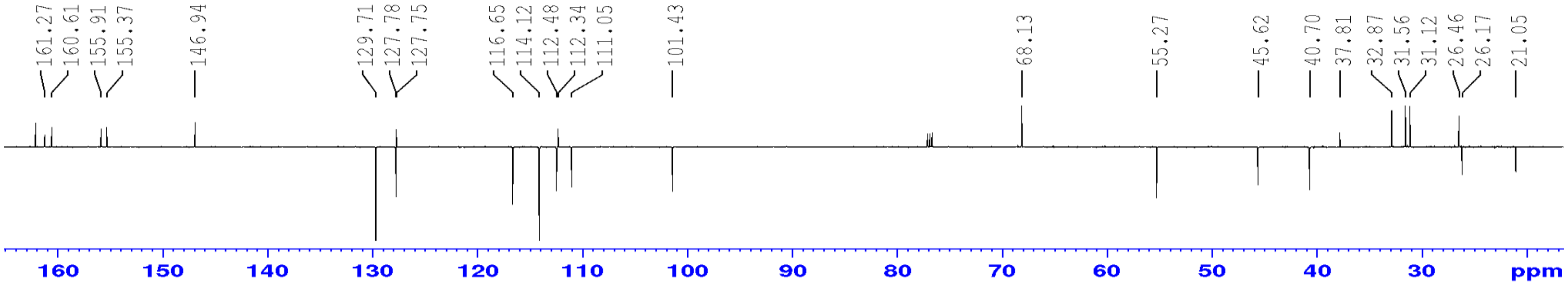

Compound **17f**

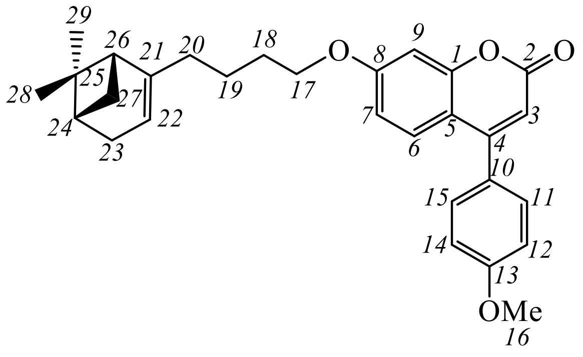

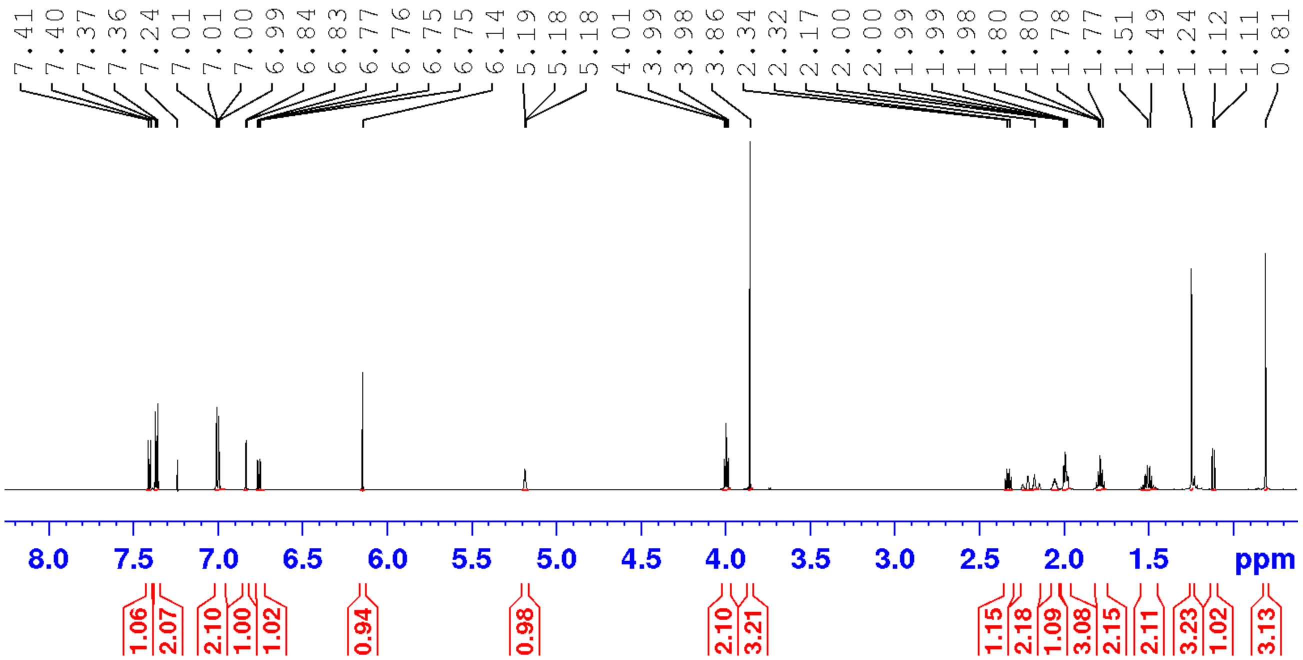

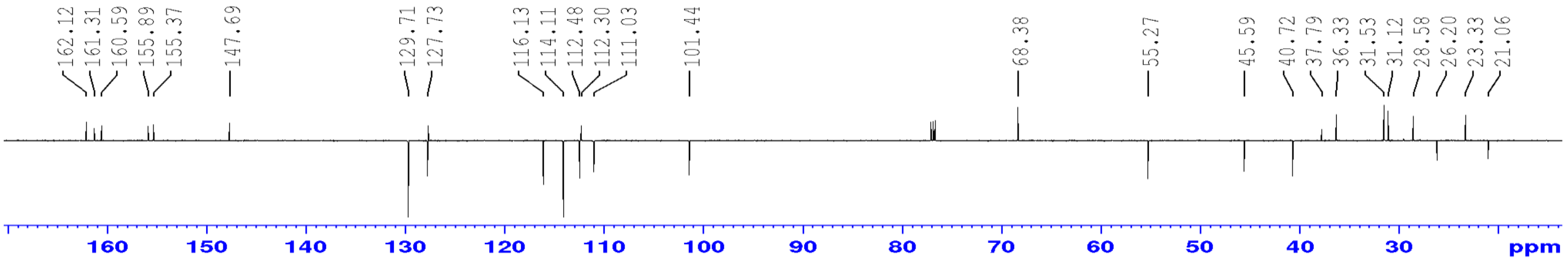

Compound **17g**

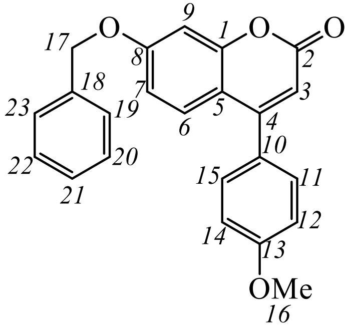

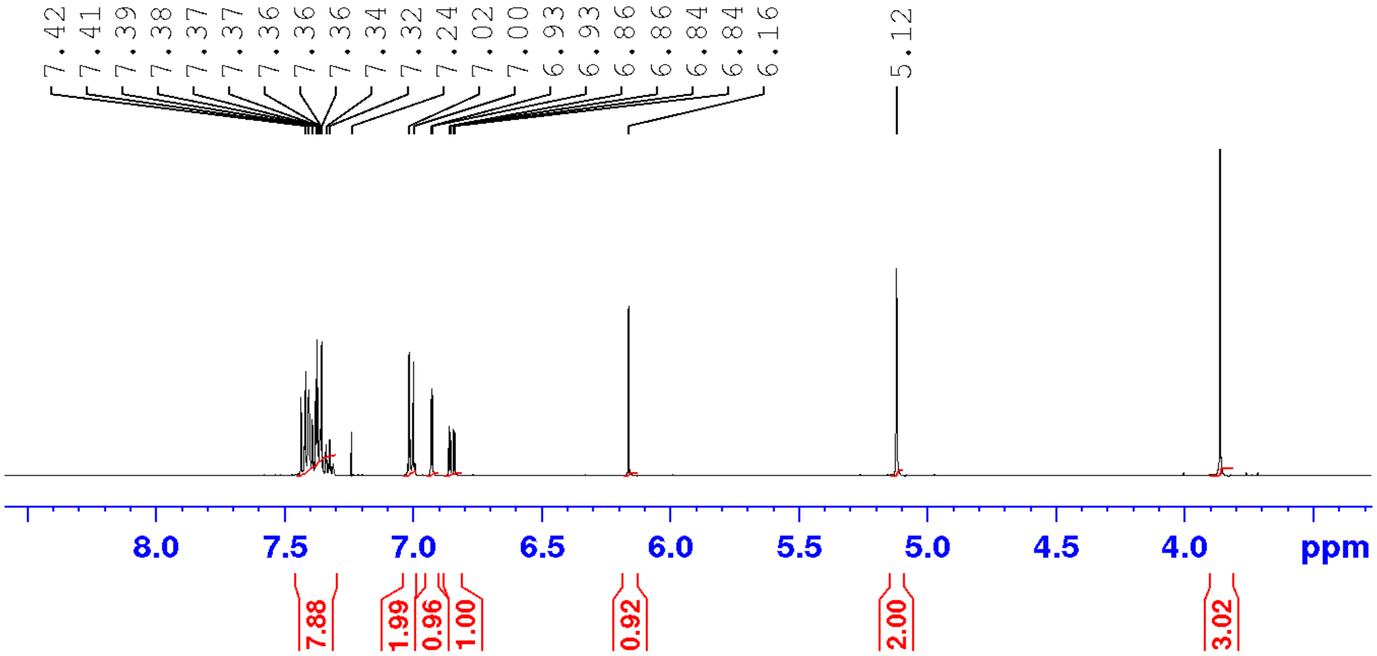

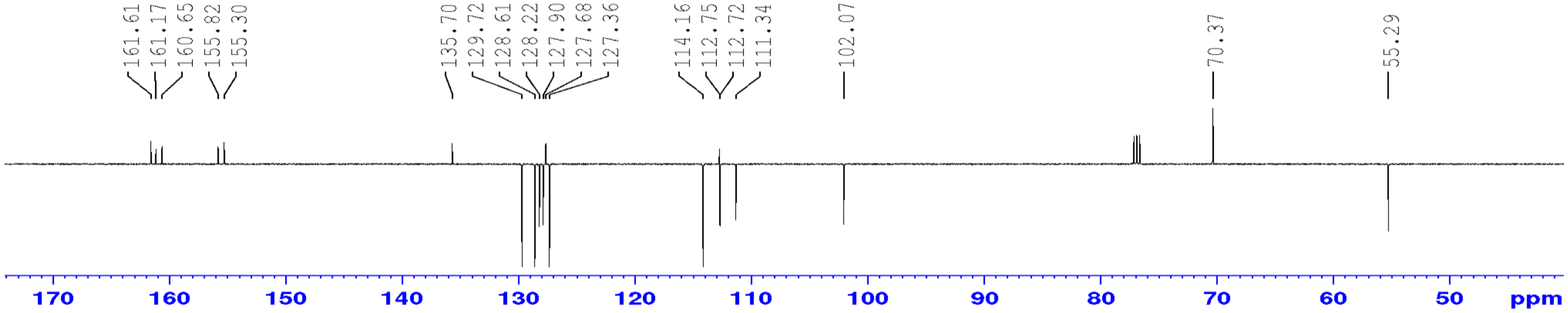

Compound **21**

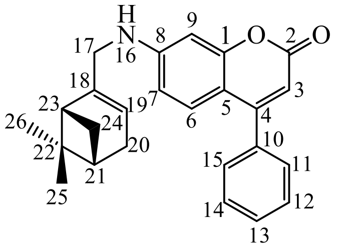

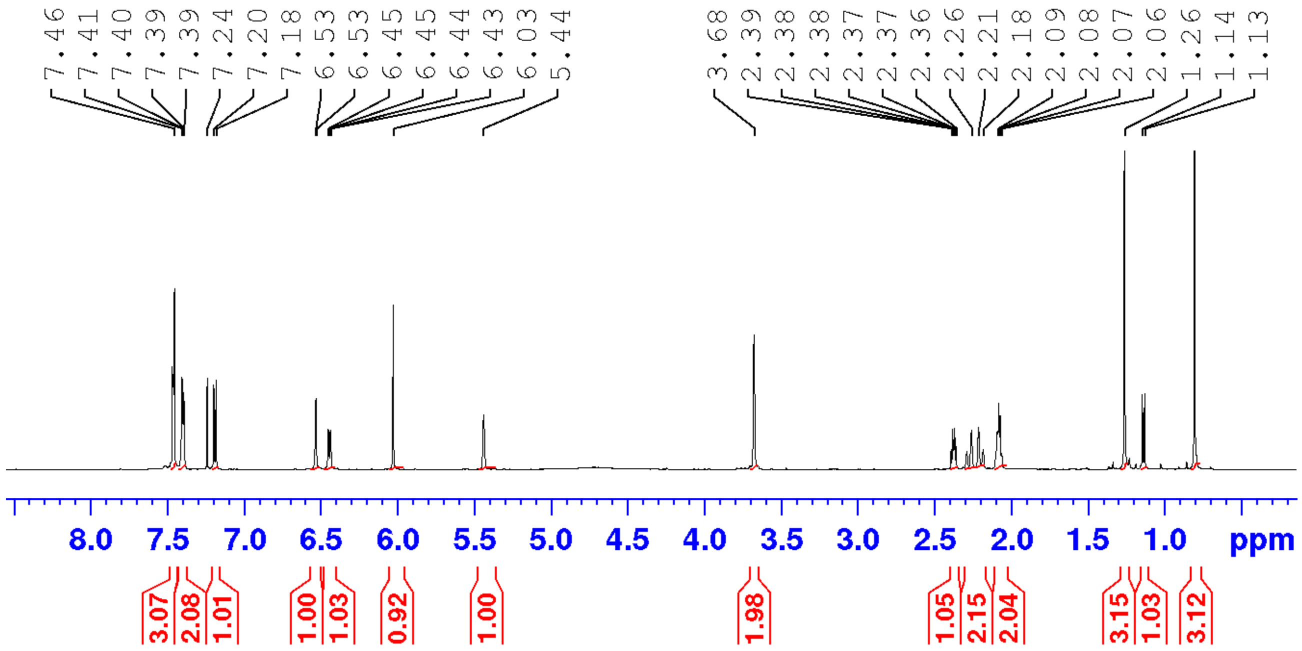

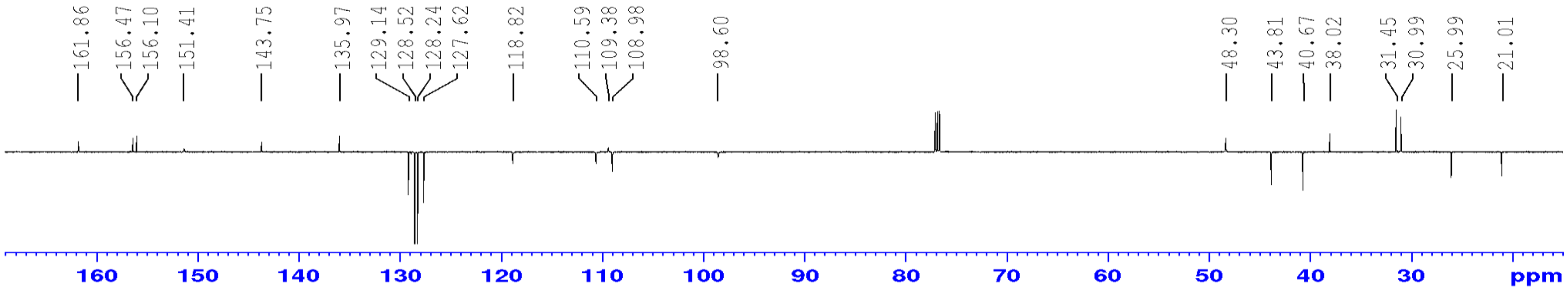

Compound **22**

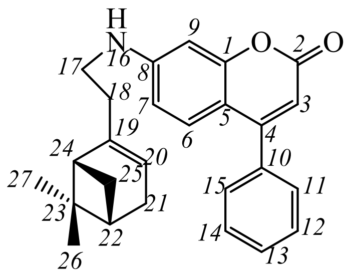
H
N
O
O
1 2 3 4 5 6 7 8 9 10 11 12 13 14 15 16 17 18 19 20 21 22 23 24 25 26 27

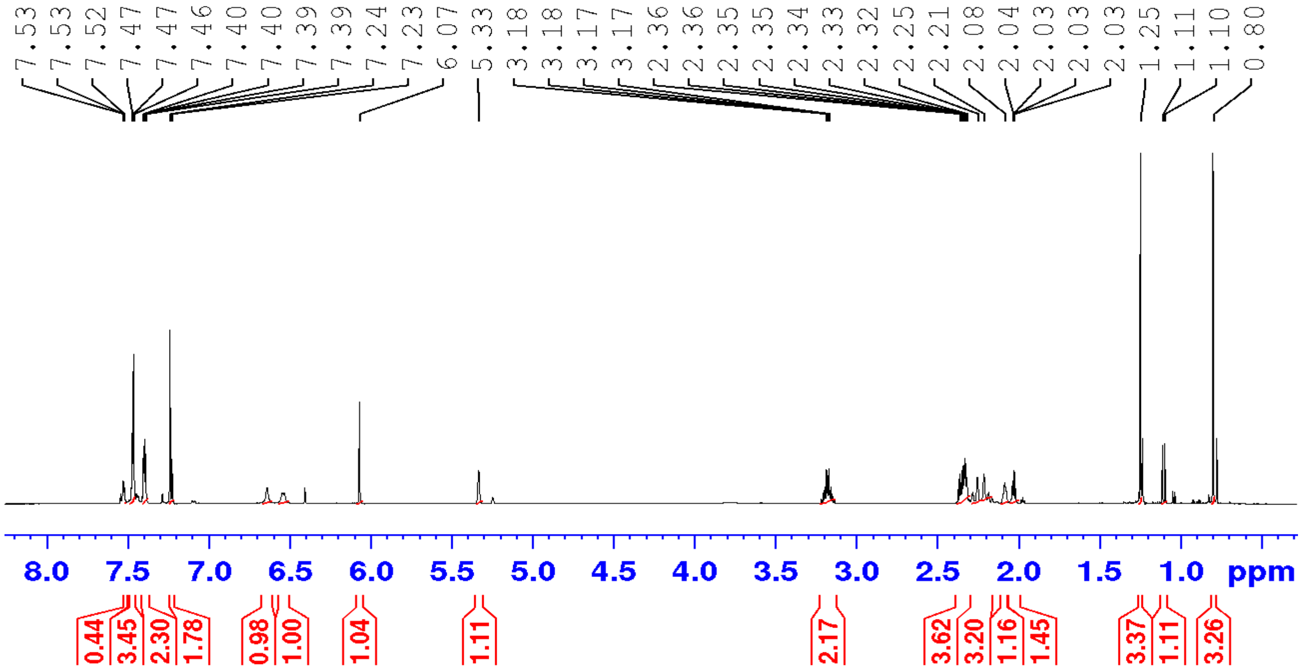
7.53 7.53 7.52 7.47 7.47 7.46 7.40 7.40 7.39 7.39 7.24 7.23 6.07 5.33 3.18 3.18 3.17 3.17 2.36 2.36 2.35 2.35 2.34 2.33 2.32 2.25 2.21 2.08 2.04 2.03 2.03 2.03 1.25 1.11 1.10 0.80
8.0 7.5 7.0 6.5 6.0 5.5 5.0 4.5 4.0 3.5 3.0 2.5 2.0 1.5 1.0 ppm
0.44 3.45 2.30 1.78 0.98 1.00 1.04 1.11 2.17 3.62 3.20 1.16 1.45 3.37 1.11 3.26

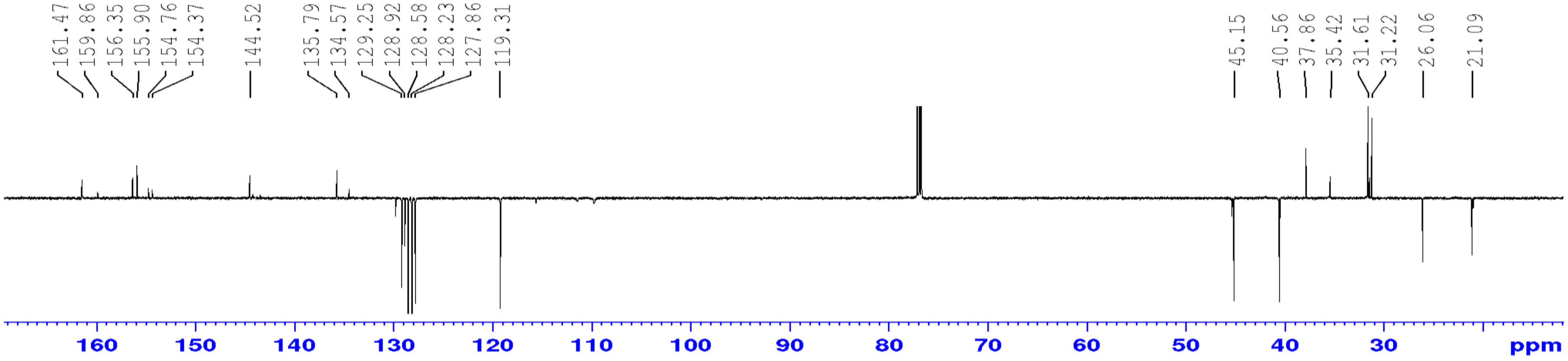
161.47 159.86 156.35 155.90 154.76 154.37 144.52 135.79 134.57 129.25 128.92 128.58 128.23 127.86 119.31 45.15 40.56 37.86 35.42 31.61 31.22 26.06 21.09
160 150 140 130 120 110 100 90 80 70 60 50 40 30 ppm

Correspondence of ligands' pharmacophores to the pharmacophore features of the binding site

The structural descriptors of the studied ligands are presented in Tables SM-1-3: M – molecular weight, AlogP – the predicted octanol/water partial coefficient based on atomic contributions shows the value of the lipophilicity of the compounds under consideration, PSA – polar surface area or the sum of the surface of all polar atoms, according to (Pajouhesh and Lenz 2005) PSA value greater than 140 $Å^2$ characteristic of compounds with poor membrane permeability, HBD and HBA are donor atoms and hydrogen bond acceptors, respectively. pIC_{50} – $-lg[IC_{50}]$: c; SI is the selectivity index, the ratio of cytotoxicity to inhibitory activity. Based on the SI value of the connection, the leaders are colored green. Matched ligand sites – the ratio of ligands to the pharmacophore profile of the binding site: A – the presence of a hydrogen bond acceptor; R – the presence of an aromatic ring; HP – the presence of a hydrophobic fragment; D – the presence of a fragment – a donor of the hydrogen bond.

Table S1. Analysis of structural descriptors and pharmacophoric features of the ligand as a function of linker length.

ID structures		-$(CH_2)_n$-	M, Da	AlogP	PSA, Å^2	HBD	HBA	Matched ligand sites	pIC_{50}	SI
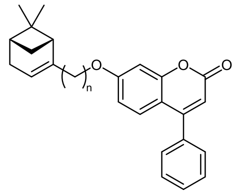	**14b**	1	372.5	5.65	39.44	0	3	3: **A – 1**; **R – 2**	4.75	**32.8**
	**14d**	2	386.5	5.97	39.44	0	3	3: **HP – 1**; **R – 2**	4.08	7.9
	**14e**	3	400.5	6.43	39.44	0	3	3: **HP – 1**; **R – 2**	**5.12**	**192.6**
	**14f**	4	414.5	6.89	39.44	0	3	< 3	3.95	**11.4**
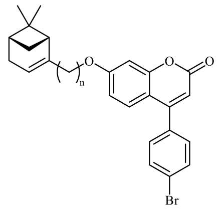	**15b**	1	451.2	6.40	39.44	0	3	3: **A – 1**; **R – 2**	4.82	3.0
	**15d**	2	465.4	6.72	39.44	0	3	3: **HP – 1**; **R – 2**	< 4.7	< 0.8
	**15e**	3	479.4	7.18	39.44	0	3	3: **HP – 1**; **R – 2**	< 4.9	< 1
	**15f**	4	493.4	7.64	39.44	0	3	< 3	5.02	9.6
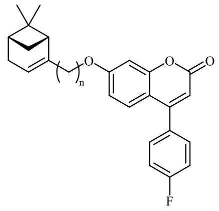	**16b**	1	390.5	5.86	39.44	0	3	3: **A – 1**; **R – 2**	3.99	2.9
	**16e**	3	418.5	6.64	39.44	0	3	3: **HP – 1**; **R – 2**	3.96	9.0
	**16f**	4	432.5	7.09	39.44	0	3	< 3	5.03	**11.8**
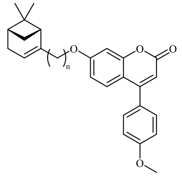	**17b**	1	402.5	5.64	48.67	0	4	3: **A – 1**; **R – 2**	< 4.9	< 1
	**17d**	2	416.5	5.96	48.67	0	4	3: **HP – 1**; **R – 2**	4.19	**10.6**
	**17f**	4	444.5	6.87	48.67	0	4	3: **HP – 1**; **R – 2**	< 4.9	< 1
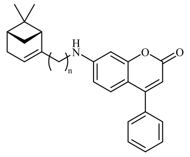	**21**	1	371.5	5.48	42.24	1	2	4: **D – 1**; **HP – 1**; **R – 2**	< 4.9	< 1.5
	**22**	2	385.5	5.80	42.24	1	2	3: **HP – 1**; **R – 2**	4.10	6.0

Table S2. Analysis of structural descriptors and pharmacophoric features of the ligand as a function of the nature of the substituent.

ID structures		-R	M, Da	AlogP	PSA	HBD	HBA	pIC_{50}	SI	Matched ligand sites
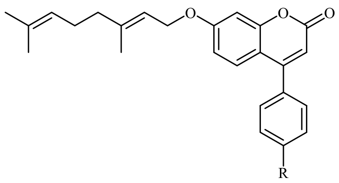	**14a**	-H	374.5	6.81	39.44	0	3	4.17	11.9	< 3
	**15a**	-Br	453.4	7.55	39.44	0	3	5.05	1.0	< 3
	**16a**	-F	392.5	7.01	39.44	0	3	4.32	5.8	< 3
	**17a**	$-OCH_3$	404.5	6.79	48.67	0	4	4.23	5.8	< 3
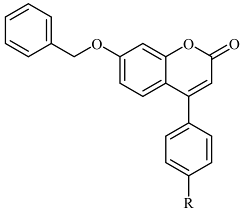	**14g**	-H	328.4	5.10	39.44	0	3	< 2.80	< 0.9	< 3
	**15g**	-Br	407.3	5.85	39.44	0	3	4.08	3.2	3: **R – 3**
	**16g**	-F	346.4	5.30	39.44	0	3	3.16	< 0.9	3: **R – 3**
	**17g**	$-OCH_3$	358.4	5.08	48.67	0	4	< 4.77	< 1	3: **R – 3**

Table S3. Analysis of structural descriptors and pharmacophoric features of the different stereoisomers.

ID structures		-R<br>-R’	M, Da	AlogP	PSA	HBD	HBA	pIC_{50}	SI	Matched ligand sites
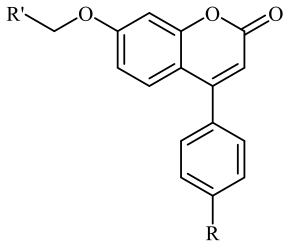	**14b**	R = H<br>R’ = 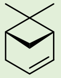	372.5	5.65	39.44	0	3	4.75	32.8	3: **A – 1**; **R – 2**
	**14c**	R’ = 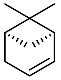	372.5	5.65	39.44	0	3	3.63	3.5	< 3
	**15b**	R = Br<br>R’ = 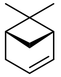	451.2	6.40	39.44	0	3	4.82	3.0	3: **A – 1**; **R – 2**
	**15c**	R’ = 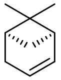	451.2	6.40	39.44	0	3	4.31	1.9	< 3
	**16b**	R = F<br>R’ = 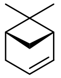	390.5	5.86	39.44	0	3	3.99	2.9	3: **A – 1**; **R – 2**
	**16c**	R’ = 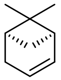	390.5	5.86	39.44	0	3	4.84	1.4	< 3
	**17b**	R = -<br>OCH_3<br>R’ = 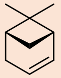	402.5	5.64	48.67	0	4	< 4.9	< 1	3: **A – 1**; **R – 2**
	**17c**	R’ = 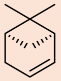	402.5	5.64	48.67	0	4	< 4.8	< 0.98	< 3

Molecular docking

Table S4. Molecular docking results.

ID *compounds*	pIC50	Pose (max 20)	GC	IDF	H-bond	Other interactions
14a	4.17	17	-8.70	-3108.00	None	A: Phe488 – π-π<br>A: Phe140 – HP<br>B: Phe140 – π-π<br>D: Phe137; Leu138; Met396; Phe488 – HP<br>D: Phe140 – π-π
14b	4.75	12	-9.27	-3108.97	None	A: Phe488 – π-π<br>B: Phe488 – π-π<br>B: Phe140; Leu141 – HP<br>D: Phe488 – π-π
14c	3.63	9	-8.31	-3106.42	None	A: Phe488 – π-π<br>B: Phe488 – π-π<br>D: Phe137; Leu138; Phe140; Leu141 – HP
14d	4.08	16	-9.05	-3106.64	None	D: Phe488 – π-π<br>D: Phe137; Leu138; Phe140; Leu141 – HP
14e	5.12	17	-10.26	-3108.15	None	A: Phe140 – HP<br>B: Phe140 – π-π<br>B: Phe488 – π-π<br>B: Phe137; Leu138; Phe140; Leu141 – HP<br>D: Phe137; Leu138; Phe140; Leu141 – HP
14f	3.95	17	-9.22	-3107.32	None	A: Phe488 – π-π<br>B: Phe488 – π-π
14g	2.80	8	-7.60	-3104.82	None	A: Phe140; Phe488 – π-π<br>A: Met396 – HP<br>D: Phe488 – π-π
15a	5.05	17	-9.40	-3110.11	None	A: Phe488 – π-π<br>A: Phe137; Phe140 – HP

						B: Phe140; Leu141 – HP<br>B: Phe488 – π-π<br>D: Phe140; Phe488 – π-π
15b	4.82	14	-9.70	-3108.88	None	A: Phe488 – π-π<br>A: Phe137; Phe140 – HP<br>B: Phe140; Leu141 – HP<br>B: Phe488 – π-π<br>D: Phe140; Phe488 – π-π
15c	4.30	19	-8.14	-3106.38	A: Asp489	D: Phe137; Leu138; Phe140 – HP
15d	4.70	16	-9.47	-3110.81	A: Phe488<br>Asp489	A: Phe137 – HP<br>B: Phe488 – π-π<br>B: Phe137; Leu138; Phe140 – HP
15e	4.90	12	-8.73	-3109.03	A: Phe488<br>Asp489	A: Phe488 – π-π<br>B: Phe137; Leu138; Phe140 – HP
15f	5.02	12	-9.72	-3110.20	A: Phe488<br>D: Asp486	A: Phe140 – HP<br>B: Phe488 – HP<br>D: Phe137, Phe138 – HP.<br>D: Phe488 – π-π
15g	4.08	8	-8.99	-3109.05	None	A: Phe488 – π-π<br>B: Phe140; Phe488 – π-π<br>B: Phe140; Leu141 – HP<br>D: Phe140; Phe488 – π-π
16a	4.32	15	-8.86	-3108.25	A: Asp489<br>Phe488	A: Lys498 – π-cation<br>B: Phe137; Phe140; Phe488 – HP<br>D: Phe140; Phe488 – HP
16b	3.99	17	-9.03	-3107.49	None	A: Phe488 – π-π<br>D: Phe488 – π-π<br>D: Phe137; Leu138; Phe140 – HP
16c	4.84	6	-9.07	-3109.40	None	A: Phe488 – π-π<br>A: Phe137; Phe140 – HP<br>B: Phe140; Leu141 – HP

						D: Phe140; Phe488 – π-π
16e	3.96	17	-9.20	-3107.86	None	A: Phe140 – π-π<br>A: Phe140 – HP<br>B: Met396 – HP<br>B: Phe140 – π-π<br>D: Phe137; Leu138; Phe140 – HP
16f	5.03	13	-9.99	-3108.68	None	A: Phe488 – π-π<br>B: Phe140 – π-π<br>D: Phe140 – π-π<br>D: Phe137; Leu138; Phe140 – HP
16g	3.20	11	-8.17	-3107.30	None	A: Phe140; Phe488 – π-π<br>B: Phe488 – π-π<br>D: Phe488 – π-π<br>D: Phe137; Leu138; Phe140 – HP
17a	4.20	13	-8.97	-3109.54	A: Asp489<br>Phe488	B: Phe488 – π-π<br>B: Phe137; Leu138; Phe140 – HP
17b	4.90	14	-9.16	-3108.81	None	B: Phe140; Phe488 – π-π<br>B: Phe137; Leu138; Phe140 – HP<br>D: Phe137; Leu138; Phe140 – HP
17c	4.80	10	-9.59	-3109.31	None	A: Phe140; Phe488 – π-π<br>B: Phe137; Leu138; Phe140 – HP<br>D: Phe140; Phe488 – π-π<br>D: Phe137; Leu138; Phe140; Met396 – HP
17d	4.19	18	-8.53	-3107.42	A: Phe488<br>A: Asp489	A: Lys498 – π-cation<br>D: Phe488 – π-π<br>D: Phe137; Leu138; Phe140; Leu141 – HP
17f	4.97	18	-9.86	-3110.34	A: Phe488<br>A: Asp489<br>B: Lys498	B: Pro484 – HP<br>D: Phe488 – HP

17g	4.80	11	-9.72	-3109.89	None	A: Phe488 – π-π<br>B: Phe488 – π-π<br>D: Phe137; Leu138; Phe140 – HP
21	4.90	16	-10.36	-3106.98	B: Phe137	A: Phe488 – π-π<br>B: Phe488 – π-π<br>B: Phe137; Leu138; Phe140 – HP
22	4.10	17	-8.86	-3106.99	None	A: Phe140 – HP<br>A: Phe488 – π-π<br>B: Phe488 – π-π<br>D: Phe137; Leu138; Phe140; Leu141 – HP<br>D: Phe140 – π-π

Molecular dynamic

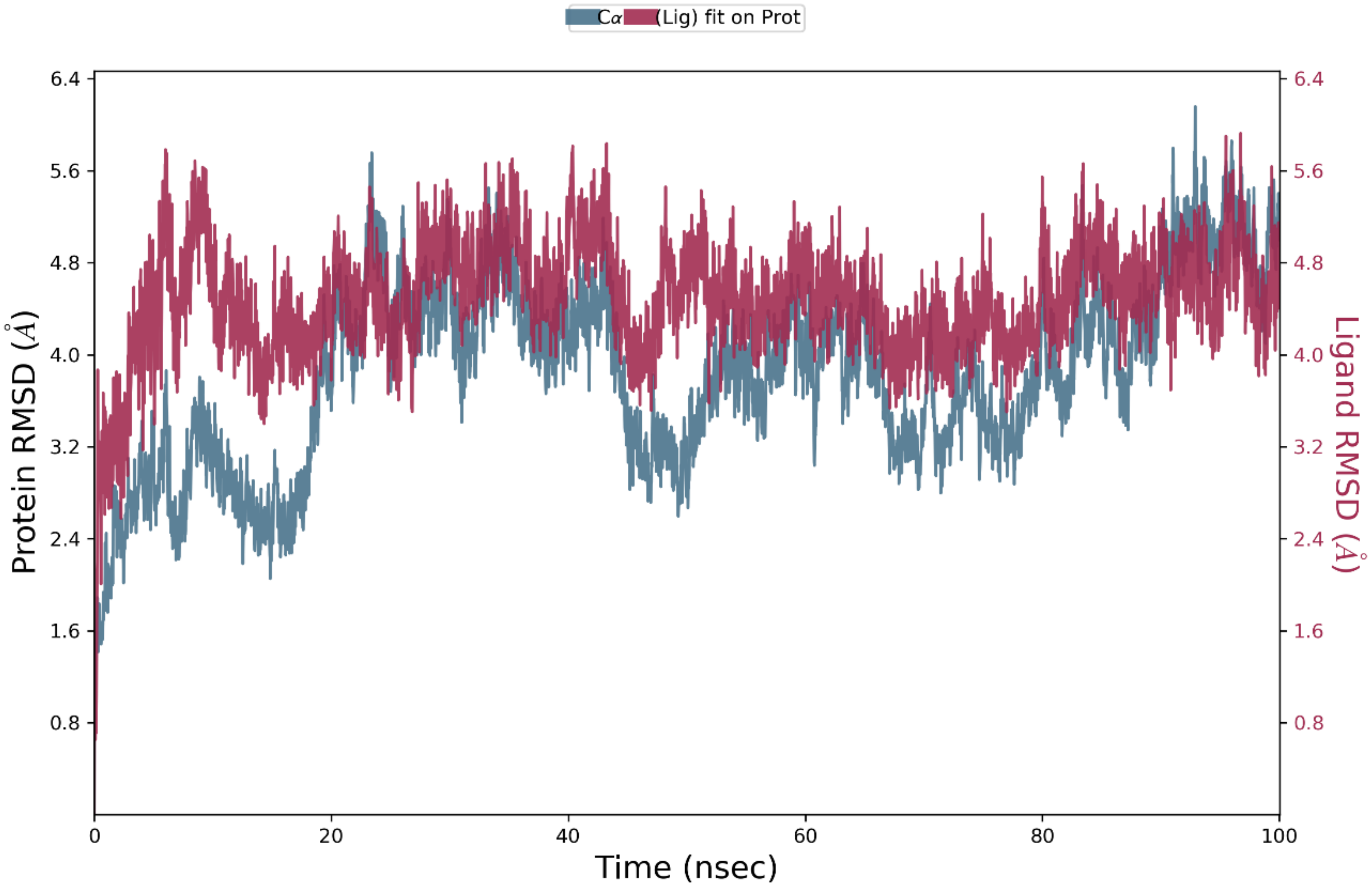

Figure S1. RMSD fluctuations.

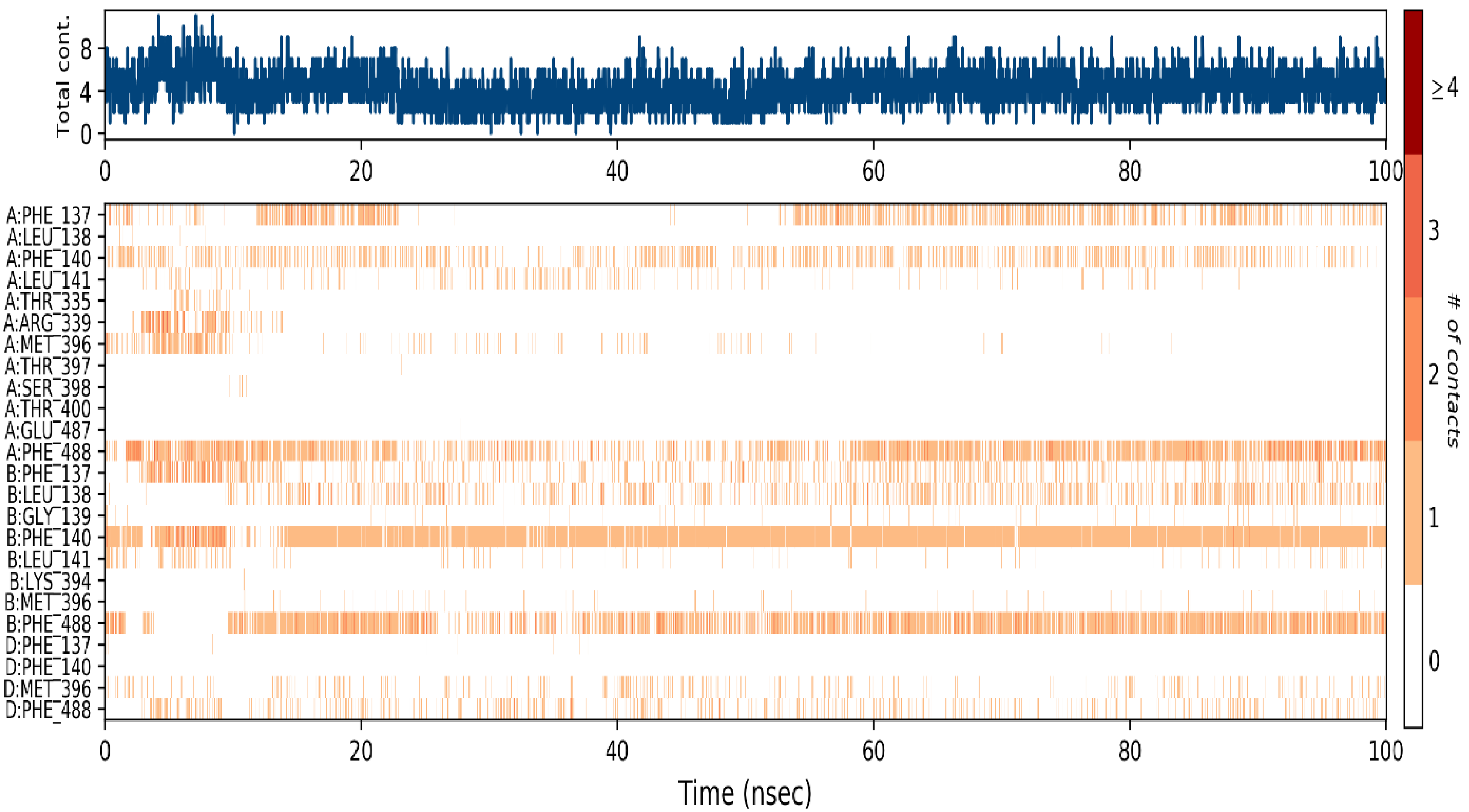

Figure S2. The total number of protein-ligand contacts.
